# Current-induced creation and dynamics of embedded magnetic skyrmion bags

**DOI:** 10.1038/s41467-026-74046-4

**Published:** 2026-06-11

**Authors:** Yaodong Wu, Jialiang Jiang, Lingyao Kong, Meng Shi, Shouguo Wang, Mingliang Tian, Haifeng Du, Jin Tang

**Affiliations:** 1 School of Physics and Materials Engineering, Hefei Normal University, Hefei, China; 2 State Key Laboratory of Opto-Electronic Information Acquisition and Protection Technology, School of Physics, Anhui University, Hefei, China; 3 Anhui Provincial Key Laboratory of Low-Energy Quantum Materials and Devices, High Magnetic Field Laboratory, HFIPS, Chinese Academy of Sciences, Hefei, China; 4 Anhui Provincial Key Laboratory of Magnetic Functional Materials and Devices, School of Materials Science and Engineering, Anhui University, Hefei, China

**Keywords:** Spintronics, Spintronics

## Abstract

Magnetic skyrmion bags—vortex-like structures hosting multiple skyrmions with tunable topological charge (*Q*)—hold significant promise for next-generation spintronic computing. However, while their creation using magnetic fields has been demonstrated, their direct electrical generation remains an outstanding challenge. Here, we report the direct current-induced formation and manipulation of embedded skyrmion bags in a FeGe nanoplate under zero magnetic field. Using in-situ Lorentz transmission electron microscopy, we capture the transformation of a distorted helical ground state into embedded skyrmion bags with diverse configurations, driven by nanosecond current pulses. Theoretical analysis indicates that this process is driven by the spin-transfer-torque-induced fracture of the helical state. Furthermore, we demonstrate electrically-induced transitions between skyrmion bags of different *Q*, leading to the stabilization of complex three-dimensional topological structures, including experimental signatures of magnetic monopoles and bobbers. Our work establishes a foundation for all-electrical control of high-*Q* topological spin textures and topological defects, paving the way for their application in functional spintronic devices.

## Abstract (web summary)

This study reports the all-electrical creation and manipulation of magnetic skyrmion bags at zero magnetic field, providing contribution to future topology-base spintronic applications.

## Introduction

Charge quantization of nuclei is the fundamental law of physics, giving rise to the colorful world we live in. In magnetic materials, the magnetization of magnetic structures can be quantified using the invariant known as the topological charge *Q*, which essentially counts the number of times the physical space covers the unit sphere that the magnetization vector **m** points to^1^. Likewise, the quantization of topological charges determines various topological spin textures, such as magnetic skyrmions^2–6^, magnetic bobbers^7^, and magnetic merons^8^. Topological spin textures with emergent electromagnetic properties are promising information carriers applied in spintronic devices^9–15^. Among these topological spin textures, magnetic skyrmions were the first to be discovered^3^, characterized by their unit *Q*, whose value depends on the specific magnetic arrangement configuration, either being *Q* = 1 or −1^4–6,16^. Subsequent advancements have employed skyrmions and counterparts as discrete building units to construct topological spin textures with customizable topological charges *Q*^17–22^, which may expand the potential applications of topological spintronic devices, including ASCII binary information encoding and interconnect devices^17,23^.

**Fig. 1:**
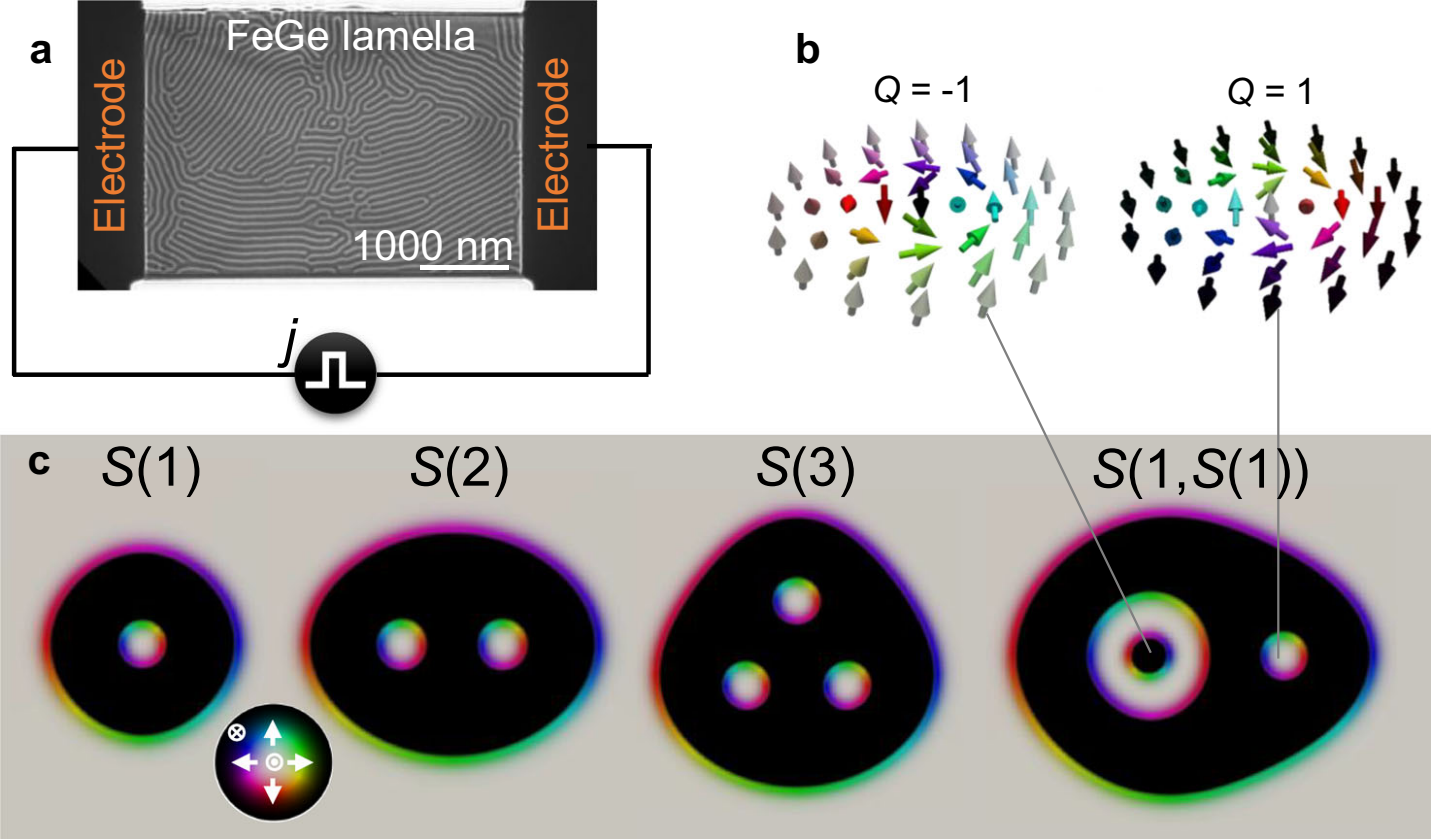
Magnetic configurations of skyrmion bags. **a** Schematic representation of the FeGe microdevice. **b** Skyrmions with *Q* = 1 and −1. **c** Representative examples of *S*(*n*) and *S*(*n*_1_, *S*(*n*_2_)) skyrmion bags. The color coding indicates the magnetization orientation as defined in the colorbar: white represents an out-of-plane upward magnetization, while black represents an out-of-plane downward magnetization.

In a two-dimensional context, multiple-*Q* magnetic solitons have been envisioned as skyrmion bags or high-order skyrmions^17,18,24–27^. We introduce a notation *S*(*n*) to represent skyrmion bags having *n* interior skyrmions with *Q* = 1 and a total degree of *Q* = *n* – 1. This definition can also be expanded to more complex structures with *S*(*n*_2_) skyrmion bags inside a *S*(*n*_1_) skyrmion bag, called nested *S*(*n*_1_, *S*(*n*_2_)) skyrmion bags with *Q* = *n*_1_ – *n*_2_, as shown in Fig. 1. Advancing into three-dimensional topological magnetism, both theory and experiments confirm the existence of skyrmion bundles^19,21^, composed of high-order skyrmions at near-surface layers and skyrmion bags at interior layers. The exterior spiral boundary of these three-dimensional skyrmion bundles is topologically akin to magnetic Hopfions^21,28^.

The realization of multiple-*Q* magnetic solitons poses significant challenges due to the inherent contradictions in the magnetic field that arise during the stabilization of internal skyrmion clusters and boundary spirals. Previous studies have addressed these challenges by employing a two-step process: first, creating internal skyrmion clusters at a negative field, and then reversing the magnetic field to form boundary spirals^19,21,28^. This two-step approach has proven effective in generating diverse multiple-*Q* magnetic solitons^19,21,28^. Although simulations suggest the creation of multiple-*Q* solitons using electrical methods^29^, experimental confirmation remains elusive. The electrical creation of these solitons represents a crucial step towards the practical application of multiple-*Q* topological spintronics.

In this study, we experimentally demonstrate the electrical creation of skyrmion bags exhibiting multiple *Q* values and morphologies in FeGe lamella at zero magnetic fields. This achievement leverages the two-fold degeneracy of skyrmions at zero magnetic fields, where both skyrmions with *Q* = − 1 and 1 are stable. This degeneracy provides a natural platform for assembling skyrmion bags. By utilizing current-induced spin-transfer torque^30^, we manipulate skyrmions with *Q* = 1 that are encircled by skyrmions with *Q* = − 1 (and vice versa), resulting in the formation of diverse nested skyrmion bags embedded in a skyrmion-lattice background magnetization^26^. Furthermore, we report the observation of the current-induced collapse of skyrmion tubes within these nested skyrmion bags. This collapse process leads to the evidence of the emerging topological defects, such as monopoles and bobbers^7,31^, which are controlled by the applied currents. These findings not only demonstrate the feasibility of electrically creating multiple-*Q* magnetic solitons but also open new avenues for exploring and manipulating topological defects in magnetic systems.

## Results

### Electrical creation of magnetic skyrmion bags

A 100-nm-thick FeGe lamella^32^, equipped with two electrodes, was fabricated for in-situ Lorentz transmission electron microscopy (TEM) imaging^33,34^, as shown in Fig. 1a. In the *B*_20_ FeGe magnets, the skyrmion size is about 70 nm^19,32^. Herein, we demonstrate the creation of magnetic skyrmion bags utilizing pulsed currents. Notably, the outermost magnetizations of both *S*(*n*) and *S*(*n*_1_, *S*(*n*_2_)) bags exhibit an out-of-plane up orientation, as shown in Fig. 1c. Conversely, when the outermost magnetizations of skyrmion bags exhibit an out-of-plane down orientation, we refer to them as –*S*(*n*) bags, with a topological charge of *Q* = 1 – *n*, and –*S*(*n*_1_, *S*(*n*_2_)) bags, with a topological charge of *Q* = *n*_2_ – *n*_1_.

**Fig. 2:**
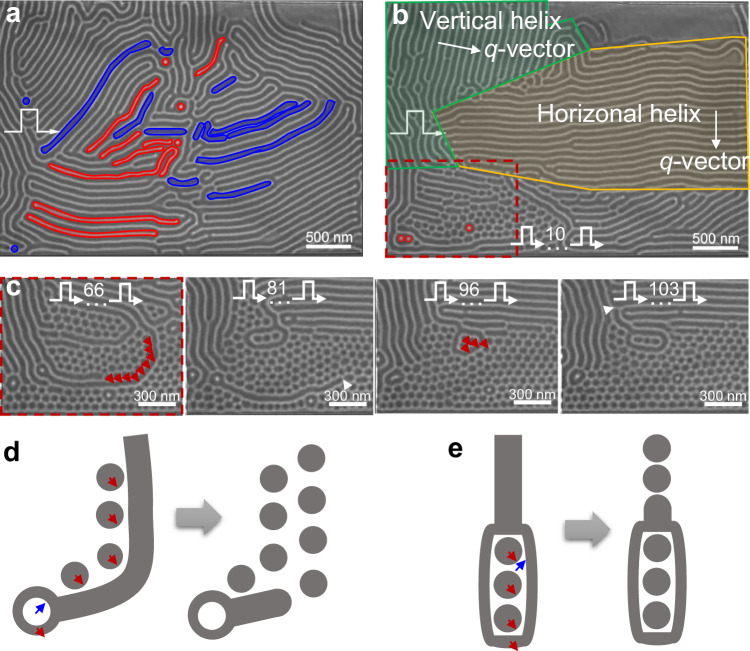
Current-induced creation of skyrmion bags at zero magnetic fields. **a** Initial helix domains after applying a single high pulsed current. Blue and red boxes represent the closure helix domains topologically equivalent to skyrmions with *Q* = 1 and −1, respectively. **b** Magnetic configuration after applying 10 pulsed currents on the initial helix domains. **c** Snapshots of creating skyrmion bags during the application of a series of pulsed currents. **d** Schematic formation of *S*(*n*) bags because of the split of helix during the rush of moving individual skyrmions. **e** Schematic formation of *S*(*S*(*n*)) bags because of the split of helix during the rush of internal individual skyrmions. The arrows in **d** and **e** mark the moving orientations of individual skyrmions.

Figure 2 presents a comprehensive overview of the current-driven dynamic transformations, commencing from the initial helix state. The current pulses have a duration of 70 ns. We define a positive current pulse as one in which the current flows from the left to the right side of the device, and a negative current pulse as one in which the current flows from the right to the left side. Notably, when the current density *j* remains below 3.5 × 10^10 ^A/m^2^, the helix domains exhibit no discernible dynamic responses (Supplementary Fig. 1a). However, as the current density rises within the range of 3.5–5.0 × 10^10 ^A/m^2^, we observe a transformation from the helix state to a skyrmion lattice, as depicted in Fig. 2b and c, Supplementary Fig. 1b, and Supplementary Movies 1 and 2. Nevertheless, it is crucial to note that once the current density surpasses 5.5 × 10^10 ^A/m^2^, the current-induced helix exhibits chaotic dynamics, primarily attributed to the significant Joule thermal heating effect (Supplementary Fig. 1c and Movie 3). Thus, there present a optimal current density *j*_opt_ for current-induced creation of skyrmions and skyrmion bags (Supplementary Fig. 2).

In chiral magnets, the competition between exchange and Dzyaloshinskii-Moriya interaction (DMI) forces the magnetizations to twist periodically on a plane either perpendicularly (helix) or with a canting angle (cone) along a specific direction, defined as *q*-vector^35,36^. For the initial helix (Fig. 2a), there are no preferred *q*-vectors. Once current is applied, the spin-transfer torque forces the formation of a horizontal helix with a *q*-vector perpendicular to the current orientation, as experimentally identified (Fig. 2b). However, we can always observe a perpendicular helix whose top ends are pinned from the geometrical edges. A prior study has conclusively established the creation of skyrmions from the perpendicular helix triggered by spin-transfer torque, which can be attributed to the force analysis of the instability of helix ends^37^. Skyrmions are first split from the perpendicular helix and then move to the bottom side because of skyrmion Hall effects^38,39^, resulting in the accumulation of skyrmions. Specifically, for positive currents, we consistently observe the formation of a skyrmion lattice with a negative topological charge, *Q* (Supplementary Fig. 3). Conversely, reversing the current polarity results in the emergence of a skyrmion lattice with a positive *Q* (Supplementary Fig. 3).

It is noteworthy that, due to the two-fold degeneracy of skyrmions with *Q* = 1 and *Q* = − 1 in the absence of an external field, a few individual skyrmions or closed helix domains with positive and negative *Q* can both persist (Fig. 2a). As positive pulsed currents are successively applied, these individual skyrmions with positive *Q* have chances to be embedded into a larger closed helix with negative *Q* (Fig. 2c). Because of skyrmion Hall effects driven by current, the long helix could undergo the integral crash forces of external skyrmion clusters moving to the bottom ends, contributing to the formation of individual *S*(*n*) bags (Fig. 2d). Especially, there has a chance to form −*S*(*n*) skyrmion bags embedded into a large closed helix (Fig. 2c). The long helix undergo tension force from the internal bag and can be split to form individual nested skyrmion bags (Fig. 2e). After approximately 120 pulsed currents, the number of skyrmions reaches a stable equilibrium, and no further skyrmion bags are created (Supplementary Movies 1–3). Under continuous current stimulation, skyrmions are generated to form a lattice with a uniform topological charge, *e.g*., *Q* = − 1. In contrast, skyrmion bags consist of internal skyrmions with *Q* = 1, which predominantly originate from the initial disordered state (Fig. 2a). As a result, skyrmion bags appear as isolated objects surrounded by a skyrmion lattice, and their number is inherently limited by the availability of oppositely charged seeds.

The temperature rise is closely linked to the current density due to the Joule thermal heating effect^40^. As the current density increases, so does the temperature. Once the temperature increase reaches approximately 250 K, the helix domains become the only stable state at zero field^32^. Therefore, by applying a single high-density current pulse, we can effectively reset the initial helical domains (Supplementary Fig. 4 and Supplementary Movie 4), enabling us to repeat the dynamic process depicted in Fig. 2 and Supplementary Movie 1. In individual experiments, we adopt a standardized procedure. First, we apply a single pulsed current with a density of 5.6 × 10^10 ^A/m^2^ to restore the initial helix domains. Subsequently, we apply approximately 150 (low) current pulses to induce the generation of skyrmions and skyrmion bags.

**Fig. 3:**
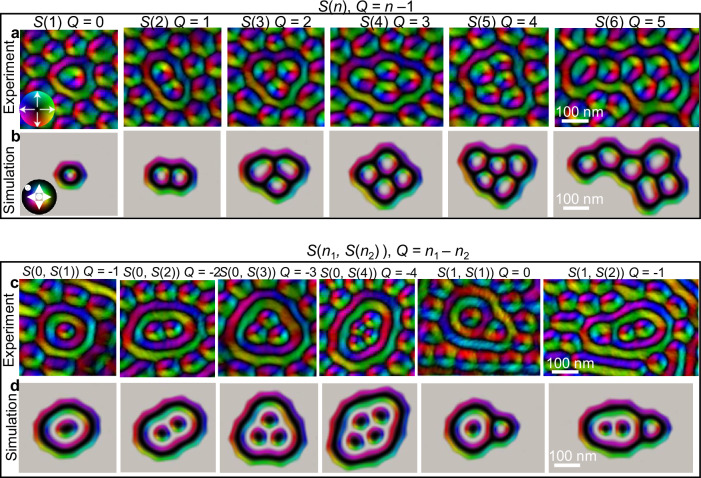
Representative skyrmion bags captured during the cycle of current-induced helix-to-skyrmion transformations. **a, b** Experimental observations (**a**) and corresponding simulations (**b**) of traditional *S*(*n*) skyrmion bags, exhibiting a topological charge of *Q* = *n* − 1. **c, d** Experimental observations (**c**) and corresponding simulations (**d**) of nested *S*(*n*_1_, *S*(*n*_2_)) skyrmion bags with *Q* = *n*_1_ − *n*_2_. The experimental magnetizations are retrieved through a typical transport of intensity equation (TIE) analysis, while the simulated magnetizations are extracted from the middle layers of the skyrmion bags. The colors in (**a**) and (**c**) represent experimental in-plane magnetization according to the colorwheel in (**a**). The colors in (**b**) and (**d**) represent simulated magnetizations according to the colorwheel in (**b**).

In our multiple individual experiments, we successfully generated skyrmion bags exhibiting various topological charges, as shown in Fig. 3a. For skyrmion bundles characterized by a depth-modulated spin twisting^19,21^, the average in-plane magnetizations of the boundary spirals are significantly weaker compared to those of the interior skyrmions. However, for skyrmion bags, the retrieved average in-plane magnetizations of the boundary spirals are comparable to those of the interior skyrmions. This notable difference can be attributed to the varying background magnetizations. In our present experiments, the skyrmion bag is stabilized within a skyrmion lattice. Consequently, the background magnetization can be approximated as a ferromagnet, leading to negligible spin twists along the depth orientation for skyrmion bags. Our simulations further confirm this observation (Fig. 3b), revealing that the spin textures across all layers maintain their skyrmion bag characteristics (Supplementary Fig. 5).

We successfully generated not only *S*(*n*) skyrmion bags but also intricate nested *S*(*n*_1_, *S*(n_2_)) skyrmion bags by utilizing pulsed currents, as shown in Fig. 3c and Supplementary Fig. 6. The simulation well reproduced the intricate configurations (Fig. 3d). The formation process of these nested skyrmion bags can be outlined in two distinct steps. Initially, an −*S*(*n*_2_) skyrmion bag surrounded by a helix domain is created (Fig. 1c). Subsequently, the helix domain undergoes a breakage process, resulting in the emergence of an *S*(*n*_1_) skyrmion bag that encircles the inner −*S*(*n*_2_) skyrmion bag, thus giving rise to the nested *S*(*n*_1_, *S*(n_2_)) skyrmion bags configuration.

Current-induced effects also include Joule thermal heating and the Oersted field. By reversing the current orientation, the topological charges of current-induced skyrmions and skyrmion bags are reversed (Supplementary Fig. 7), ruling out Joule heating as the main origin, since it does not depend on current direction. Our multiphysic field simulation shows a maximum temperature rise of about 14 K for a 70-ns current pulse with a density of 4 × 10^10 ^A m^−2^ (Supplementary Fig. 8). This moderate temperature rise may assist the splitting of skyrmions and skyrmion bags from the ends of a long perpendicular helix, but the topological sign is determined solely by STT. To assess the possible influence of the Oersted field, we performed multiphysics field simulations of the field distribution at the applied current density (Supplementary Fig. 9). The Oersted field is predominantly in-plane, with a maximum magnitude of approximately 2.86 mT, and its orientation differs between the top and bottom surfaces. In our FeGe devices, skyrmion creation typically requires an out-of-plane field on the order of 100 mT. The Oersted field is therefore (i) purely in-plane, (ii) an order of magnitude smaller, and (iii) spatially varying, making it incapable of nucleating skyrmions. Furthermore, symmetry and force analysis yield four distinct STT‑driven dynamical behaviors depending on the pinning ends of the vertical helix and the current orientation (Supplementary Fig. 10). Our experiments reproduce all four predicted schemes (Supplementary Fig. 11). Consequently, we conclude that STT is the primary effect determining the topological sign of current‑induced skyrmion bags and skyrmions, while Joule heating plays at most an auxiliary role. Since our devices are based on FeGe without adjacent heavy-metal layers, spin–orbit torques are absent, and the current-induced torque is dominated by the in-plane Zhang–Li STT^30^. The creation mechanism of skyrmion bags relies on the coexistence of skyrmions with opposite topological charges at zero magnetic field, and the STT-driven instability of helix ends—both of which are rooted in topology and dynamics rather than in specific material parameters. Therefore, our experimental findings can, in principle, be generalised to other skyrmion-hosting systems, including room-temperature chiral magnets and multilayers with interfacial DMI^14^.

**Fig. 4:**
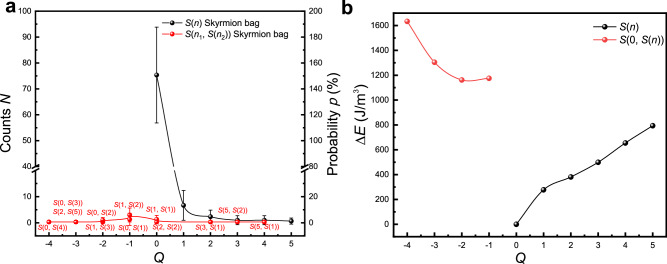
Probability for electrical creation of skyrmion bags. **a** Counts of magnetic skyrmion bags, *N*, observed over 50 independent cycles, during the transition from a disordered helical state to a skyrmion lattice. The generation probability is calculated as *p* = *N*/50 × 100%, representing the likelihood of skyrmion bag formation per current cycle. Each current cycle consists of a single high-current-density pulse followed by 150 low-current-density pulses. The statistical data were obtained from three devices with similar dimensions: length ~4 μm, width ~3 μm, and thickness ~100 nm. Error bars represent the standard deviation across 3 devices. **b** Simulated total energy difference $\Delta$*E* between skyrmion bags and the *S*(1) bag embedded in the skyrmion lattice.

We define a current cycle as the process of transforming helical domains primarily into a skyrmion lattice, driven by the application of current (Supplementary Movie 1 for reference). Figure 4a presents the total counts of magnetic skyrmion bags obtained from 50 individual current cycles. Each current cycle consists of a single high-current pulse with a density of 5.5 $\times$ 10^10 ^A/m², followed by 150 low-current pulses with a density of 4.5 $\times$ 10^10^ A/m². Initially, a few skyrmions with topological charges *Q* = 1 and *Q* = − 1 are present. Upon application of positive current pulses, *Q* = − 1 skyrmions are generated, whereas *Q* = 1 skyrmions either gradually annihilate or evolve into skyrmion bags (Supplementary Fig. 12). Our findings indicate that *S*(1) bags, also known as skyrmionium or 2π-vortex, have the highest likelihood of being generated. Notably, the generation possibility of *S*(*n*) skyrmion bags decreases as *n* increases. This trend can be attributed to the instability of high-*Q* skyrmion bags during current-driven dynamics, as demonstrated in Supplementary Movies 5 and 6. These Movies reveal transitions from *S*(3) with *Q* = 2 to *S*(2) with *Q* = 1 and *S*(6) with *Q* = 5 to *S*(5) with *Q* = 4, indicating a reduction in topological charge during the application of current stimuli. Due to the intricate two-step creation process involved in forming nested *S*(*n*_1_, *S*(*n*_2_)) skyrmion bags, only a few such bags can be observed. The likelihood of their creation aligns with the trend in the total free energy *E* of skyrmion bags, as illustrated in Fig. 4b. Our simulations reveal that *S*(1) bags possess the lowest energy, with the total energy increasing as *Q* increases. Furthermore, nested skyrmion bags generally exhibit relatively high energies. The skyrmion bags stabilize as high-energy metastable phases, originating from initial skyrmion seeds with different signs of *Q* contrast to that of the skyrmion lattice. To keep the outer magnetization consistency, the initial skyrmions with *Q* = 1 must be encircled by a skyrmion with *Q* = − 1, resulting in the formation of skyrmion bags.

The reproducibility of current-induced skyrmion bag creation was tested in multiple microdevices with thicknesses ranging from 80 to 150 nm. Consistent current-induced dynamics were observed across all devices (Supplementary Figs. 13–16). Owing to their high-energy metastable nature, thermal fluctuations promote the transformation from skyrmion bags to isolated skyrmions. Accordingly, our experiments show that the probability of skyrmion bag formation per current cycle decreases with increasing temperature (Supplementary Fig. 17b). In contrast, the probability of skyrmion bag formation per current cycle decreases with increasing pulse width (Supplementary Fig. 17a). Under longer current stimuli, there is a higher probability that skyrmion bags will transform into isolated skyrmions, driven by thermal fluctuations and the continuous application of spin-transfer torque. To assess the stability of skyrmion bags, we monitored the magnetic texture under constant conditions (zero applied current, 95 K, and zero external magnetic field) for over 13 h (Supplementary Fig. 18). Repeated imaging reveals no detectable changes in the morphology, size, or position of the skyrmion bags, demonstrating their long-term stability.

### Current-induced topological transformations assisted by the emergence of topological defects

**Fig. 5:**
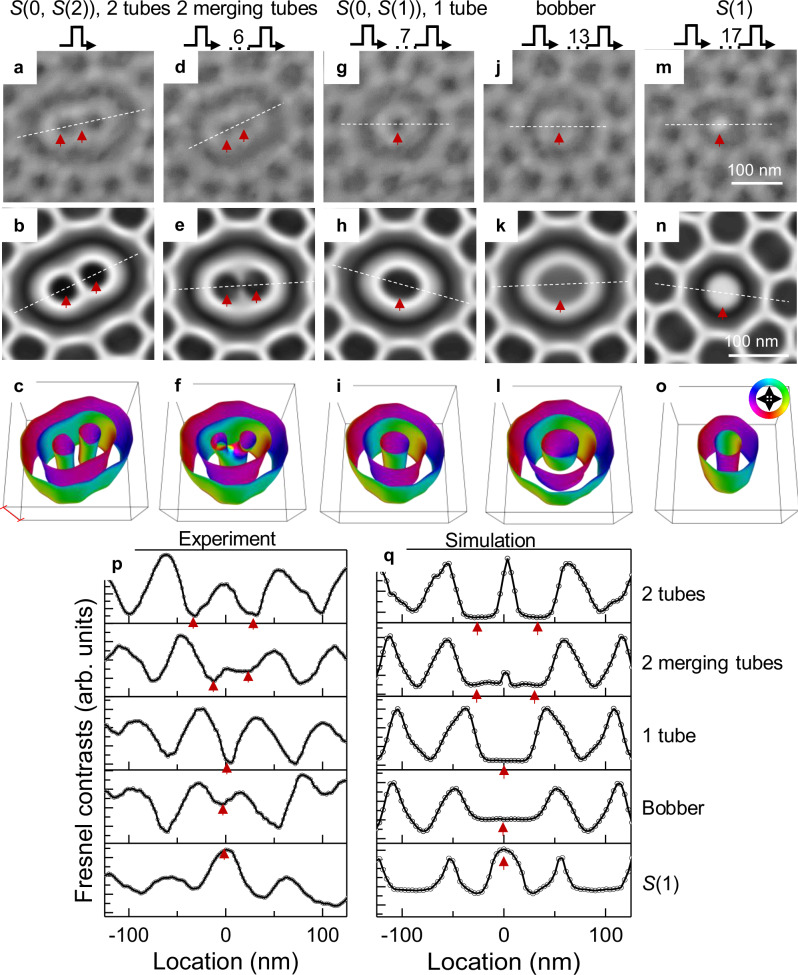
Current-induced collapse process of interior skyrmion tubes of a nested *S*(0, *S*(2)) skyrmion bag. Experimental Fresnel contrasts, simulated Fresnel contrasts, and simulated 3D magnetization isosurfaces during the collapse process. **a**–**c** An initial nested *S*(0, *S*(2)) skyrmion bag. **d**–**f** A nested skyrmion bag containing two merging skyrmion tubes was obtained after applying 6 current pulses. **g**–**i** A nested *S*(0, *S*(1)) skyrmion bag containing one tube obtained after applying 7 current pulses. **j**–**l** a nested skyrmion bag containing 1 bobber obtained after applying 13 current pulses. **m**–**o** *S*(1) skyrmion bag obtained after applying 17 current pulses. The iso-surface corresponds to zero out-of-plane magnetization. The color scheme in the isosurface illustrates the in-plane magnetization, based on the colorwheel. Defocused distance of Fresnel contrasts, −500 μm. **p, q** Experimental (**p**) and simulated (**q**) Fresnel contrasts along the corresponding white dashed lines.

We have shown the metastable nature of high-*Q* or nested bags, which possess higher energies compared to *S*(1) bags. Given this, it is reasonable to expect topological transitions within skyrmion bags during the application of current stimuli. Here, we further explore the dynamics of magnetic skyrmion bags driven by current, as shown in Fig. 5. During the application of pulsed currents, we observe distinct transformations from *S*(*n*) to *S*(*n* − 1) bags (Supplementary Movies 5 and 6). These transformations are directly linked to the collapse of skyrmion tubes within the bags. The topological transformations from *S*(*n*) to *S*(*n* − 1) bags are understood by the instability of skyrmion bags due to the current-induced Joule thermal heating. For longer current density and longer current pulse width, the Joule thermal heating effect becomes more remarkable, causing the easy transformations (Supplementary Figs. 19 and 20). If the current orientation is reversed, based on the symmetry analysis and selective polarity rule (Supplementary Fig. 10), the initial skyrmion bags annihilate during the background magnetization transitions between *Q* = − 1 skyrmion lattice and *Q* = 1 skyrmion lattice (Supplementary Fig. 7).

The collapse process of skyrmion tubes can be categorized into two distinct styles^41^. Firstly, there is the merger of two skyrmion tubes, facilitated by topological monopoles in Y-shaped skyrmion tubes. Secondly, a skyrmion tube is broken from the interior, assisted by magnetic bobbers. Although these topological defects emerge during field-driven annihilations of skyrmion tubes^31,42,43^, experimental observation using electrical methods has remained elusive. Here, we present compelling evidence of these typical collapse processes in the context of current-induced topological quantized annihilation of nested skyrmion bags. Starting with an *S*(0, *S*(2)) nested skyrmion bag as the initial state (Fig. 5a–c), we observe a transition to an *S*(0, *S*(1)) nested skyrmion bag after the application of a series of current pulses (Fig. 5d, e). This topological transformation is achieved through the merging of two internal skyrmion tubes, which exhibit a narrow gap between them and contribute to a reduced Fresnel contrast (Fig. 5e, f). This phenomenon is consistently observed in our experiments (Fig. 5d). With further application of pulsed currents, we observe a subsequent transformation from the *S*(0, *S*(1)) state to an *S*(1) bag (Fig. 5g-o). This transition is associated with the collapse of a skyrmion tube (Fig. 5j–l). Notably, in our experiments, we identify an intermediate state characterized by a very weak, dotted-like Fresnel contrast (Fig. 5j). This observation can be attributed to the emergence of a magnetic bobber during the collapse process (Fig. 5k and l). The abrupt changes in Fresnel contrast during the collapse processes of skyrmion tubes in experiments (Fig. 5p) are highly consistent with simulations (Fig. 5q), providing experimental proof for the stabilization of complex 3D topological spin textures.

The emergent intermediate collapsed state of skyrmion tubes driven by current can be widely achieved for nested skyrmion bags (Supplementary Figs. 21 and 22). Typically, we can obtain various styles of internal contrasts for internal topological configurations of nested skyrmion bags (Supplementary Fig. 22), suggesting the stability of bobbers with various penetration depths. Our findings provide experimental evidence for the collapse mechanisms of skyrmion tubes within skyrmion bags and offer insights into the dynamic behavior of these complex magnetic structures under the influence of current stimuli. The distinction between depth-uniform skyrmion tubes and depth-modulated 3D solitons (such as magnetic bobbers or skyrmion bundles) based on Lorentz TEM contrast differences has been established in previous studies^7,16,19,34^. Following this principle, our observed contrast features are consistent with the presence of 3D topological structures, but we refer to them as experimental signatures rather than definitive proof. For direct and unambiguous reconstruction of the full 3D spin configuration, future work should employ tilt-series Lorentz TEM imaging over a wide angular range (e.g., –60° to +60°) combined with tomographic reconstruction^44–46^.

Previous studies have established emergent skyrmion lattice dynamics, including rotation driven by spin torques, thermal gradients, and field gradients^47–49^. Typically, such dynamics are inferred from shifting scattering peaks in reciprocal-space neutron scattering experiments^49^. Skyrmion bags—which can be regarded as topological defects within the lattice—offer a unique opportunity to directly image lattice dynamics in real space. In our observations, skyrmion bags undergo counter-clockwise rotation within the lattice, revealing the counter-clockwise rotation of the entire skyrmion lattice (Supplementary Fig. 23 and Movie 3). These findings suggest a pathway for device applications that exploit skyrmion lattice dynamics by detecting the motion of skyrmion bags (Supplementary Fig. 23d). The electrical detection of skyrmion bags with different topological charges *Q* can be achieved via the topological Hall effect, whose magnitude is proportional to the *Q* value within the detection region^50–52^. For more efficient and practical read-out, tunnel magnetoresistance effects—integrated into a magnetic tunnel junction geometry—can be used to distinguish different *Q* values of skyrmion bags (Supplementary Fig. 23d)^53–55^. This provides a pathway for all-electrical writing, manipulation, and reading of skyrmion-bag-based spintronic devices.

## Discussion

In summary, we have experimentally achieved the first instance of the current-induced creation of magnetic skyrmion bags without the application of an external magnetic field. The unique twofold degeneracy exhibited by skyrmions, coupled with the selective polarity arising from the end instability of the helix when a pulsed current is applied, underlies the electrical creation of these skyrmion bags. Our study further uncovers novel current-induced bag dynamics, encompassing topological quantized annihilations. We propose that the intricate collapsed processes involving skyrmion tubes within nested skyrmion bags could pave the way for the realization of monopoles and bobbers through electrical methods. The creation and topological transformation assisted by emerging topological defects, facilitated solely by currents in the absence of an external field, not only provide further insight into skyrmion physics but also open up new avenues for the development of multiple-*Q* spintronics.

The stochastic nature of skyrmion bag formation in our zero‑field electrical approach, while currently limiting deterministic operation, highlights the governing roles of local domain terminations and current direction. Achieving deterministic electrical creation of skyrmion bags will require further exploration, particularly through local manipulation techniques such as laser irradiation and lithographically defined pinning sites.

## Methods

### Fabrications of FeGe microdevice

A thin FeGe lamella with a thickness of ~80-150 nm for TEM magnetic imaging was fabricated by the lift-out method using a focused-ion beam and scanning electron microscopy dual beam system (Helios Nanolab 600i, FEI) in combination with a gas injection system and a micromanipulator (Omniprobe 200 + , Oxford). The results presented in the main text figures are obtained from the same device (Device #1), which has a length of 4 μm, a width of 3 μm, and a thickness of 100 nm.

### TEM measurements

Magnetic imaging was carried out using a TEM instrument (Talos F200X, FEI) operated at 200 kV at the Fresnel mode. The objective lens is switched off to provide a field-free condition. A single-tilt liquid-nitrogen specimen holder (Model 616.6 cryotransfer holder, Gatan) was used for varying temperature measurements. The pulsed current with a pulse duration of 20-160 ns and a frequency of 1 Hz was provided by a voltage source (AVR-E3-B-PN-AC22, Avtech Electrosystems). All experiments were performed at zero magnetic field.

### Numerical simulations

Micromagnetic simulations were performed using MuMax3^56^. The total free energy terms are written as: $\varepsilon={\int }_{{V}_{s}}\left\{{\varepsilon }_{{\mbox{ex}}}+{\varepsilon }_{{{\rm{DMI}}}}+{\varepsilon }_{{\mbox{dem}}}\right\}{{\rm{d}}}{{\boldsymbol{r}}}$. Here, exchange energy ${\varepsilon }_{{\mbox{ex}}}=A({\partial }_{x}{{{\bf{m}}}}^{2}+{\partial }_{y}{{{\bf{m}}}}^{2}+{\partial }_{z}{{{\bf{m}}}}^{2})$, DMI energy ${\varepsilon }_{{\mbox{DMI}}}={D}_{{{\rm{dmi}}}}{{\bf{m}}}\cdot [\nabla \times {{\bf{m}}}]$, and demagnetization energy ${\varepsilon }_{{\mbox{dem}}}=-\frac{1}{2}{M}_{{{\rm{s}}}}{{{\bf{B}}}}_{{{\rm{d}}}}{{\bf{m}}}$. Here ${{\bf{m}}}\equiv {{\bf{m}}}(x,y,z)$ is the normalized units continuous vector field that represents the magnetization ${{\bf{M}}}\equiv {M}_{{{\rm{s}}}}{{\bf{m}}}(x,y,z)$. *A, D*_dmi_, and *M*_s_ are the exchange interaction, DMI interaction, and saturation magnetization, respectively. **B**_d_ is the demagnetizing field. We set a typical value for saturation magnetization *M*_s_ = 384 kA m^−1^ for FeGe^57^. The exchange interaction *A*_ex_ = 3.25 pJ/m is determined from the fit to the field-dependence of magnetization evolution^57^. DMI interaction ${D}_{{{\rm{dmi}}}}=4{{\rm{\pi }}}A/{L}_{{{\rm{D}}}}=$ 0.5834 mJ m^−2^ is obtained from zero-field spin spiral period *L*_D_ = 70 nm^57^. We set the cell size as 2 × 2 × 2 nm^3^. We obtained the equilibrium spin configurations using the conjugate-gradient method.

## Supplementary information

Supplementary Information

Description of Additional Supplementary Files

Supplementary Movie 1

Supplementary Movie 2

Supplementary Movie 3

Supplementary Movie 4

Supplementary Movie 5

Supplementary Movie 6

Supplementary Movie 7

Supplementary Movie 8

Supplementary Movie 9

Transparent Peer Review file

## Data Availability

The data that support the findings of this study have been included in the main text and Supplementary Information. Raw LTEM data (images and movies) are available from the corresponding authors upon reasonable request.
