# Supplementary material for: Current-induced creation and dynamics of embedded magnetic skyrmion bags
Source: Nat Commun. 2026 Jun 11;17:7442. doi: 10.1038/s41467-026-74046-4 (PMC13408094; doi:10.1038/s41467-026-74046-4)
Supplement: Supplementary file 1 — Supplementary Information [file 41467_2026_74046_MOESM1_ESM.pdf]

Supplementary Information

Current-induced creation and dynamics of embedded magnetic skyrmion bags

Yaodong Wu[1], Jialiang Jiang[2, 3*], Lingyao Kong[2], Meng Shi[3], Shouguo Wang[4], Mingliang Tian[2, 3], Haifeng Du[3*], and Jin Tang[2, 3*]

[1]School of Physics and Materials Engineering, Hefei Normal University, Hefei 230601, China

[2]State Key Laboratory of Opto-Electronic Information Acquisition and Protection Technology, School of Physics, Anhui University, Hefei, 230601, China

[3]Anhui Provincial Key Laboratory of Low-Energy Quantum Materials and Devices, High Magnetic Field Laboratory, HFIPS, Chinese Academy of Sciences, Hefei 230031, China

[4]Anhui Provincial Key Laboratory of Magnetic Functional Materials and Devices, School of Materials Science and Engineering, Anhui University, Hefei 230601, China

*Corresponding author: jintang@ahu.edu.cn; duhf@hmfl.ac.cn; jjl2024@ahu.edu.cn

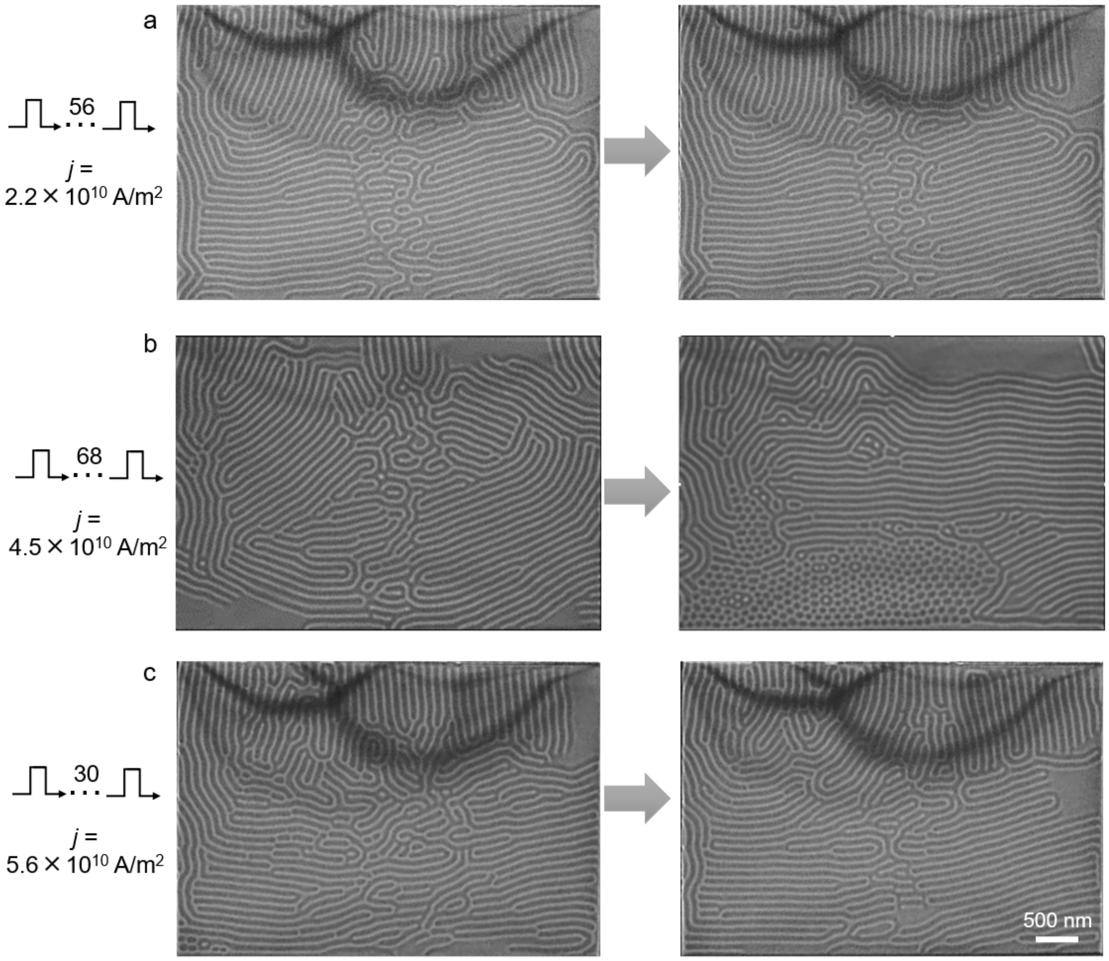

Supplementary Fig. 1 | Current-driven dynamics from the initial helix. a $j = 2.2 \times 10^{10}$ A/m^2. **b** $j = 4.5 \times 10^{10}$ A/m^2. **c** $j = 5.9 \times 10^{10}$ A/m^2. Pulse duration, 70 ns.

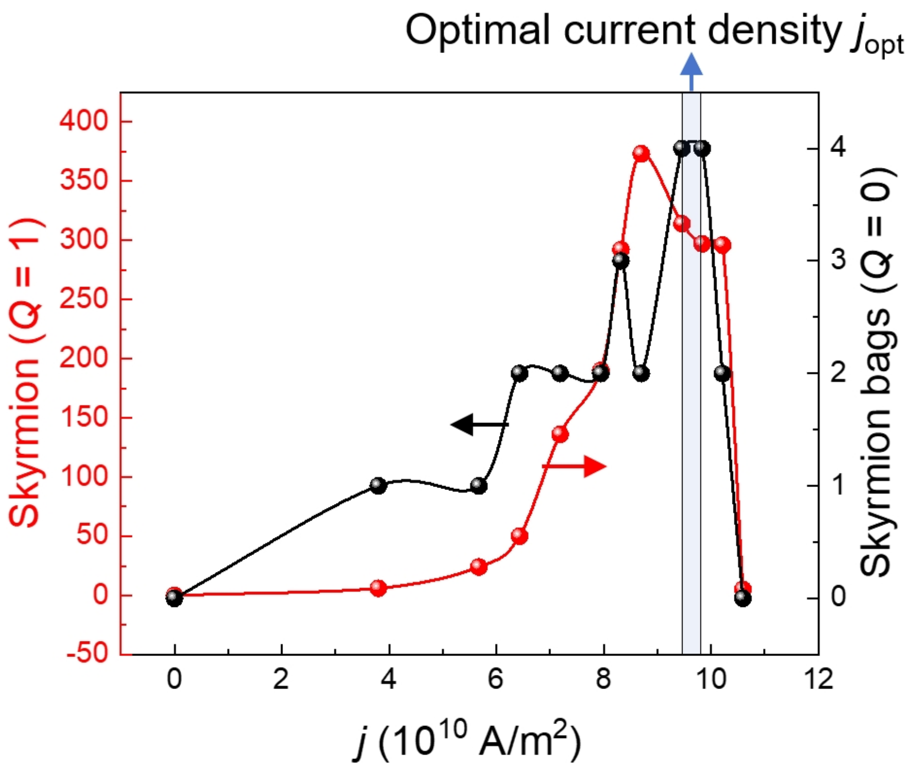

Supplementary Fig. 2 | The maximum skyrmion and Q = 0 skyrmion bag counts as a function of current density induced by current during each current cycle. Temperature, 100 K. Pulse duration, 20 ns.

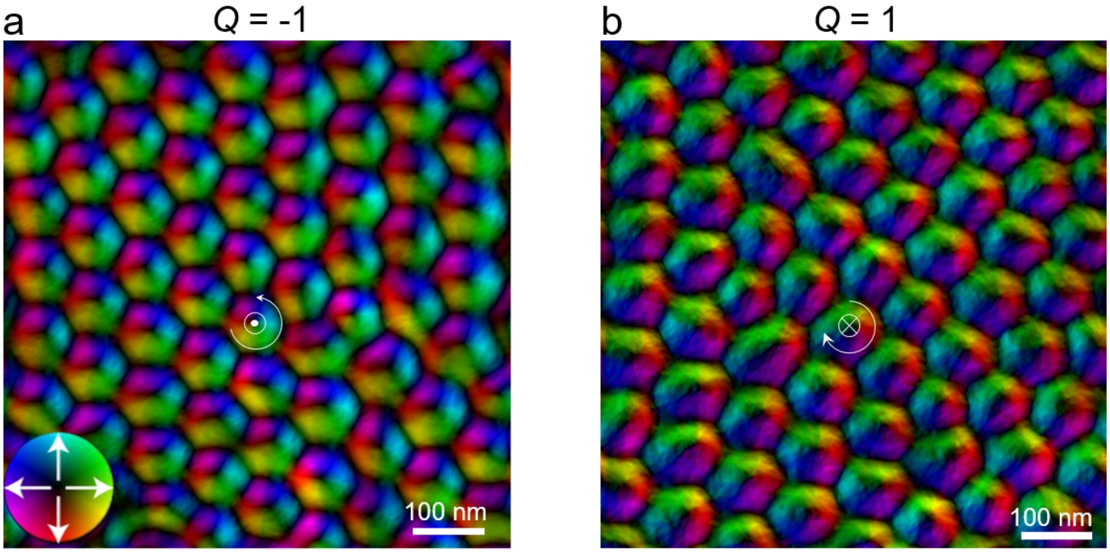

Supplementary Fig. 3 | Zero-field magnetic skyrmion lattices. a, b In-plane magnetization mapping of skyrmions with $Q = 1$ (a) and -1 (b) retrieved from TIE.

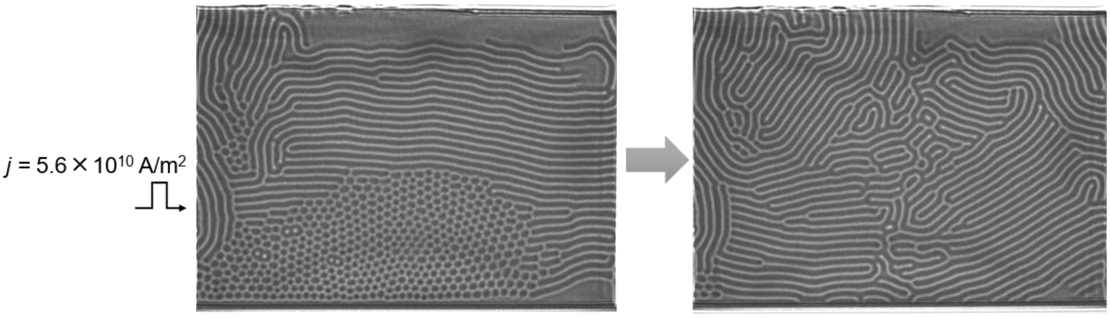

Supplementary Fig. 4 | Initial helix obtained by applying a single high current with a density of 5.6 × 10^{10} A/m^2.

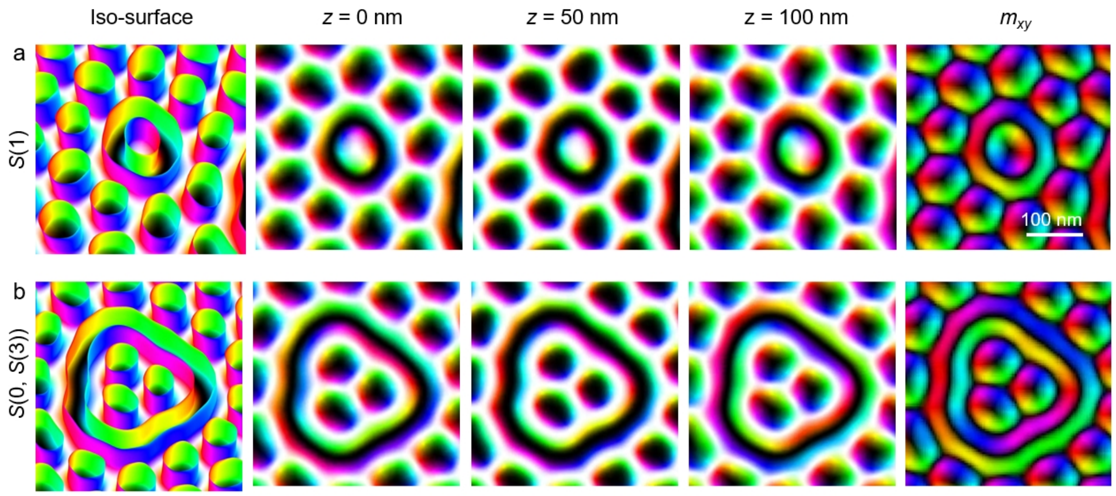

Supplementary Fig. 5 | 3D configuration of skyrmion bags. a, b 3D iso-surfaces for $m_z = 0$, magnetization at the top layer $z = 0$ nm, magnetization at the middle layer $z = 50$ nm, magnetization at the bottom layer, and in-plane average magnetization for the *S*(1) (a) and *S*(0, *S*(3)) (b) bags.

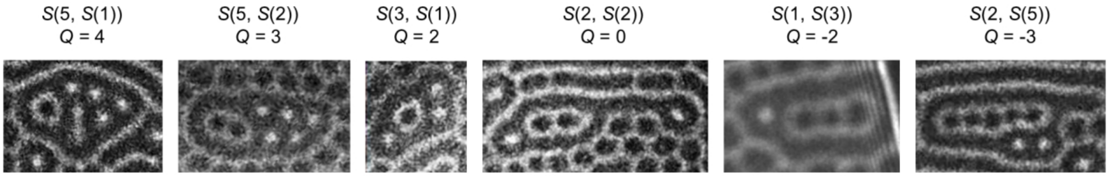

Supplementary Fig. 6 | Experimental observations of nested magnetic skyrmion bags at a zero magnetic field. De-focused Fresnel contrasts of *S*(5, *S*(1)), *S*(1, *S*(1)), *S*(5, *S*(2)), *S*(3, *S*(1)), *S*(2, *S*(2)), *S*(1, *S*(3)), and *S*(2, *S*(5)) nested skyrmion bags. Defocused distance, −500 µm.

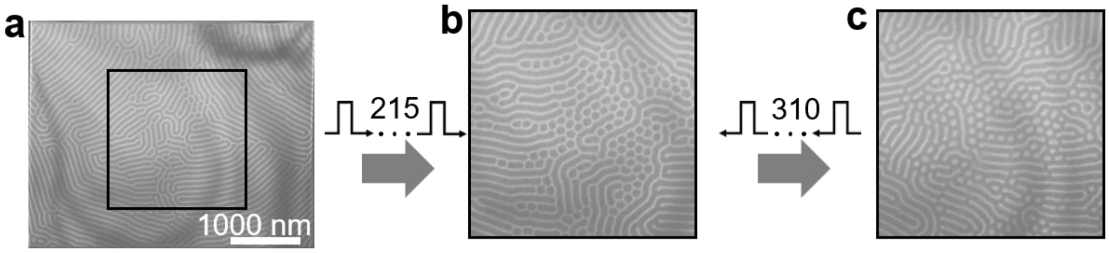

Supplementary Fig. 7 | Skyrmion polarity reversal by reversing the current orientation. **a** Initial domain states. **b** Current-induced skyrmions and skyrmion bags after applying 215 current pulses with a density of $+8.4 \times 10^{10}$ A m^{-2}. **c** Subsequent state after applying 310 current pulses with a density of -8.4×10^{10} A m^{-2}. The Fresnel images in (b) and (c) correspond to the region indicated by the black box in (a). Defocus distance: −500 μm.

Supplementary Table 1 | Parameters for Multiphysics field simulations.

Name	Parameter	Value	Unit	Notes
**FeGe**	Density (ρ_m)	8220	kg m^{-3}	Ref. [1]
	Heat capacity (C_p)	363	J kg^{-1} K^{-1}	Ref. [2]
	Thermal conductivity (k)	3.39	W m^{-1} K^{-1}	Ref. [2]
	Resistivity (ρ)	$(1+0.00948(T-100)) \times 80.87$	μΩ cm	Ref. [2]
	Heat transfer coefficient (h)	5×10^6	W m^{-2} K^{-1}	Ref. [2]
**I-Pt**	Density (ρ_m)	21450	kg m^{-3}	Ref. [3]
	Heat capacity (C_p)	132	J kg^{-1} K^{-1}	Ref. [3]
	Thermal conductivity (k)	71.6	W m^{-1} K^{-1}	Ref. [3]
	Resistivity (ρ)	$(1+0.00385(T-100)) \times 2.71$	μΩ cm	Ref. [4]
	Heat transfer coefficient (h)	5×10^6	W m^{-2} K^{-1}	Ref. [2]

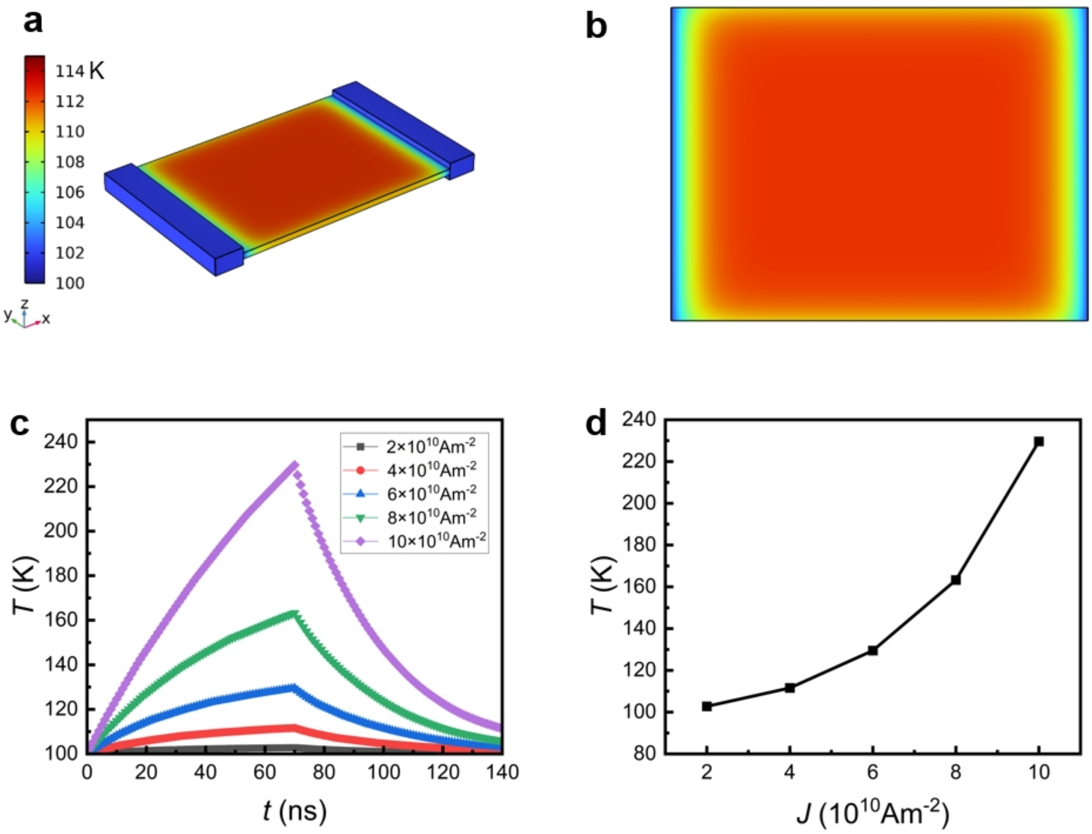

Supplementary Fig. 8 | Multiphysics field simulation of current-induced Joule thermal heating. a Simulated temperature distribution after a 70-ns current pulse with a current density of 4×10^{10} A m^{-2}. **b** Enlarged view of the temperature distribution in the device area. **c** Temperature at the center of the device as a function of time during the pulse for different current densities. **d** Maximum temperature rise versus current density for 70-ns pulses.

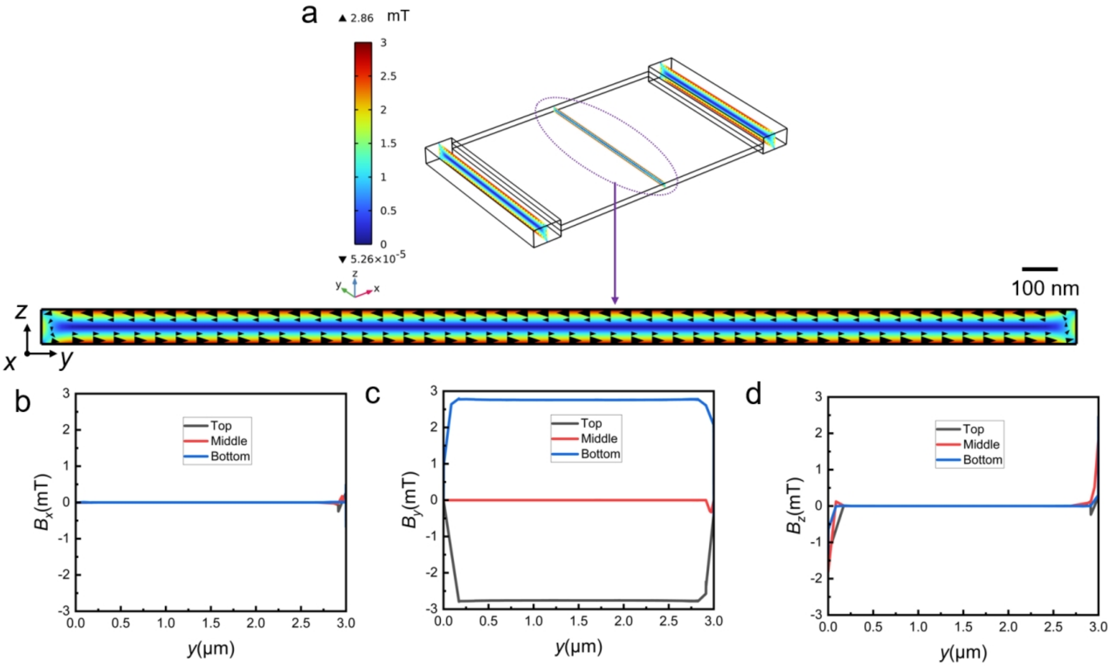

Supplementary Fig. 9 | Simulated Oersted field distribution of a 100-nm-thick FeGe microdevice at a current density of 4.5×10^{10} A·m^{-2} applied along the *x* axis. a The Oersted field distribution in the middle layer. The color represents the magnitude of the magnetic flux density. **b-d** Dependence of the Oersted field components on position *y* at the middle layer: B_x (b), B_y (c), and B_z (d).

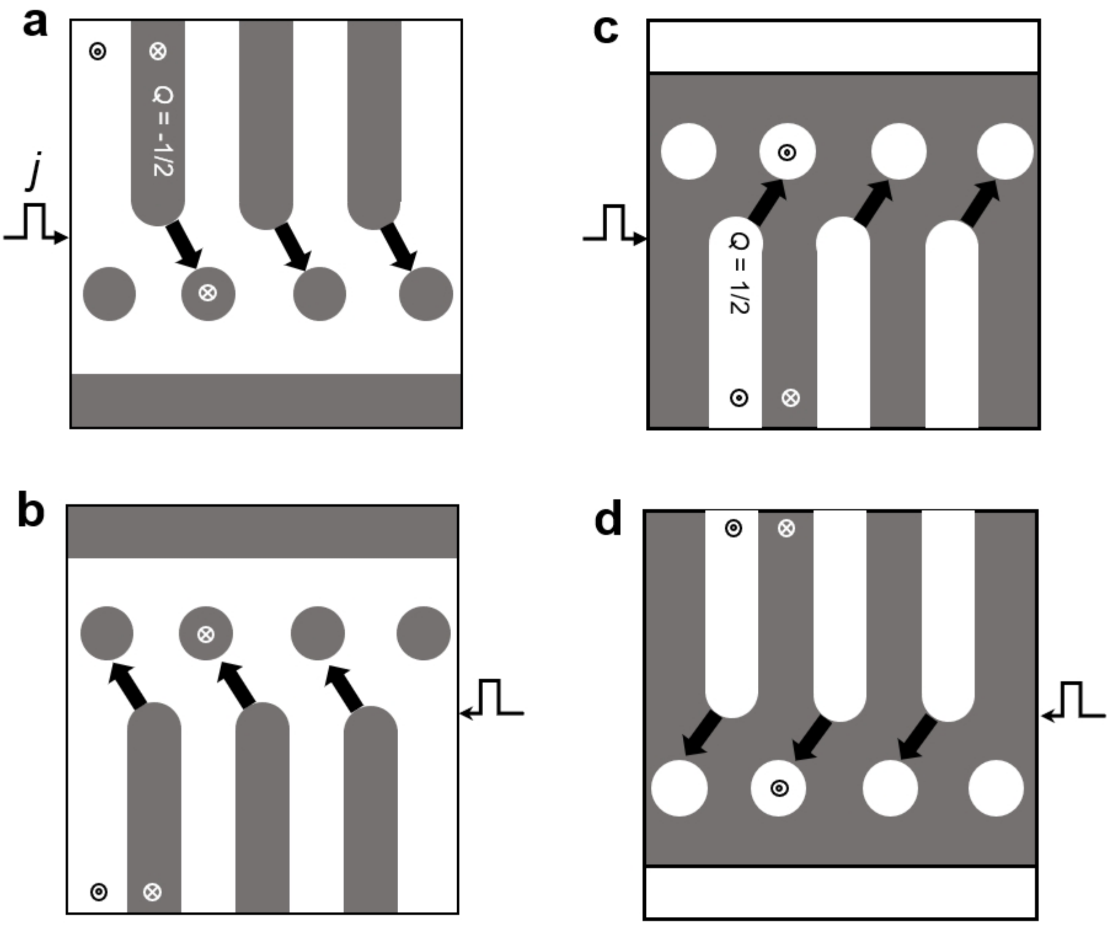

Supplementary Fig. 10 | Schematic skyrmion generation rule based on force and symmetry analysis. Four cases are considered depending on the helix pinning ends and current orientation. Black and white represent out-of-plane magnetization down and up, respectively. Black arrows indicate the motion direction of helix ends, equivalent to merons driven by STT. **a** vertical helix pinned at the top ends, positive current; **b** pinned at the bottom ends, positive current; **c** pinned at the bottom ends, negative current; **d** pinned at the top ends, negative current. Starting from (a), other cases in (b), (c), and (d) can be obtained by applying time-reversal and/or combined rotation of spin and space by 180 degrees to (a).

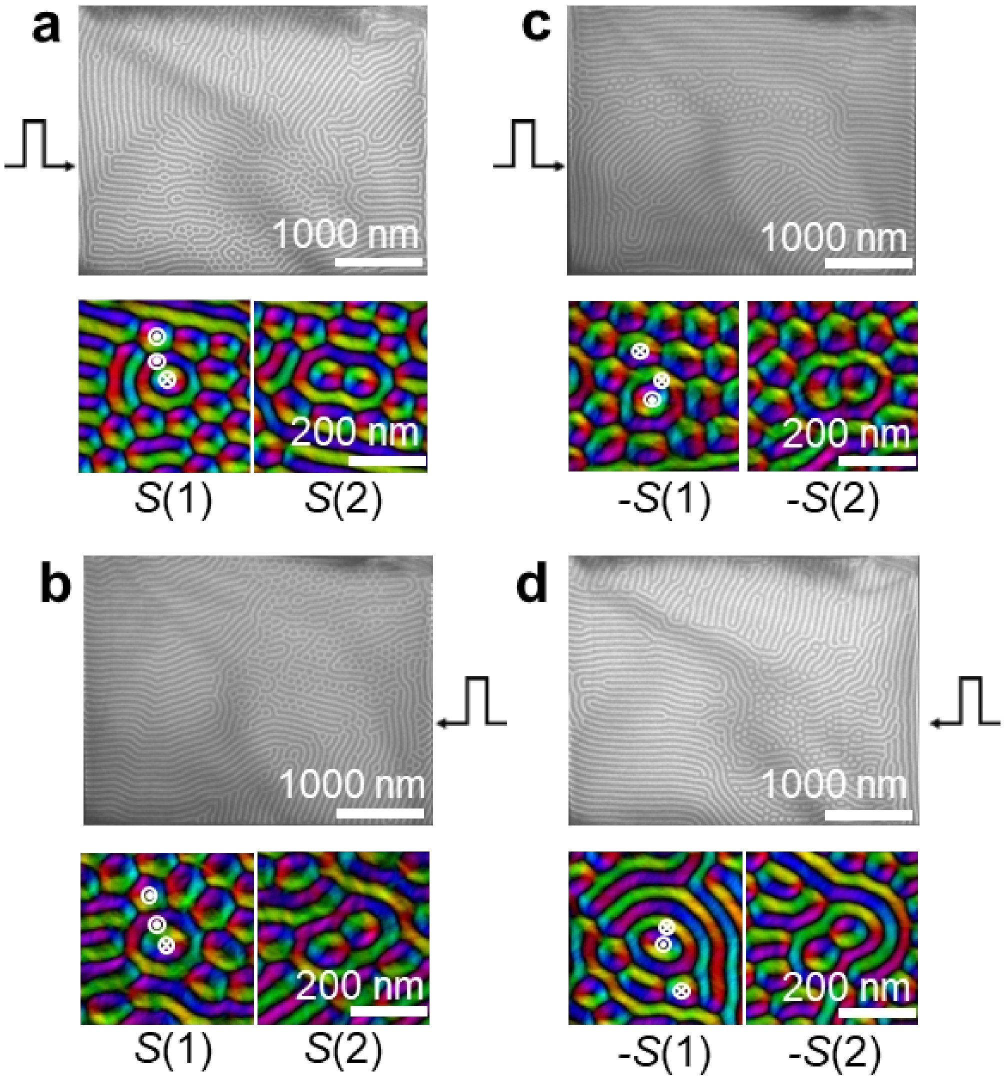

Supplementary Fig. 11 | Experimental demonstration of the skyrmion generation rule based on force and symmetry analysis. Fresnel images were taken at a defocus distance of −500 μm, revealing the pinning ends of the vertical helix. Representative in-plane magnetic configurations of skyrmion bags and skyrmions are shown for four cases, depending on the pinning location and current polarity: **a** vertical helix pinned at the top ends, positive current; **b** pinned at the bottom ends, positive current; **c** pinned at the bottom ends, negative current; **d** pinned at the top ends, negative current.

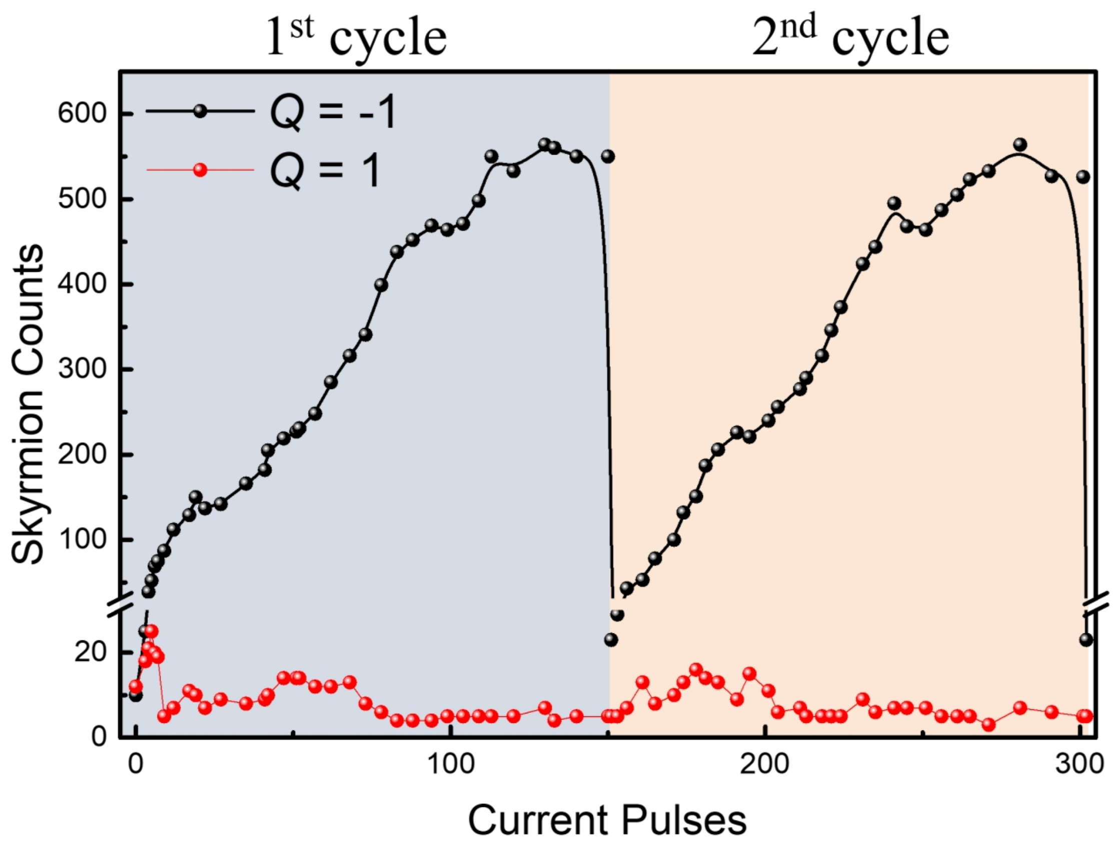

Supplementary Fig. 12 | Count of Q = 1 and −1 skyrmions during each current cycle. Each current cycle consists of a single high-current pulse with a density of 5.5×10^{10} A/m², followed by 150 low-current pulses with a density of 4.5×10^{10} A/m². The pulse duration is set to 70 ns with a frequency of 1 Hz.

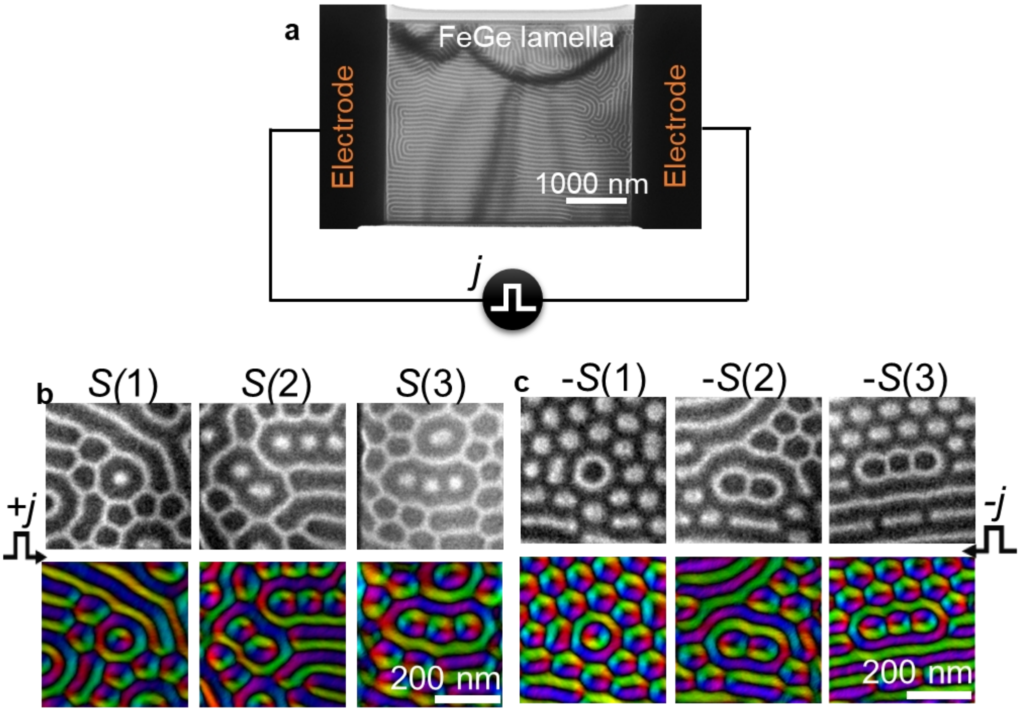

Supplementary Fig. 13 | Experimental observation of skyrmion bag in an 80-nm thick FeGe device (#2) at 100 K and zero field. (a) Overview of the device. (b) Representative skyrmion bags induced by positive currents. (c) Representative skyrmion bags induced by negative currents.

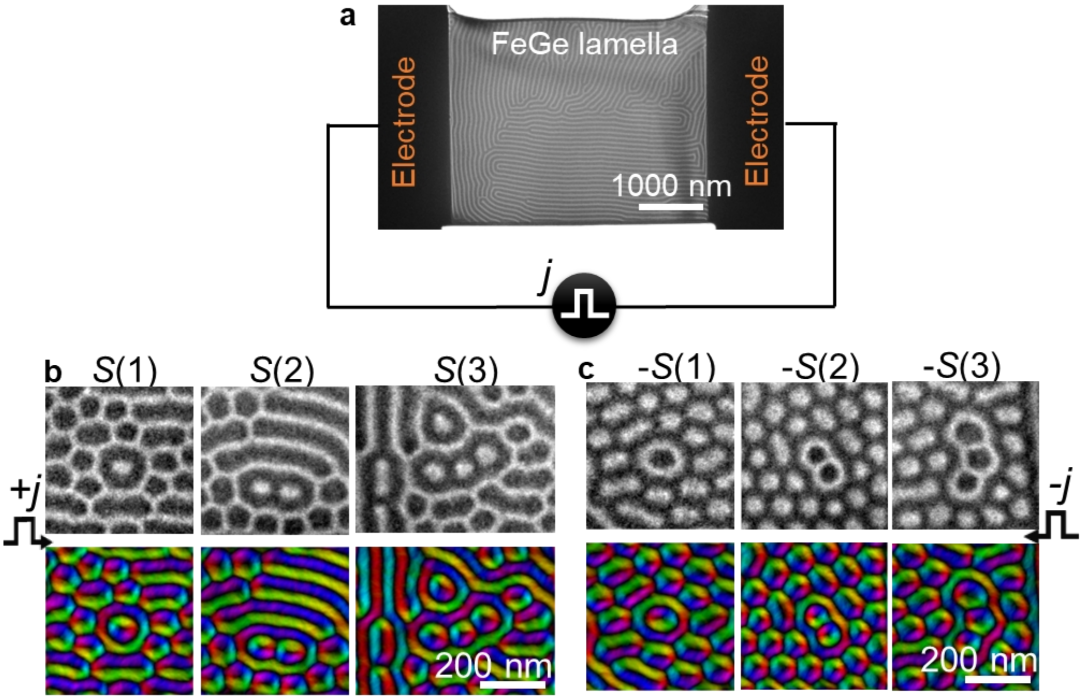

Supplementary Fig. 14 | Experimental observation of skyrmion bag in a 120-nm thick FeGe device (#3) at 100 K and zero field. (a) Overview of the device. (b) Representative skyrmion bags induced by positive currents. (c) Representative skyrmion bags induced by negative currents.

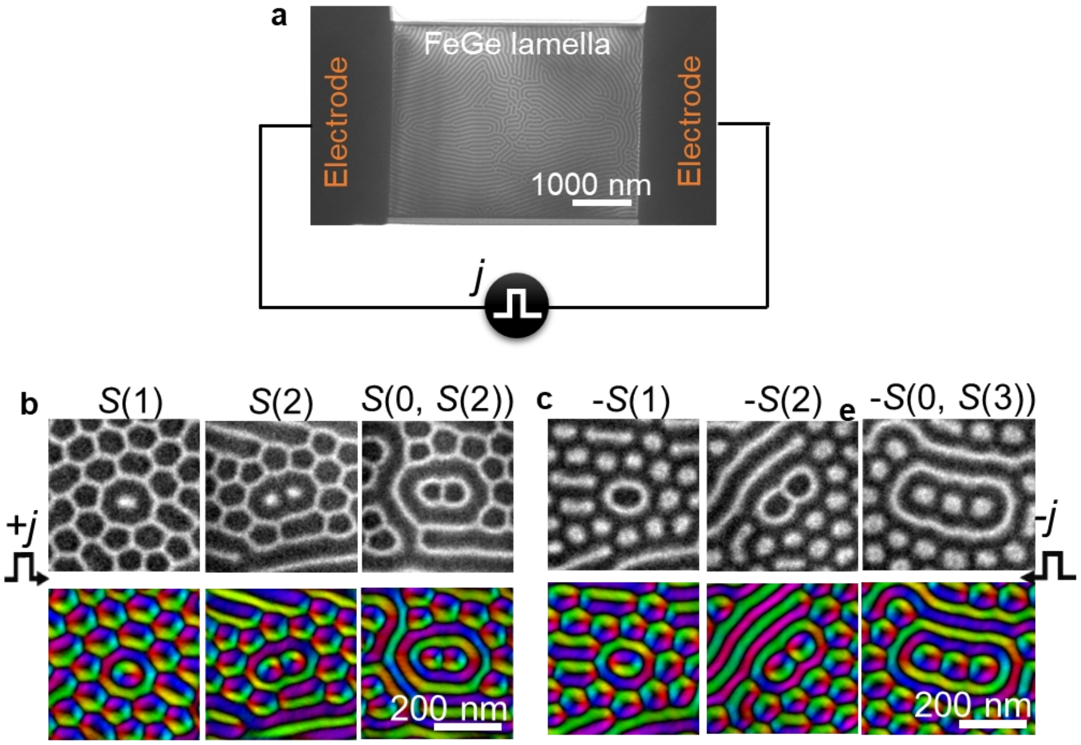

Supplementary Fig. 15 | Experimental observation of skyrmion bag in a 130-nm thick FeGe device (#4) at 100 K and zero field. (a) Overview of the device. (b) Representative skyrmion bags induced by positive currents. (c) Representative skyrmion bags induced by negative currents.

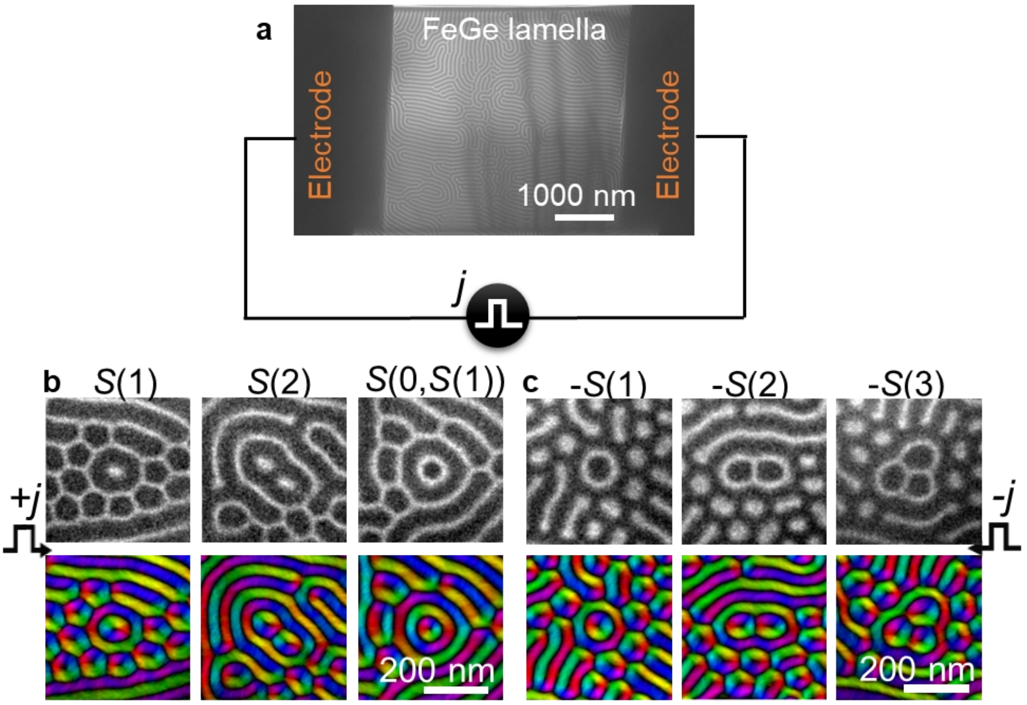

Supplementary Fig. 16 | Experimental observation of skyrmion bag in a 150-nm thick FeGe device (#5) at 100 K and zero field. (a) Overview of the device. (b) Representative skyrmion bags induced by positive currents. (c) Representative skyrmion bags induced by negative currents.

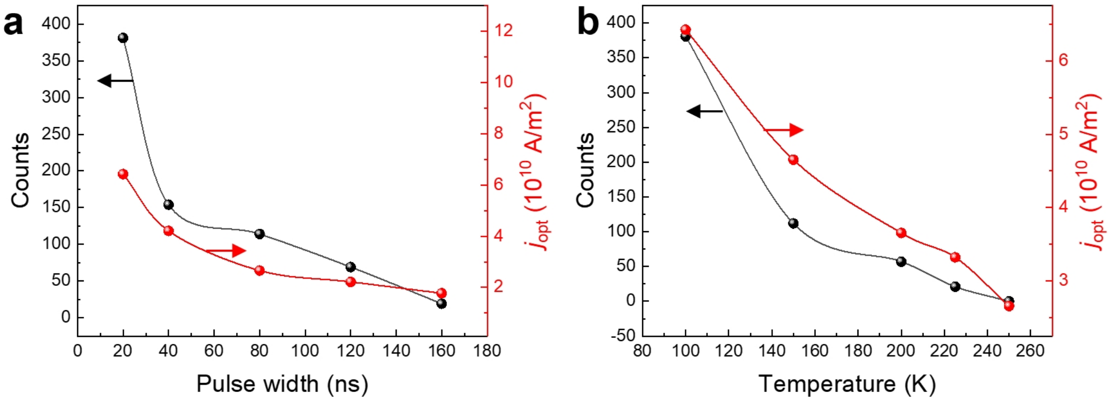

Supplementary Fig. 17 | Effect of pulse width and temperature on the current-induced creation of skyrmion bags. a The S(1) skyrmion bag emerging in 50 current cycles as a function of pulse width. The current during the cycle is chosen as the optimal current density. **b** The S(1) skyrmion bag emerging in 50 current cycles as a function of temperature. The current during the cycle is chosen as the optimal current density. The pulse width is 20 ns.

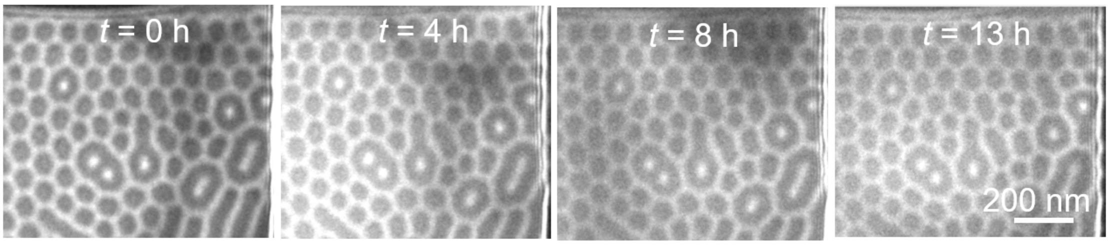

Supplementary Fig. 18 | Skyrmion bag stability over time under constant conditions (zero applied current, 95 K, and zero external magnetic field).

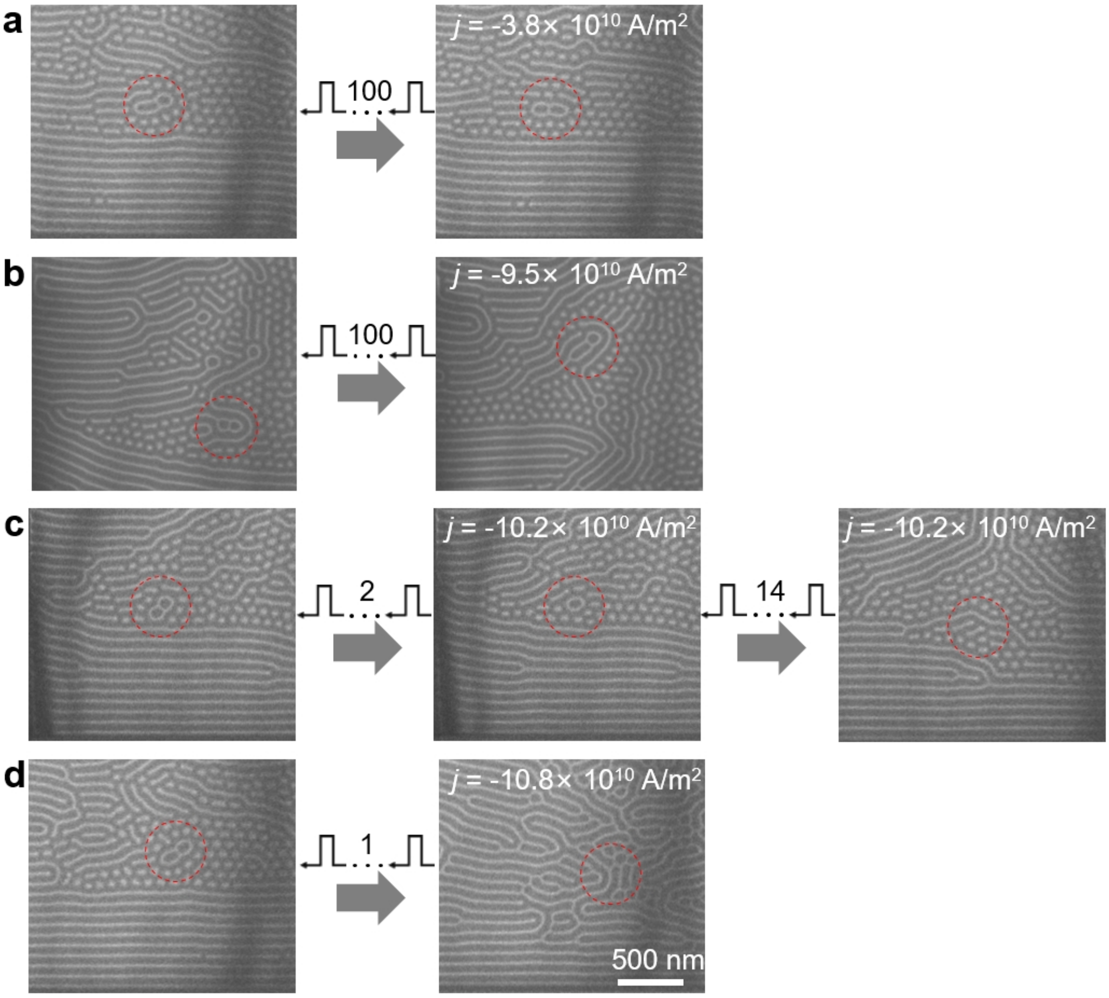

Supplementary Fig. 19 | Effect of current density on the stability of the −S(2) skyrmion bag for a fixed pulse width of 20 ns. The −S(2) skyrmion bag retains its topological state after applying 100 current pulses with densities of -3.8×10^{10} A/m² (a) and -9.5×10^{10} A/m² (b). At a current density of -10.2×10^{10} A/m² (c), the bag first transforms into a −S(1) bag after 2 pulses and subsequently into a Q = 1 skyrmion after 14 pulses. At a higher current density of -10.8×10^{10} A/m² (d), the state is reset after a single pulse.

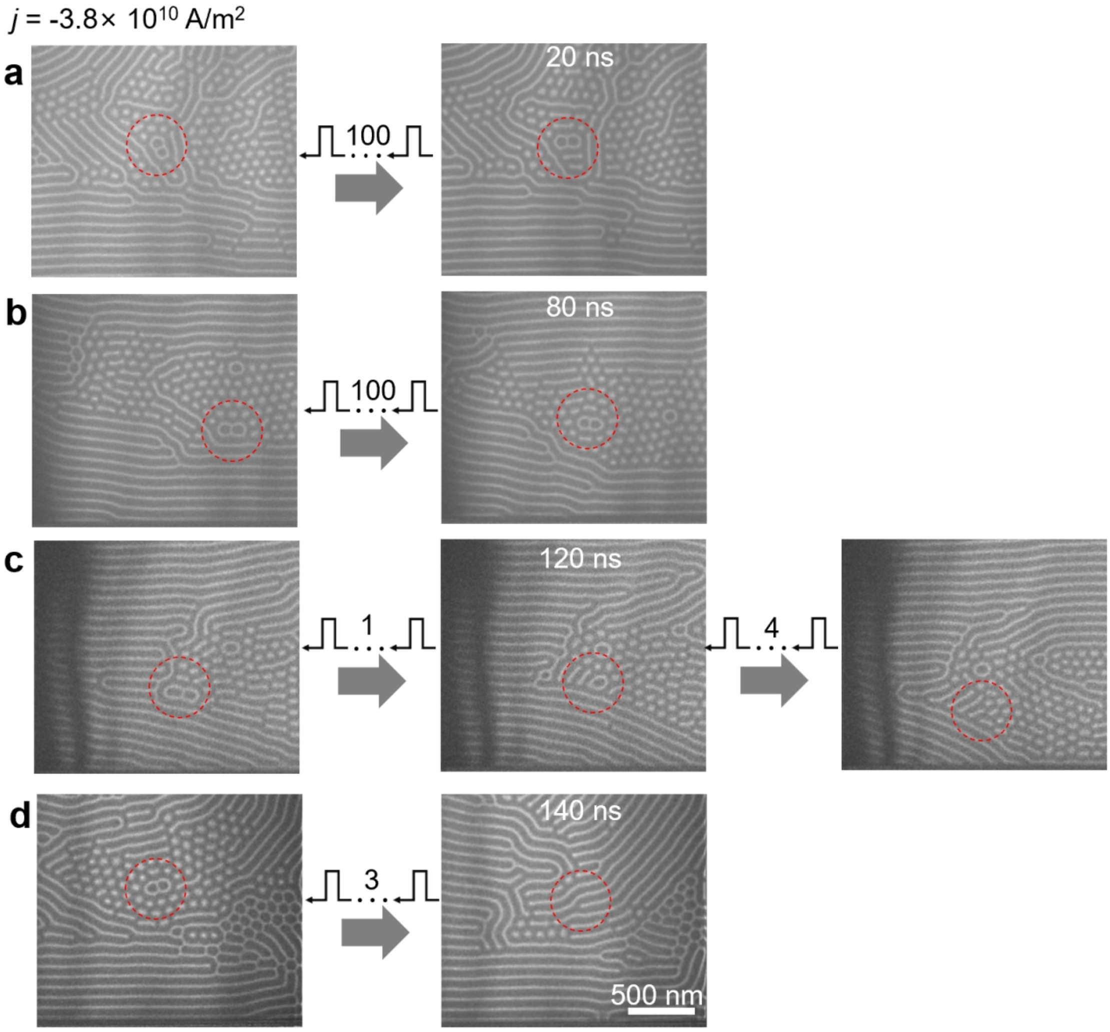

Supplementary Fig. 20 | Effect of pulse width on the stability of the $-S(2)$ skyrmion bag for a fixed current density of -3.8×10^{10} A/m^2. The $-S(2)$ skyrmion bag retains its topological state after applying 100 current pulses with widths of 20 ns (a) and 80 ns (b). At a pulse width of 120 ns (c), the bag first transforms into a $-S(1)$ bag after 1 pulse and then into a $Q = 1$ skyrmion after 4 pulses. At a width of 140 ns (d), the state is reset after 3 pulses.

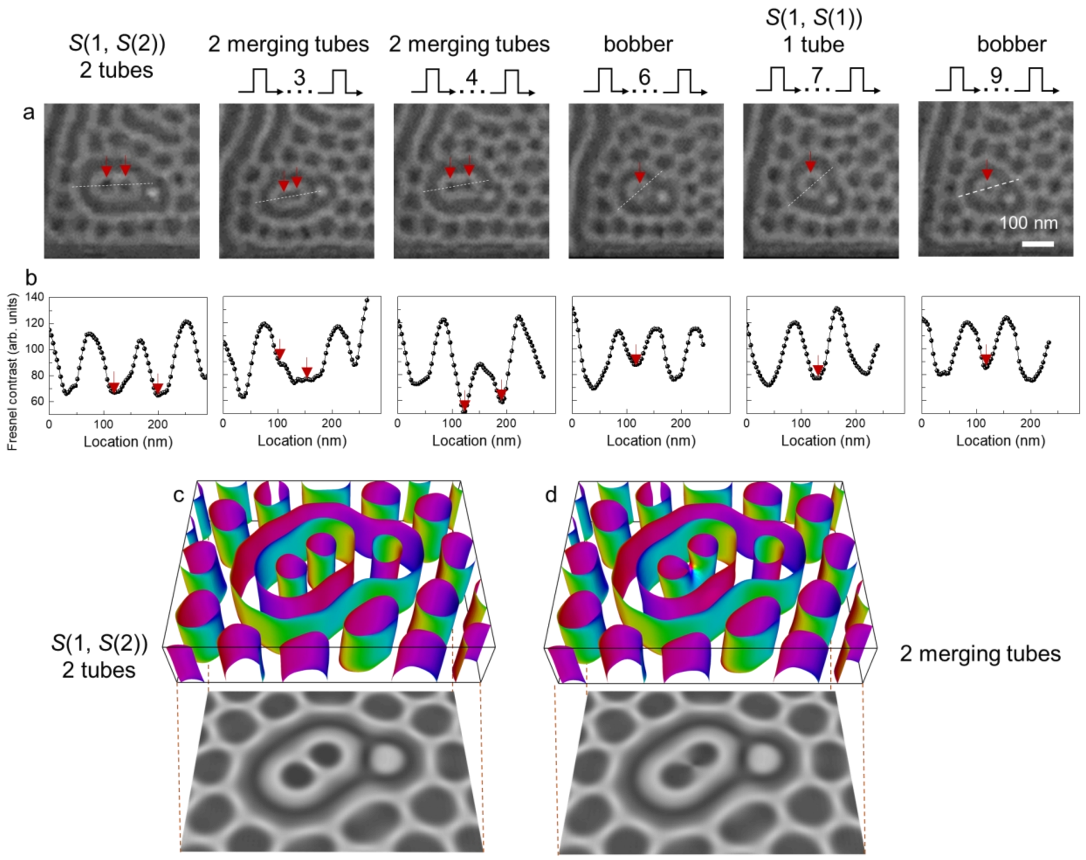

Supplementary Fig. 21 | Current-induced collapse of a nested *S*(1, *S*(2)) skyrmion bag. a Fresnel contrast during the collapse process. Defocused distance, −500 µm. **b** Line profiles of Fresnel contrast along the white dashed lines. The red arrows point out the location where the tubes are. **c, d** Simulated 3D magnetic iso-surface for $m_z = 0$ and corresponding defocused Fresnel contrasts of *S*(1, *S*(2)) nested bags with two individual tubes (c) and two merging tubes (d).

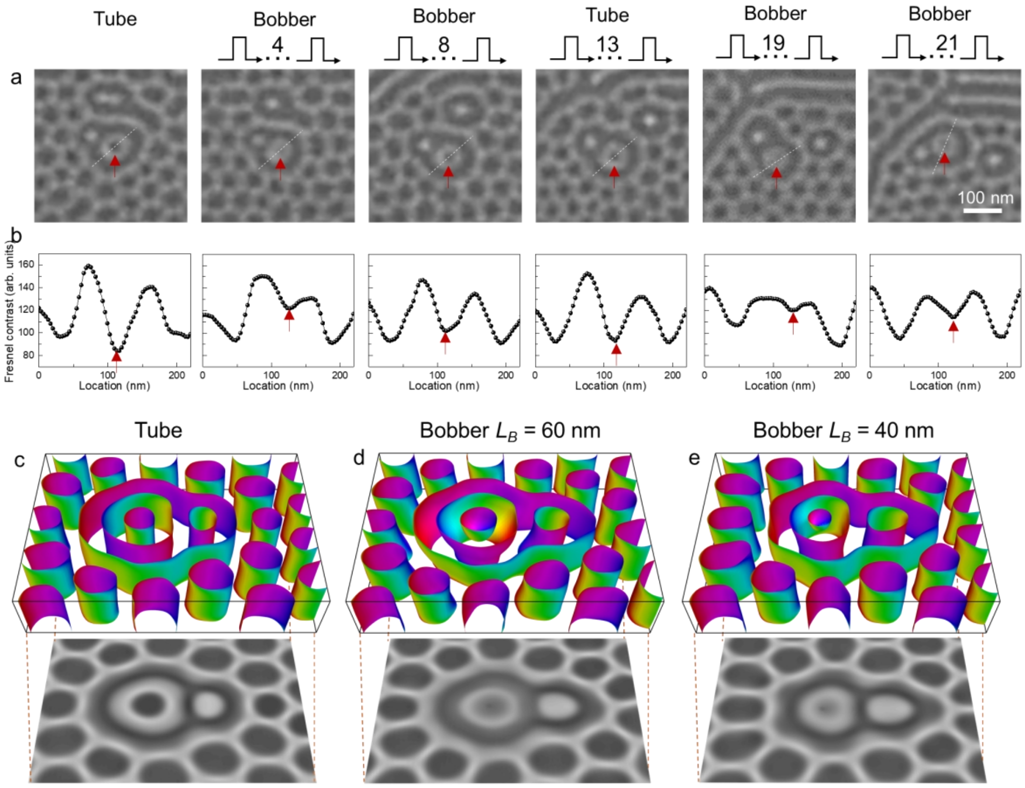

Supplementary Fig. 22 | Current-induced collapse of a nested *S*(1, *S*(1)) skyrmion bag. a Fresnel contrast during the collapse process. Defocused distance, −500 µm. **b** Line profiles of Fresnel contrast along the white dashed lines. The red arrows point out the location where the tubes/bobbers are. **c-e** Simulated 3D magnetic iso-surface for $m_z = 0$ and corresponding defocused Fresnel contrasts of *S*(1, *S*(1)) nested bags with 1 tube (c), 1 bobber with penetration depth L_B = 56 nm (d), and 1 bobber with penetration depth L_B = 34 nm (e).

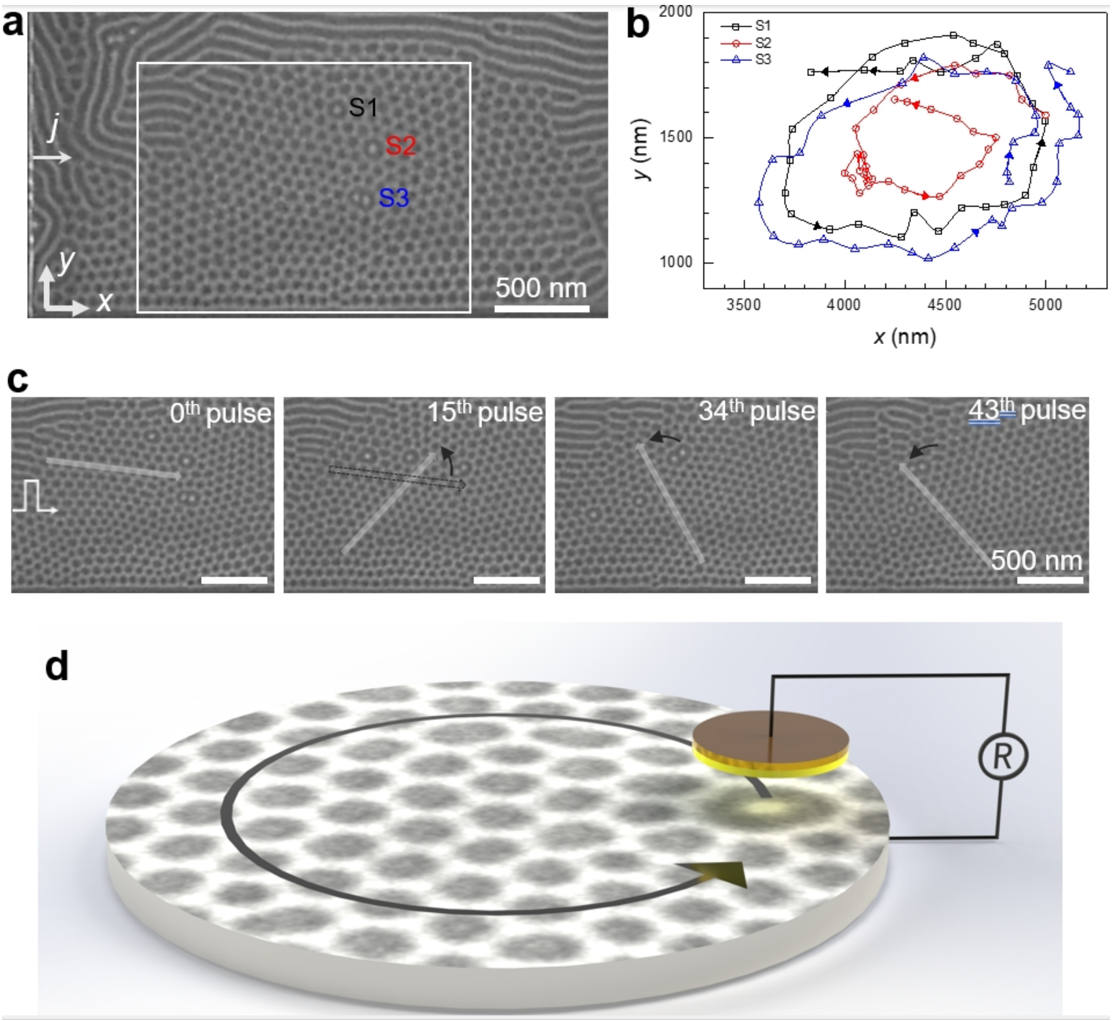

Supplementary Fig. 23 | Current-driven rotation of skyrmion lattice and skyrmion bags at zero magnetic field. a The overall view of the device. **b** The bag trajectories during the application of current pulses. S1, S2, and S3 represent the skyrmion bag as marked in (a). **c** Snapshots of the lattice and bag rotation driven by nanosecond current pulses. These Fresnel images were taken at a defocused distance of −500 μm. **d** Schematic device design based on skyrmion lattice rotation dynamics through the tunnel magnetoresistance detection of embedded skyrmion bags.

Supplementary References

1. Wilhelm, H. *et al.* Scaling study and thermodynamic properties of the cubic helimagnet FeGe. *Phys. Rev. B* **94**, 144424 (2016).
2. Zhao, X., Wang, S., Wang, C. & Che, R. Thermal effects on current-related skyrmion formation in a nanobelt. *Applied Physics Letters* **112**, 212403 (2018).
3. Arblaster, J. W. *Selected Values of the Crystallographic Properties of Elements*. (ASM International, 2018).
4. Strouse, G. F. Standard Platinum Resistance Thermometer Calibrations from the Ar TP to the Ag FP. *NIST* (2007).
