# Supplementary material for: Current-induced creation and dynamics of embedded magnetic skyrmion bags
Source: Nat Commun. 2026 Jun 11;17:7442. doi: 10.1038/s41467-026-74046-4 (PMC13408094; doi:10.1038/s41467-026-74046-4)
Supplement: Supplementary file 2 — Description of Additional Supplementary Files [file 41467_2026_74046_MOESM2_ESM.pdf]

Supplementary Movie captions:

Supplementary Movie 1 | Current-induced creation of *S*(1) and *S*(0, S(3)) magnetic skyrmion bags under zero magnetic field. The process is initiated at 0:06 s with two high-amplitude current pulses (5.5 × 10^{10} A/m^2) to establish the initial magnetic state. Following this, a train of current pulses at a density of 4.5 × 10^{10} A/m^2 drives the system dynamics (70 ns pulse duration, 1 Hz repetition rate).

Supplementary Movie 2 | Current-induced creation of *S*(1) and *S*(0, *S*(4)) magnetic skyrmion bags. The pulse duration is set to 70 ns with a frequency of 1 Hz, and the pulse amplitude is 4.5 × 10^{10} A/m^2.

Supplementary Movie 3 | Current-induced creation of *S*(1), *S*(2) and *S*(1, *S*(1)) magnetic skyrmion bags. The pulse duration is set to 70 ns with a frequency of 1 Hz, and the pulse amplitude is 4.5 × 10^{10} A/m^2.

Supplementary Movie 4 | Current-induced dynamics on helix under the stimuli of high-density current. The pulse duration is set to 70 ns, and the pulse amplitude is 5.6 × 10^{10} A/m^2.

Supplementary Movie 5 | Current-induced transformation from *S*(6) to *S*(5) bags. The pulse duration is set to 70 ns with a frequency of 1 Hz, and the pulse amplitude is 4.5 × 10^{10} A/m^2.

Supplementary Movie 6 | Current-induced transformation from *S*(3) to *S*(2) bags. The pulse duration is set to 70 ns with a frequency of 1 Hz, and the pulse amplitude is 4.5 × 10^{10} A/m^2.

Supplementary Movie 7 | Current-induced transformation from *S*(0, *S*(2)) to *S*(1) bags. The pulse duration is set to 70 ns with a frequency of 1 Hz, and the pulse amplitude is 4.5 × 10^{10} A/m^2.

Supplementary Movie 8 | Current-induced transformation from *S*(1, *S*(2)) to *S*(1, *S*(1)) bags. The pulse duration is set to 70 ns with a frequency of 1 Hz, and the pulse amplitude is 4.5×10^{10} A/m^2.

Supplementary Movie 9 | Current-induced dynamics of the *S*(1, *S*(1)) bag. The pulse duration is set to 70 ns with a frequency of 1 Hz, and the pulse amplitude is 4.5×10^{10} A/m^2.
