# Supplementary material for: Current-induced creation and dynamics of embedded magnetic skyrmion bags
Source: Nat Commun. 2026 Jun 11;17:7442. doi: 10.1038/s41467-026-74046-4 (PMC13408094; doi:10.1038/s41467-026-74046-4)
Supplement: Supplementary file 12 — Transparent Peer Review file [file 41467_2026_74046_MOESM12_ESM.pdf]

nature portfolio

Peer Review File

Current-induced creation and dynamics of embedded magnetic skyrmion bags

Corresponding Author: Professor Jin Tang

This file contains all reviewer reports in order by version, followed by all author rebuttals in order by version.

Version 0:

Reviewer comments:

Reviewer #1

(Remarks to the Author)
The skyrmions with high topological numbers can be the potential candidates for spintronics applications, such as multilevel memory, neuromorphic computing. For practical application, it is quite essential to create and manipulate the high-Q skyrmions on demand. Electrical current spin transfer torques could be a potential method for this. Although previous simulation study suggested the creation of multiple-Q skyrmions using electrical methods, experimental confirmation remained elusive. In the present study, authors have investigated current-induced formation of skyrmion bags in a 100 nm thick FeGe nanolayer. The helical ground state of magnetization is transformed into skyrmion bags under the application of a current-induced spin-transfer torque, as observed by Lorentz Transmission electron microscopy. Authors further demonstrate electrically-induced transitions between skyrmion bags of different topological charge (Q), leading to the stabilization of complex three-dimensional topological structures, including magnetic monopoles and bobbers. The experimental observations are corroborated with micromanetic simulations.
I believe the present work will significantly advance the research field. Thus, the work seems to be suitable for Nature Communications. However, the current manuscript is lacking several information about the experiment and, physics behind the observed phenomena. Thus, a major revision is required before I can recommend it for publication.
[1] The number of created skyrmion bags and nested skyrmions is way smaller than the number of normal skyrmions. It appears that the creation of high-Q skyrmions is very challenging. Authors should explain in the manuscript the key challenges to create multiple-Q skyrmions by electrical method. Under what conditions are they formed, i.e., what are the key factors to decide the formation of high-Q skyrmions?
[2] How did the authors come to the conclusion that the current induced spin-transfer torques (STT) are taking the leading role in generating skyrmion bags in their sample? The authors have not talked about it in detail. What are the roles of in-plane and out-of-plane torques? Why did the authors neglect the possible role of Oersted field? Authors should calculate the generated Oersted field for the applied current densities.
[3] Did the authors perform the measurements on a few devices to confirm the reproducibility of the obtained data? Please comment on it. Also, the “counts” and “probability” in Figure 4 should have error bars if the numbers are averaged out from several measurements performed on several devices.
[4] Some additional queries from Figure 4. What is the area of the sample from which the number of high-Q skyrmions was counted? What is the number of normal skyrmions (Q=1, -1)? The authors did not mention about the size of the skyrmions. The nested skyrmions have significantly high energy. So, why do they form? An explanation about the probability calculation is needed.
[5] Why did the authors choose FeGe material for this study? Also, why was 100 nm thickness of FeGe chosen? I was wondering whether the material parameters, measurement temperature, and sample thickness have crucial roles in the formation of high-Q skyrmions, i.e., the number of high-Q skyrmions.
[6] How was the pulse width of the current decided? Is there any effect of pulse width on the formation of skyrmion bags?
[7] On page 9, the authors commented that “In our present experiments, the skyrmion bag is stabilized within a skyrmion lattice”. How did the authors confirm that the skyrmion bags are stabilized over skyrmion bundles in the experiment?
[8] The authors explained that the high-Q skyrmions are transformed into lower-Q skyrmions after the application of current pulses. If it is true, how are the high-Q skyrmions actually formed, after how many pulses? What are the effects of current density, polarity, and pulse width in this case? In the manuscript, the given explanations are quite confusing.
[9] On page 6, the authors mentioned “Once current is applied, the spin-transfer torque forces the formation of a horizontal helix with a q-vector perpendicular to the current orientation, as experimentally identified (Fig. 2b). However, we can always observe a perpendicular helix whose top ends are pinned from the geometrical edges”. What is the meaning of “q-vector”

here? Authors are requested to clarify these statements. Also, the formation process of skyrmions from perpendicular helix is not well explained in the manuscript. On the next page, the authors mentioned “The long helix could undergo the integral crash forces of external individual skyrmion moving to the bottom ends, contributing to the formation of individual S(n) bags”. This statement is also not very clear to me.
[10] Some more comments:
(i) What is the sign convention of the applied current pulses?
(ii) On page 6, the authors mentioned that “Notably, the outermost magnetizations of both S(n) and S(n1, S(n2)) bags exhibit an out-of-plane up orientation, as shown in Fig. 1c”. Should it be “out-of-plane down orientation”?
(iii) How long do the high-Q skyrmions remain stable after formation?

Reviewer #2

(Remarks to the Author)
The manuscript reports the observation of skyrmion bags that spontaneously emerge during the current-induced transformation from the chiral labyrinth domain state (called by the authors a spin spiral) into the skyrmion lattice. In essence, this effect stems from the formation of closed domains whose direction is inverted compared to that favored by the combination of dominant domain termination and current direction. The closed domain traps inverted skyrmions inside. The authors found that a varied number of skyrmions can be trapped, and observed current-driven transformations among different skyrmion populations in the bag.
This work is part of a series of papers published by the same authors on the subject of current-induced skyrmion generation. Notably, Nano Letters, 22, (2022) cited as Ref. 35 in the manuscript showed current-induced generation of skyrmions in the same material (FeGe) and the same structure, also at zero magnetic field. Ref. 25 Nano Letters, 25 (2025) demonstrated stable skyrmion bags in FeGaTe defined by current sequence with assistance from the field.
The main claim of novelty in the present manuscript is that skyrmion bags are generated by current without assistance from the field. I find the presented results clear, convincing, aesthetically pleasing, easy to popularize for the general public. They advance the field of skyrmionics. Thus, I expect that this work will be cited and will have an impact both on the field and on the broader scientific community.
On the technical level, my main concern is that the domain termination and direction relative to current determines the type of current-generated skyrmions. Consequently, generation of skyrmion bags starting from a random magnetic configuration without magnetic field assistance is an uncontrolled process. In my opinion, this randomness diminishes the practical significance of the presented results, under the assumption that skyrmionic devices can, in principle, become practical.
From the more fundamental point of view, I feel that the manuscript is a relatively modest advancement compared to the results previously published by the group. This is evidenced by the fact that the manuscript does not provide a new theoretical analysis. Therefore, I am overall not convinced that the level of novelty or practical significance warrant publication in Nat. Comm.

Reviewer #3

(Remarks to the Author)
Referee Report on "Current-induced creation and dynamics of embedded magnetic skyrmion bags"
by Yaodong Wu, Jialiang Jiang, Lingyao Kong, Meng Shi, Shouguo Wang, Mingliang Tian, Haifeng Du, and Jin Tang

This manuscript reports the current-induced creation and dynamics of magnetic skyrmion bags in FeGe nanoplates using in-situ Lorentz TEM.

They performed well organized experiments and compared with the simulation.
I believe these results are, in principle, worthy of publishing on Nature Communications.
While the realization of all-electrical control of high-Q topological textures is significant and timely,
several critical points regarding the deterministic control, thermal effects, and 3D dynamics remain insufficiently addressed.
The following points should be clarified before publication.

-Stochasticity vs. Deterministic Control
The authors demonstrate that skyrmion bags can be generated via current pulses.
However, the process appears highly stochastic.
For potential applications in multi-state memory, deterministic switching between specific topological states (e.g., $S(n)$ to $S(n \pm 1)$) is essential.
The manuscript lacks a clear phase diagram or protocol that correlates specific pulse parameters (amplitude, duration, or frequency) with the resulting topological charge Q in a predictable manner.

-Quantitative Separation of Joule Heating
The study attributes the bag formation primarily to Spin-Transfer Torque (STT).
However, given the high current densities used ($j \approx 10^{10} \text{A/m}^2$), Joule heating is inevitable.
The authors should provide a more rigorous quantitative analysis or control experiment to distinguish the purely torque-driven dynamics from thermally-activated processes.
Does the temperature rise during pulsing assist in overcoming the topological energy barriers?

-Ambiguity in 3D Topological Dynamics
The interpretation of the annihilation of internal skyrmions through magnetic monopoles or chiral bobbers relies heavily on micromagnetic simulations.
As Lorentz TEM provides only a 2D projection of the magnetization, the evidence for these 3D processes remains indirect. The authors should discuss the limitations of their 2D observations in describing 3D tube-breaking events and provide more robust justification for their proposed 3D models.

-Electrical Detection (Read-out) Mechanism
While the "writing" and "manipulation" of skyrmion bags are discussed, the "reading" aspect is entirely absent.
The authors should comment on how these varying topological charges could be detected electrically (e.g., via the Topological Hall Effect or TMR) without relying on electron microscopy, which is crucial for any practical spintronic device.

-Material and Temperature Generality
The experiments are conducted on FeGe at specific temperatures (around 220 K).
The authors should address whether the observed current-induced bag formation is a universal phenomenon applicable to room-temperature chiral magnets or multilayer systems with interfacial Dzyaloshinskii-Moriya Interaction (DMI).

-The stability and retention time of the generated skyrmion bags after the current is turned off should be quantified.

Version 1:

Reviewer comments:

Reviewer #1

(Remarks to the Author)
I am satisfied with the authors responses to my queries. I have also noticed that the authors have made appropriate changes in the revised manuscript. I believe the manuscript is now suitable for publication.

Reviewer #2

(Remarks to the Author)
The authors have provided detailed rebuttals to all the criticisms raised by the three referees, and introduced the corresponding revisions in the manuscript. In my opnion, these rebuttals/changes are thorough and convincing. As I wrote in my original review, as far as the field of skyrmionics is concerned, the manuscript provides a sizable contribution and will likely be well-received. I believe it should be published.

However I should note that the comments of the reviewers and the discussion in the manuscript seem to be based on the implicit assumption that there are widely accepted pathways for practical applications of skyrmionic devices that can materialize once cetain conditions such as room-temperature operation mentioned by the Referee C, or perhaps some stability/efficiency conditions mentioned by Referee A, are satisfied. However, I am personally not aware of any concrete proposals for such practical devices, or estimates of how they would compete with existing technologies, and neither have I seen any substantial analysis of what properties, if any, of skyrmion-hosting materials would satisfy the application requirements.

Open Access This Peer Review File is licensed under a Creative Commons Attribution 4.0 International License, which permits use, sharing, adaptation, distribution and reproduction in any medium or format, as long as you give appropriate credit to the original author(s) and the source, provide a link to the Creative Commons license, and indicate if changes were made.
In cases where reviewers are anonymous, credit should be given to 'Anonymous Referee' and the source.
The images or other third party material in this Peer Review File are included in the article's Creative Commons license, unless indicated otherwise in a credit line to the material. If material is not included in the article's Creative Commons license and your intended use is not permitted by statutory regulation or exceeds the permitted use, you will need to obtain permission directly from the copyright holder.
To view a copy of this license, visit https://creativecommons.org/licenses/by/4.0/

Manuscript ID: NCOMMS-25-13186-T

"*Current-controlled creations, deletions, and topological transformations of a single magnetic antiskyrmion in nanostructured cells*" by Y. Wu *et al.*

Note that all page numbers and references without special instructions refer to the newly revised manuscript attached to this response and not to the original version submitted. The added contents are marked in red in the revised manuscript.

Response to Reviewer #1:

Reviewer #1: The skyrmions with high topological numbers can be the potential candidates for spintronics applications, such as multilevel memory, neuromorphic computing. For practical application, it is quite essential to create and manipulate the high-Q skyrmions on demand. Electrical current spin transfer torques could be a potential method for this. Although previous simulation study suggested the creation of multiple-Q skyrmions using electrical methods, experimental confirmation remained elusive. In the present study, authors have investigated current-induced formation of skyrmion bags in a 100 nm thick FeGe nanolayer. The helical ground state of magnetization is transformed into skyrmion bags under the application of a current-induced spin-transfer torque, as observed by Lorentz Transmission electron microscopy. Authors further demonstrate electrically-induced transitions between skyrmion bags of different topological charge (Q), leading to the stabilization of complex three-dimensional topological structures, including magnetic monopoles and bobbers. The experimental observations are corroborated with micromanetic simulations.
I believe the present work will significantly advance the research field. Thus, the work seems to be suitable for Nature Communications. However, the current manuscript is lacking several information about the experiment and, physics behind the observed phenomena. Thus, a major revision is required before I can recommend it for publication.

Response: We thank you very much for your time and effort in reviewing our manuscript. Below, please find our detailed response to each of your insightful comments.

Comment 1: The number of created skyrmion bags and nested skyrmions is way smaller than the number of normal skyrmions. It appears that the creation of high-Q skyrmions is very challenging. Authors should explain in the manuscript the key challenges to create multiple-Q skyrmions by electrical method. Under what conditions are they formed, i.e., what are the key factors to decide the formation of high-Q skyrmions?

Response: The key factors determining the formation of high-*Q* skyrmion bags are the coexistence and spatial arrangement of skyrmions with opposite topological charges (*Q*

= 1 and $Q = -1$). A skyrmion bag consists of inner skyrmions (*e.g.*, $Q = 1$) enclosed by a spiral boundary with opposite sign (*e.g.*, $Q = -1$).

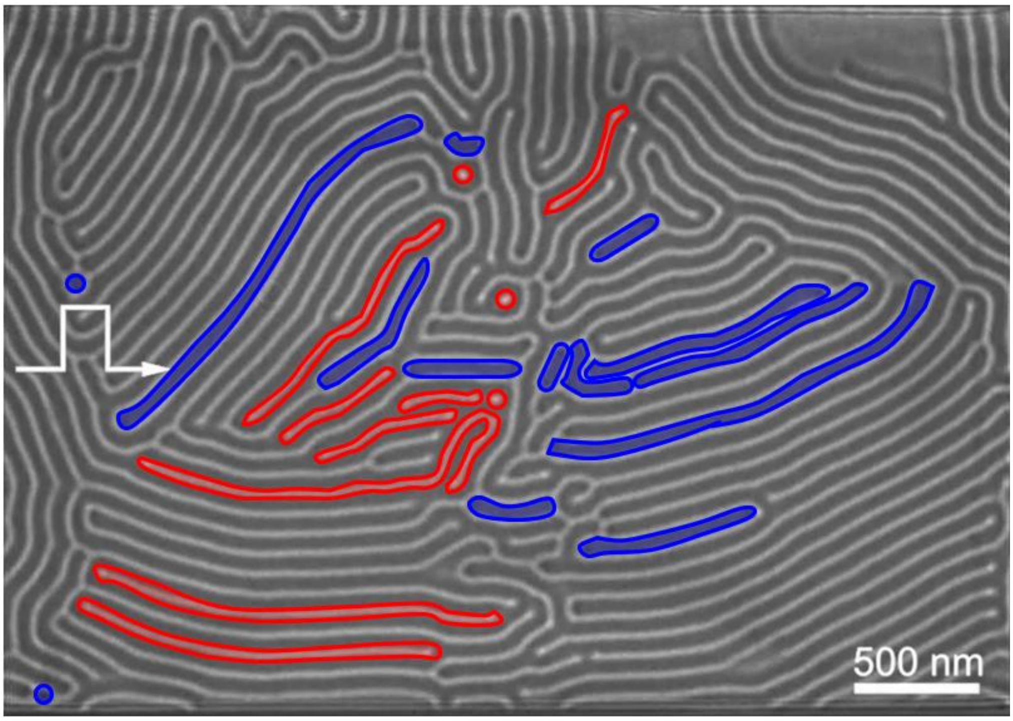

Fig. R1 (*i.e.*, Fig. 2a in the main text) Initial helix domains after applying a single high pulsed current. Blue and red boxes represent the closure helix domains topologically equivalent to skyrmions with $Q = 1$ and -1, respectively.

In our cases, the creation process involves two stages: First, a high-density current pulse is applied to reset the magnetic domains, generating a disordered state containing isolated skyrmions of both polarities ($Q = 1$ and -1), as shown in Fig. R1. Subsequently, a low-density current drives the system toward a skyrmion lattice with a preferred topological charge, e.g., $Q = -1$. During this process, pre-existing $Q = 1$ skyrmions can become surrounded by the emerging $Q = -1$ lattice, forming isolated skyrmion bags embedded in the uniform lattice background. This mechanism naturally yields a much larger number of ordinary skyrmions compared to skyrmion bags, as the latter require specific local conditions—namely, an isolated skyrmion of opposite charge to act as a nucleus.

We have clarified this point in the revised manuscript (Lines 147–152): "*Under continuous current stimulation, skyrmions are generated to form a lattice with a uniform topological charge, e.g., $Q = -1$. In contrast, skyrmion bags consist of internal skyrmions with $Q = 1$, which predominantly originate from the initial disordered state (Fig. 2a). As a result, skyrmion bags appear as isolated objects surrounded by a skyrmion lattice, and their number is inherently limited by the availability of oppositely charged seeds.*"

Comment 2: How did the authors come to the conclusion that the current induced spin-transfer torques (STT) are taking the leading role in generating skyrmion bags in their sample? The authors have not talked about it in detail. What are the roles of in-plane and out-of-plane torques? Why did the authors neglect the possible role of Oersted field? Authors should calculate the generated Oersted field for the applied current densities.

Response: We thank you for raising this important point regarding the detailed role of current-induced effects. We agree that a thorough analysis of the torques and possible field contributions is essential to justify our modeling approach and experimental interpretation. Below, we provide a comprehensive response structured into three key aspects.

Torque contributions. In our simulations, we consider the Zhang-Li spin-transfer torque (STT) as the primary driving mechanism for skyrmion dynamics. This choice is motivated by the material system under investigation: our devices are based on the helimagnet FeGe and are not capped with heavy-metal layers. Consequently, out-of-plane spin-orbit torques, which arise from the spin Hall effect in heavy-metal/ferromagnet bilayers, are absent. The Zhang–Li STT, which originates from the adiabatic coupling between conduction electron spins and the local magnetization, is widely accepted as the dominant current-induced torque in metallic helimagnets such as FeGe.

Oersted field analysis. Following your nice suggestion, we have quantitatively evaluated the possible role of the Oersted field generated by the applied current. We performed Multiphyisc Field physics simulations to compute the Oersted field distribution at the current density used in our study (4.5×10^{10} A·m^{-2}), as shown in Fig. R2. The Oersted field is found to be predominantly in-plane within the device, with a maximum magnitude of approximately 2.86 mT. Notably, the in-plane field orientation differs between the top and bottom surfaces due to the asymmetric current distribution—a characteristic feature of Oersted fields in thin-film geometries. Comparison with skyrmion nucleation conditions. In our FeGe devices, skyrmion creation typically requires an out-of-plane magnetic field with a magnitude on the order of 100 mT. The Oersted field, in contrast, is (i) purely in-plane, (ii) an order of magnitude smaller, and (iii) exhibits spatial variations that average out over the device volume. These characteristics make it incapable of nucleating skyrmions.

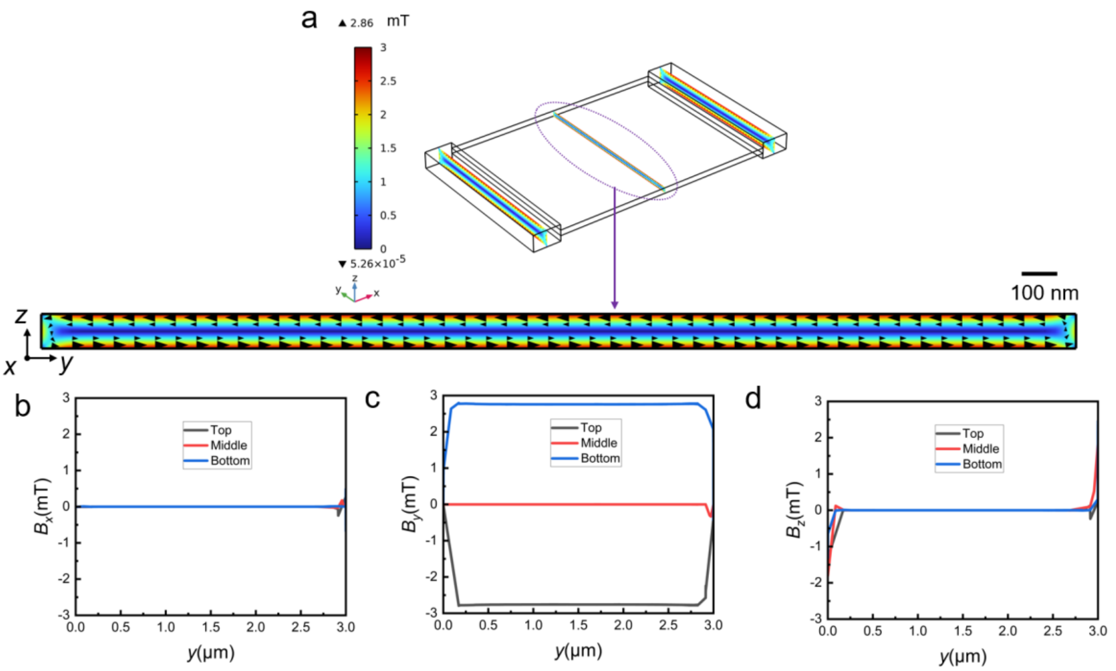

Fig. R2 (*i.e.*, Supplementary Fig. 9) Simulated Oersted field distribution of a 100-nm-thick FeGe microdevice at a current density of 4.5×10^{10} $A\cdot m^{-2}$ applied along the x axis. (a) The Oersted field distribution in the middle layer. The color represents the magnitude of the magnetic flux density. (b)-(d) Dependence of the Oersted field components on position y at the middle layer: B_x (b), B_y (c), and B_z (d).

The force analysis and symmetry consideration. To further elucidate the governing mechanism, we performed experiments correlating current orientation with helix stripe polarity and pinning configuration. Under the application of current, the helix stripe is either stretched or compressed depending on the topological charge Q and the polarity of the helix stripe. For example, as shown in Fig. R3a, when a helix with downward polarity is stretched, the meron at its end splits off under maximal stretch, generating a skyrmion with $Q = -1$; in contrast, compressed helices remain attached.

For a vertical helix pinned at the top side, a positive current leads to the formation of $Q = -1$ skyrmions and $S(n)$ skyrmion bags, while a negative current yields $Q = 1$ skyrmions and $-S(n)$ bags. Conversely, when the helix is pinned at the bottom side, the topological charge sign reverses under the same current polarity. These four predicted cases are systematically summarized in Supplementary Fig. 10 and are well reproduced in our experiments (Supplementary Fig. 11), providing strong evidence that the observed phenomena are governed by STT rather than other current-induced effects.

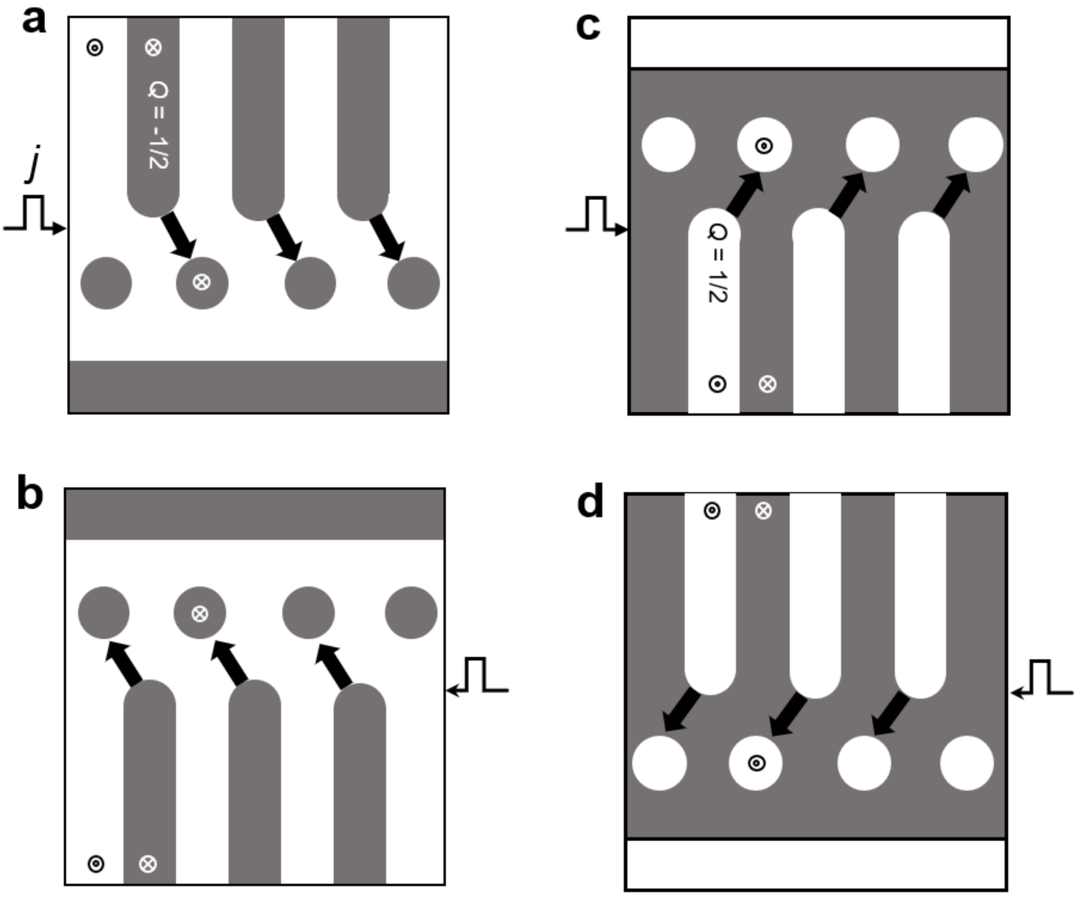

Fig. R3 (*i.e.*, Supplementary Fig. 10) Schematic skyrmion generation rule based on force and symmetry analysis. Four cases are considered depending on the helix pinning ends and current orientation. Black and white represent out-of-plane magnetization down and up, respectively. Black arrows indicate the motion direction of helix ends, equivalent to merons driven by STT. **a** vertical helix pinned at the top ends, positive current; **b** pinned at the bottom ends, positive current; **c** pinned at the bottom ends, negative current; **d** pinned at the top ends, negative current. Starting from (a), other cases in (b), (c), and (d) can be obtained by applying time-reversal and/or combined rotation of spin and space by 180 degrees to (a).

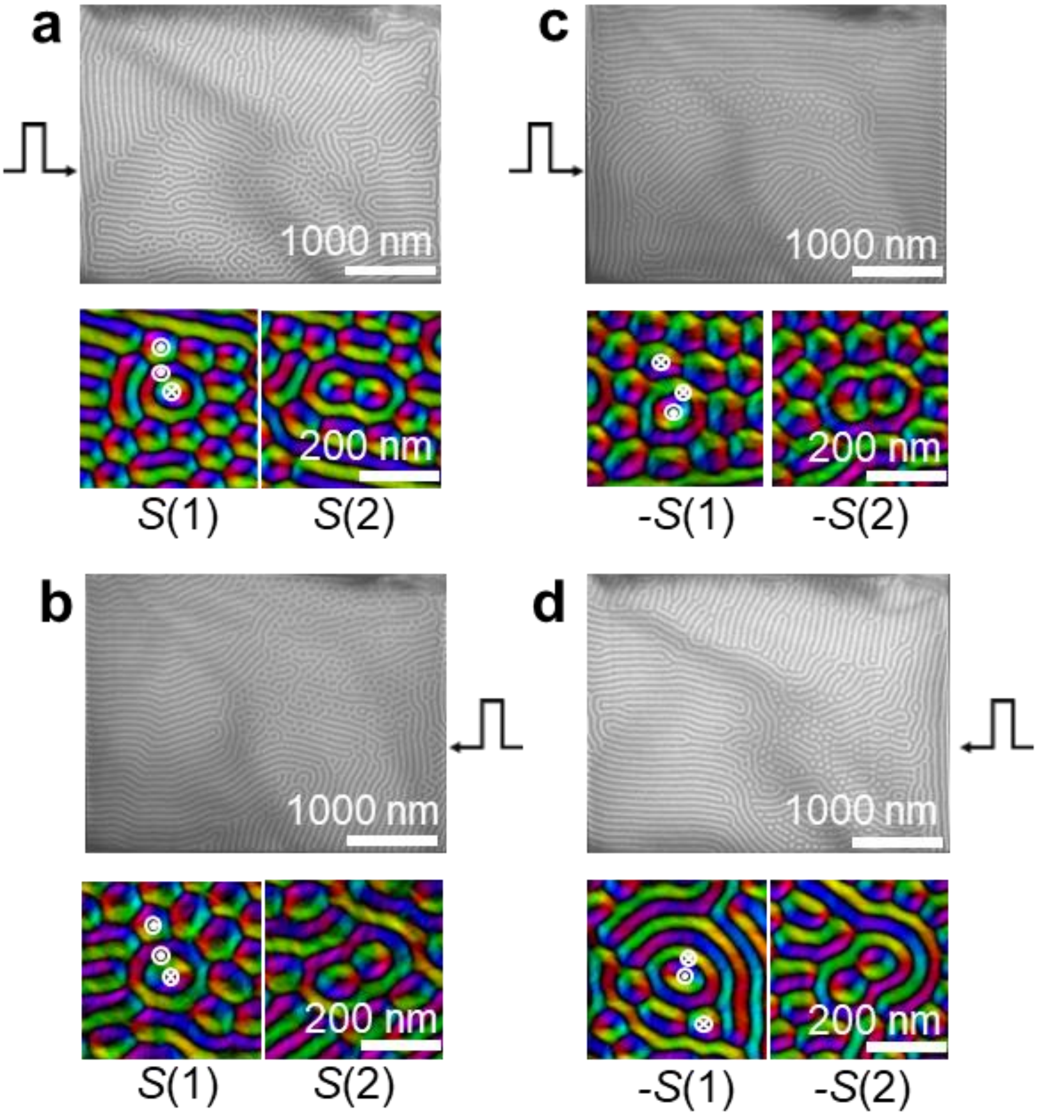

Fig. R4 (*i.e.*, Supplementary Fig. 11) Experimental demonstration of the skyrmion generation rule based on force and symmetry analysis. Fresnel images were taken at a defocused distance of −500 μm, representing the pinning ends of the vertical helix. Representative in-plane magnetic configuration of skyrmion bags and skyrmions.

To address physical mechanism comprehensively, we have added the following discussion to the revised manuscript (Lines 212–227): " *To assess the possible influence of the Oersted field, we performed multiphysics simulations of the field distribution at the applied current density (Supplementary Fig. 9). The Oersted field is predominantly in-plane, with a maximum magnitude of approximately 2.86 mT, and its orientation differs between the top and bottom surfaces due to the asymmetric current distribution. In our FeGe devices, skyrmion creation typically requires an out-of-plane field on the order of 100 mT. The Oersted field is therefore (i) purely in-plane, (ii) an order of magnitude smaller, and (iii) spatially varying, making it incapable of nucleating*

skyrmions. Furthermore, symmetry and force analysis yield four distinct STT-driven dynamical behaviors depending on the pinning ends of the vertical helix and the current orientation (Supplementary Fig. 10). Our experiments reproduce all four predicted schemes (Supplementary Fig. 11). Consequently, we conclude that STT is the primary effect determining the topological sign of current-induced skyrmion bags and skyrmions, while Joule heating plays at most an auxiliary role. Since our devices are based on FeGe without adjacent heavy-metal layers, spin–orbit torques are absent, and the current-induced torque is dominated by the in-plane Zhang–Li STT[30]. "

Comment 3: Did the authors perform the measurements on a few devices to confirm the reproducibility of the obtained data? Please comment on it. Also, the "counts" and "probability" in Figure 4 should have error bars if the numbers are averaged out from several measurements performed on several devices.

Response: Yes, we have performed measurements on multiple devices, as shown in Fig. R5-R8. The device thickness varies from 80 to 150 nm. These devices show similar current-induced dynamics and creation of skyrmion bags.

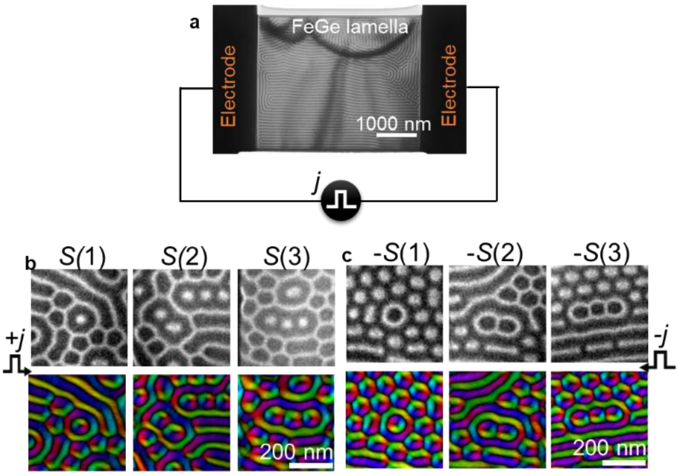

Fig. R5 (*i.e.*, Supplementary Fig. 13) Experimental observation of skyrmion bag in a 80-nm thick FeGe device at 100 K and zero field. (a) Overview of the device. (b) Representative skyrmion bags induced by positive currents. (c) Representative skyrmion bags induced by negative currents.

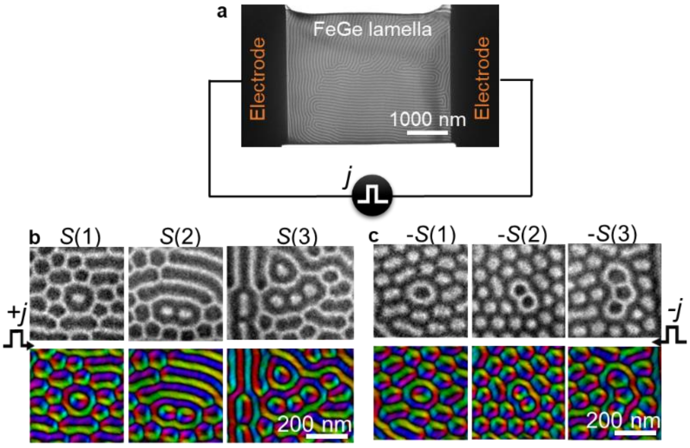

Fig. R6 (*i.e.*, Supplementary Fig. 14) Experimental observation of skyrmion bag in a 120-nm thick FeGe device at 100 K and zero field. (a) Overview of the device. (b) Representative skyrmion bags induced by positive currents. (c) Representative skyrmion bags induced by negative currents.

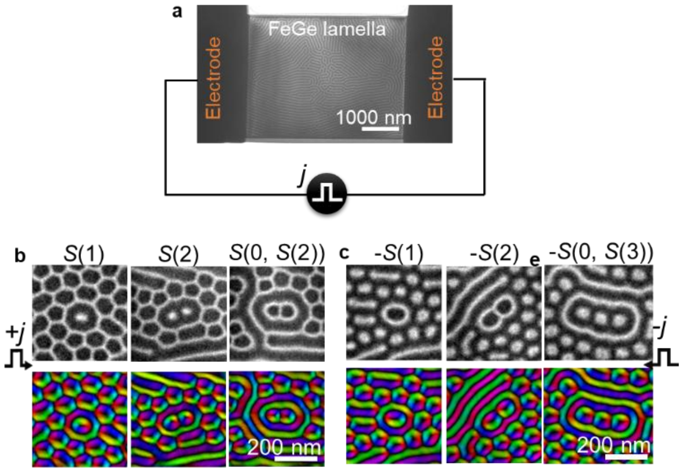

Fig. R7 (*i.e.*, Supplementary Fig. 15) Experimental observation of skyrmion bag in a 130-nm thick FeGe device at 100 K and zero field. (a) Overview of the device. (b) Representative skyrmion bags induced by positive currents. (c) Representative skyrmion bags induced by negative currents.

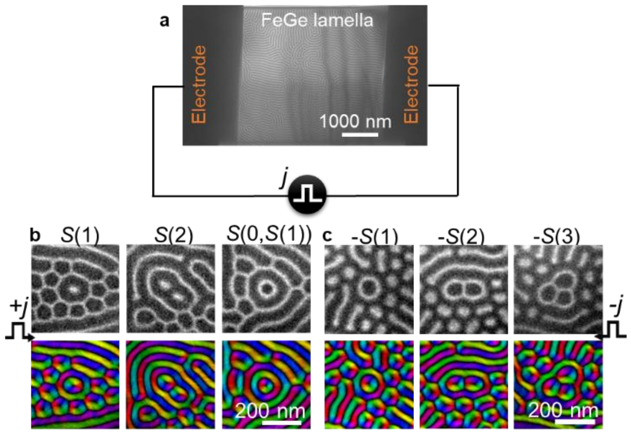

Fig. R8 (*i.e.*, Supplementary Fig. 16) Experimental observation of skyrmion bag in a 150-nm thick FeGe device at 100 K and zero field. (a) Overview of the device. (b) Representative skyrmion bags induced by positive currents. (c) Representative skyrmion bags induced by negative currents.

For the statistical counts, we fabricate three devices with similar length, width, and thickness of about 4 µm, 3 µm, and 100 nm, respectively, at 100 K. The statistical counts of skyrmion bags for each device are summarized and averaged out in the revised Figure 4, as shown in Fig. R9. For each device, we have performed 50 current cycles, which are defined by a high current density, and then 150 current pulses with a low current density. We count the skyrmion bag N in 50 cycles. The probability is defined as $P = N/50 \times 100\%$, which means the probability of creating the skyrmion bag during each current cycle. The bag creation probability in all devices follows similar trends as a function of Q.

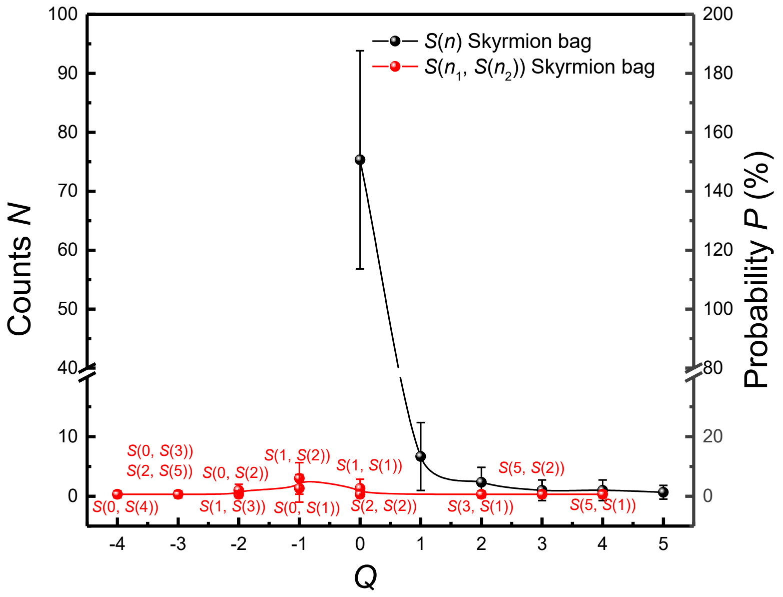

Fig. R9 (*i.e.*, Fig. 4a in the main text) **a** Counts of magnetic skyrmion bags, N, observed over 50 independent cycles, during the transition from a disordered helical state to a

skyrmion lattice. The generation probability is calculated as $p = N/50\times100\%$, representing the likelihood of skyrmion bag formation per current cycle. Each current cycle consists of a single high-current-density pulse followed by 150 low-current-density pulses. The statistical data were obtained from three devices with similar dimensions: length ~4 μm, width ~3 μm, and thickness ~100 nm. Error bars represent the standard deviation across 3 devices.

We have added the reproducibility in multiple devices in our revised manuscript, see Lines 268-271: "*The reproducibility of current-induced skyrmion bag creation was tested in multiple microdevices with thicknesses ranging from 80 to 150 nm. Consistent current-induced dynamics were observed across all devices (Supplementary Fig. 13–16).*"

Comment 4: Some additional queries from Figure 4. What is the area of the sample from which the number of high-Q skyrmions was counted? What is the number of normal skyrmions (Q=1, -1)? The authors did not mention about the size of the skyrmions. The nested skyrmions have significantly high energy. So, why do they form? An explanation about the probability calculation is needed.

Response: We thank you very much for your careful reading. We count the high-Q skyrmions in the entire area of devices. The size of skyrmions is 70 nm for FeGe magnets, consistent with previous FeGe studies (e.g., Yu *et al.* Nat. Mater. 10: 106 (2011)). We have added a new figure to show the number of normal skyrmions as a function of pulse count, as shown in Fig. R10.

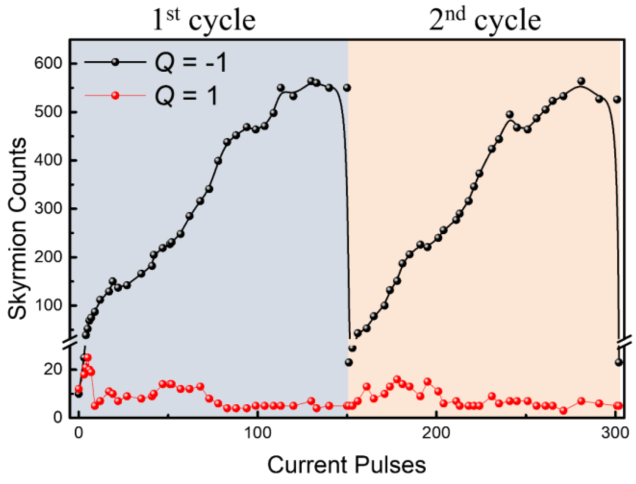

Fig. R10 (*i.e.*, Supplementary Fig. 12) Count of $Q = 1$ and -1 skyrmions during each current cycle. Each current cycle consists of a single high-current pulse with a density of 5.5×10^{10} A/m², followed by 150 low-current pulses with a density of 4.5×10^{10} A/m². The pulse duration is set to 70 ns with a frequency of 1 Hz.

We have added the skyrmion size discussion in the revised manuscript, see Line 97: "*In the B_{20} FeGe magnets, the skyrmion size is about 70 nm*[19,32]."

We have added the skyrmion count discussion in our revised manuscript, see Lines 237-242: "*Each current cycle consists of a single high-current pulse with a density of 5.5* $\times 10^{10}$ *A/m², followed by 150 low-current pulses with a density of 4.5* $\times 10^{10}$ *A/m². Initially, a few skyrmions with topological charges* $Q = 1$ *and* $Q = -1$ *are present. Upon application of positive current pulses,* $Q = -1$ *skyrmions are generated, whereas* $Q = 1$ *skyrmions either gradually annihilate or evolve into skyrmion bags (Supplementary Fig. 12).*"

The skyrmion bags can form because of the coexistence of skyrmions with $Q = 1$ and -1 at zero magnetic fields. The skyrmion bags survive as a high-energy metastable phase. Initially, both skyrmions with $Q = 1$ and -1 are observed. Upon the pulse is on, skyrmions with $Q = -1$ gradually increase to form skyrmion clusters/lattices. Skyrmions with $Q = 1$ are then embedded in the skyrmion lattices with $Q = -1$. Skyrmion lattices with $Q = -1$ possess background magnetization along the $+z$ axis; in contrast, the outer magnetization of skyrmions with $Q = 1$ is along the $-z$ axis. Thus, to keep the uniform for outer magnetization, skyrmions with $Q = 1$ need to be encircled by a skyrmion with $Q = -1$, resulting in the formation of skyrmion bags.

We have added the skyrmion number and stability of the skyrmion bag discussion in the revised manuscript, see Lines 254-258: "*The skyrmion bags stabilize as high-energy metastable phases, originating from initial skyrmion seeds with different signs of Q contrast to that of the skyrmion lattice. To keep the outer magnetization consistency, the initial skyrmions with* $Q = 1$ *must be encircled by a skyrmion with* $Q = -1$*, resulting in the formation of skyrmion bags*."

Comment 5: Why did the authors choose FeGe material for this study? Also, why was 100 nm thickness of FeGe chosen? I was wondering whether the material parameters, measurement temperature, and sample thickness have crucial roles in the formation of high-Q skyrmions, i.e., the number of high-Q skyrmions.

Response: Thank you for your thoughtful comments. Below, we provide a detailed response.

Choice of FeGe and thickness selection. We chose FeGe for this study because high-Q skyrmions have been shown to exhibit good stability in FeGe (Tang J. et al. *Nat. Nanotechnol.* **16**, 1086 (2021)). However, the creation mechanism for skyrmion bags—namely, the coexistence of $Q = 1$ and $Q = -1$ skyrmions at zero magnetic field and the STT-driven splitting of helices—is not specific to FeGe. In principle, our experimental observations can be extended to other skyrmion-hosting materials. We have clarified this point in the revised manuscript (Lines 227–233): "*The creation mechanism of skyrmion bags relies on the coexistence of skyrmions with opposite topological charges at zero magnetic field, and the STT-driven instability of helix ends—both of which are rooted in topology and dynamics rather than in specific material parameters. Therefore, our experimental findings can, in principle, be generalized to other skyrmion hosting*

systems, including room temperature chiral magnets and multilayers with interfacial DMI[14]."

Regarding thickness, we selected 100 nm primarily because it is compatible with transmission electron microscopy (TEM) imaging, which typically requires sample thicknesses below 150 nm. Following your suggestion, we systematically investigated the influence of sample thickness on skyrmion bag formation. Our experiments show that skyrmion bags can be reliably created in devices with thicknesses ranging from 80 to 150 nm (Supplementary Fig. 13–16). For thinner devices (e.g., below 80 nm), the in-plane shape anisotropy becomes dominant and suppresses the stabilization of skyrmions (Fig. R11).

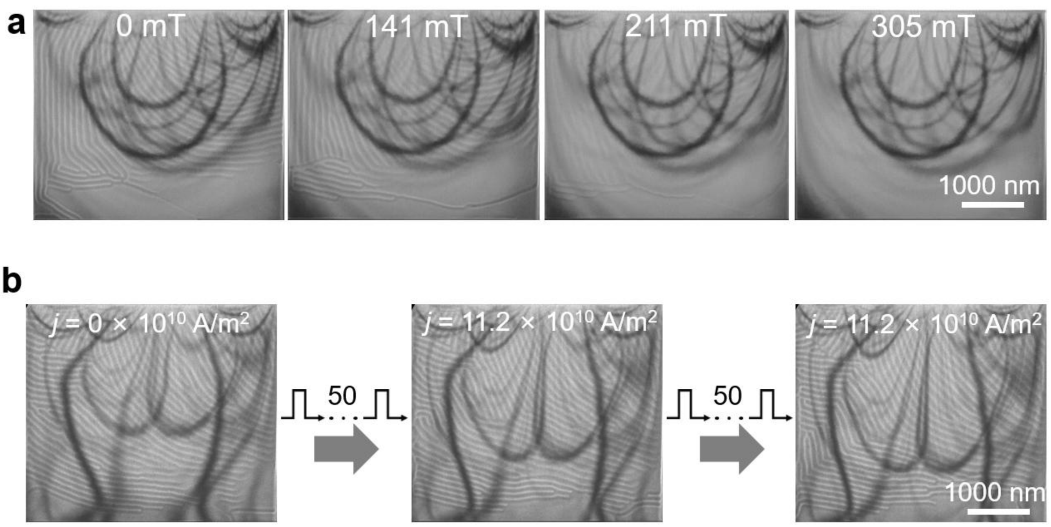

Fig. R11 Magnetic evolution in a thinner 70-nm FeGe lamella. **a** Magnetic field dependence of the magnetic evolution during the field-increasing process. **b** Magnetic domain states after applying 100 current pulses of 20 ns duration. No skyrmions are observed.

Role of temperature. We also examined the role of temperature, using the $S(1)$ bag as a representative example. For a fixed pulse width, we first determined the optimal current density j_{opt} for skyrmion bag creation at each temperature (Supplementary Fig. 2). At each temperature, we then applied 50 current cycles at j_{opt} and counted the number of $S(1)$ bags formed (Supplementary Fig. 17b). Our results show that the number of skyrmion bags decreases with increasing temperature, indicating that skyrmion bags—being high-energy metastable states—are more readily created at lower temperatures. At elevated temperatures, thermal fluctuations provide an additional driving force that promotes the transformation from skyrmion bags to isolated skyrmions.

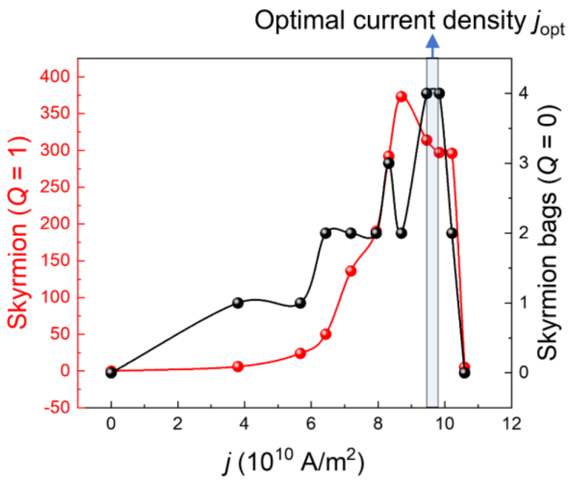

Fig. R12 (*i.e.*, Supplementary Fig. 2) The maximum skyrmion and $Q = 0$ skyrmion bag counts as a function of current density induced by current during each current cycle.

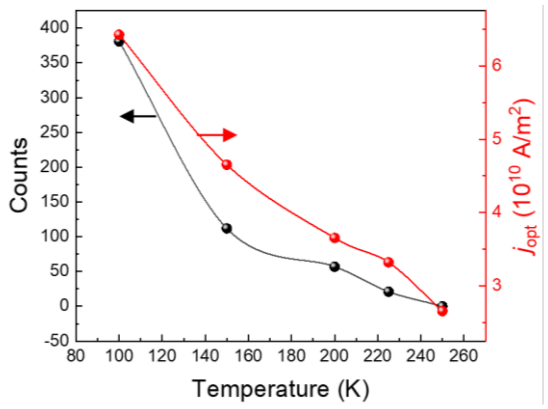

Fig. R13 (*i.e.*, Supplementary Fig. 17b) The $S(1)$ skyrmion bag counts emerging in 50 current cycles as a function of temperature. The current during the cycle is chosen as the optimal current density. The pulse width is 20 ns.

These experimental findings are summarized in Supplementary Fig. 17b and have been incorporated into the revised manuscript (Lines 268–274): "*The reproducibility of current-induced skyrmion bag creation was tested in multiple microdevices with thicknesses ranging from 80 to 150 nm. Consistent current-induced dynamics were observed across all devices (Supplementary Fig. 13–16). Owing to their high-energy metastable nature, thermal fluctuations promote the transformation from skyrmion bags to isolated skyrmions. Accordingly, our experiments show that the probability of skyrmion bag formation per current cycle decreases with increasing temperature (Supplementary Fig. 17b).*"

Comment 6: How was the pulse width of the current decided? Is there any effect of pulse width on the formation of skyrmion bags?

Response: The effect of pulse width on skyrmion bag formation was systematically examined, as presented in Fig. R14. Over 50 current cycles, the total count of skyrmion bags decreases monotonically as the pulse width is increased from 20 ns to 160 ns. We attribute this behavior to the metastable nature of skyrmion bags. At the same temperature, under longer current stimuli, there is a higher probability that skyrmion bags will transform into isolated skyrmions, driven by thermal fluctuations and the continuous application of spin-transfer torque.

We have added a discussion on pulse width in our revised manuscript, see Lines 274-278: "*In contrast, the probability of skyrmion bag formation per current cycle decreases with increasing pulse width (Supplementary Figure 17a). Under longer current stimuli, there is a higher probability that skyrmion bags will transform into isolated skyrmions, driven by thermal fluctuations and the continuous application of spin-transfer torque.*"

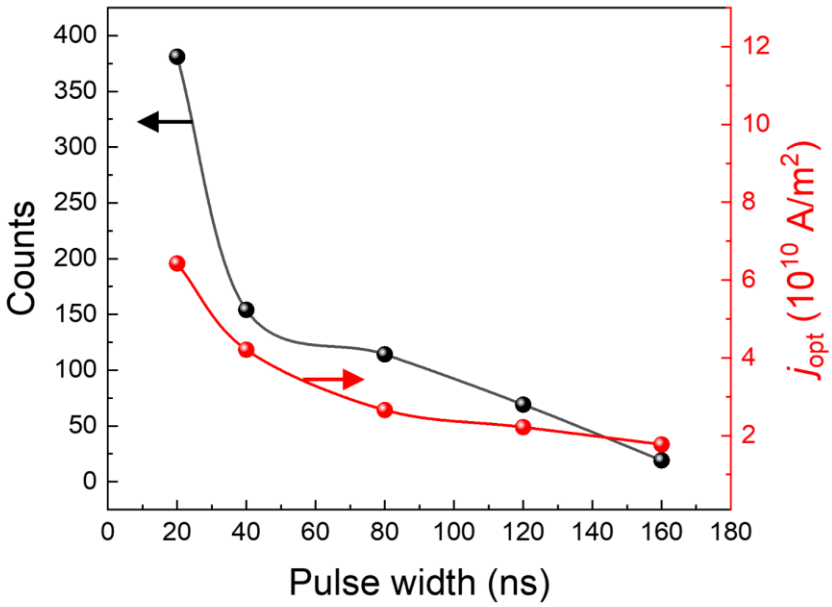

Fig. R14 (*i.e.*, Supplementary Fig. 17a) The *S*(1) skyrmion bag counts emerging in 50 current cycles as a function of pulse width. The current during the cycle is chosen as the optimal current density.

Comment 7: On page 9, the authors commented that "In our present experiments, the skyrmion bag is stabilized within a skyrmion lattice". How did the authors confirm that the skyrmion bags are stabilized over skyrmion bundles in the experiment?

Response: We thank you very much for your careful reading. In our manuscript, we term these high-*Q* spin textures as skyrmion bags from the contrast difference between internal skyrmions and outer boundary spirals. For skyrmion bundles, the outer boundary spirals show very weak integral in-plane magnetization due to the depth-modulated spin twists (Fig. R15b). In contrast, the high-*Q* magnetic soliton in this work

reveals a similar bag configuration across all the layers, resulting in similar contrast intensity between the internal skyrmions and boundary spirals (Fig. R15a).

The difference in experiment distinguishes the skyrmion bundles and skyrmion bags, which are discussed in our manuscript, see Lines 174-179: "*For skyrmion bundles characterized by a depth-modulated spin twisting*[19,21]*, the average in-plane magnetizations of the boundary spirals are significantly weaker compared to those of the interior skyrmions. However, for skyrmion bags, the retrieved average in-plane magnetizations of the boundary spirals are comparable to those of the interior skyrmions.*"

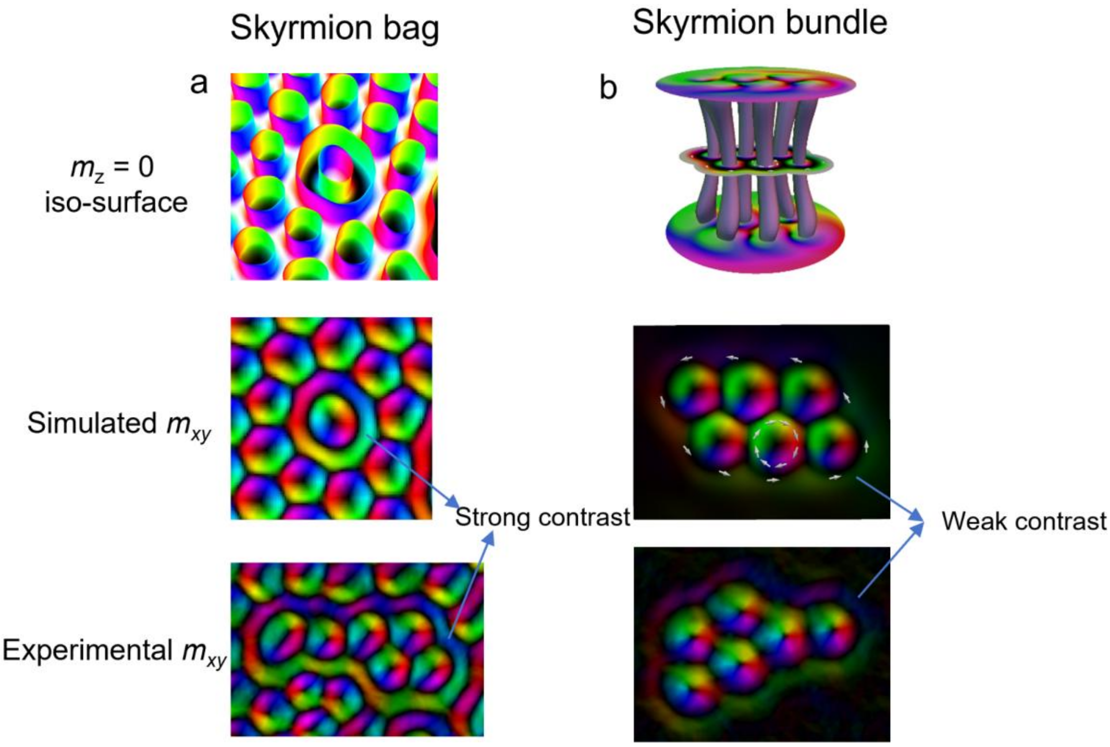

Fig. R15 Comparison of 3D skyrmion bags and skyrmion bundles. Simulated $m_z = 0$ iso-surface, simulated in-plane magnetizations m_{xy}, and experimental in-plane magnetizations m_{xy} of skyrmion bags (a) and skyrmion bundles (b).

Comment 8: The authors explained that the high-Q skyrmions are transformed into lower-Q skyrmions after the application of current pulses. If it is true, how are the high-Q skyrmions actually formed, after how many pulses? What are the effects of current density, polarity, and pulse width in this case? In the manuscript, the given explanations are quite confusing.

Response: Following your nice suggestion, we have added effects of current density, pulse width, and polarity on the topological transformations, as shown in Fig. R16-R18. Once the high-*Q* skyrmions are formed during the application of current pulses, they keep stable when the current pulse is off. During the application of current, the current-

induced Joule thermal heating plays an important role in determining its transformation to low-Q bags. For longer current density and longer current pulse width, the Joule thermal heating effect becomes more remarkable. For a fixed current pulse duration, our results show that the $-S(2)$ bag becomes more easily transformed into low-Q bags with the increase in the current density. In contrast, for a fixed current density, the $-S(2)$ bag becomes more easily transformed into low-Q bags with the increase in the pulse duration.

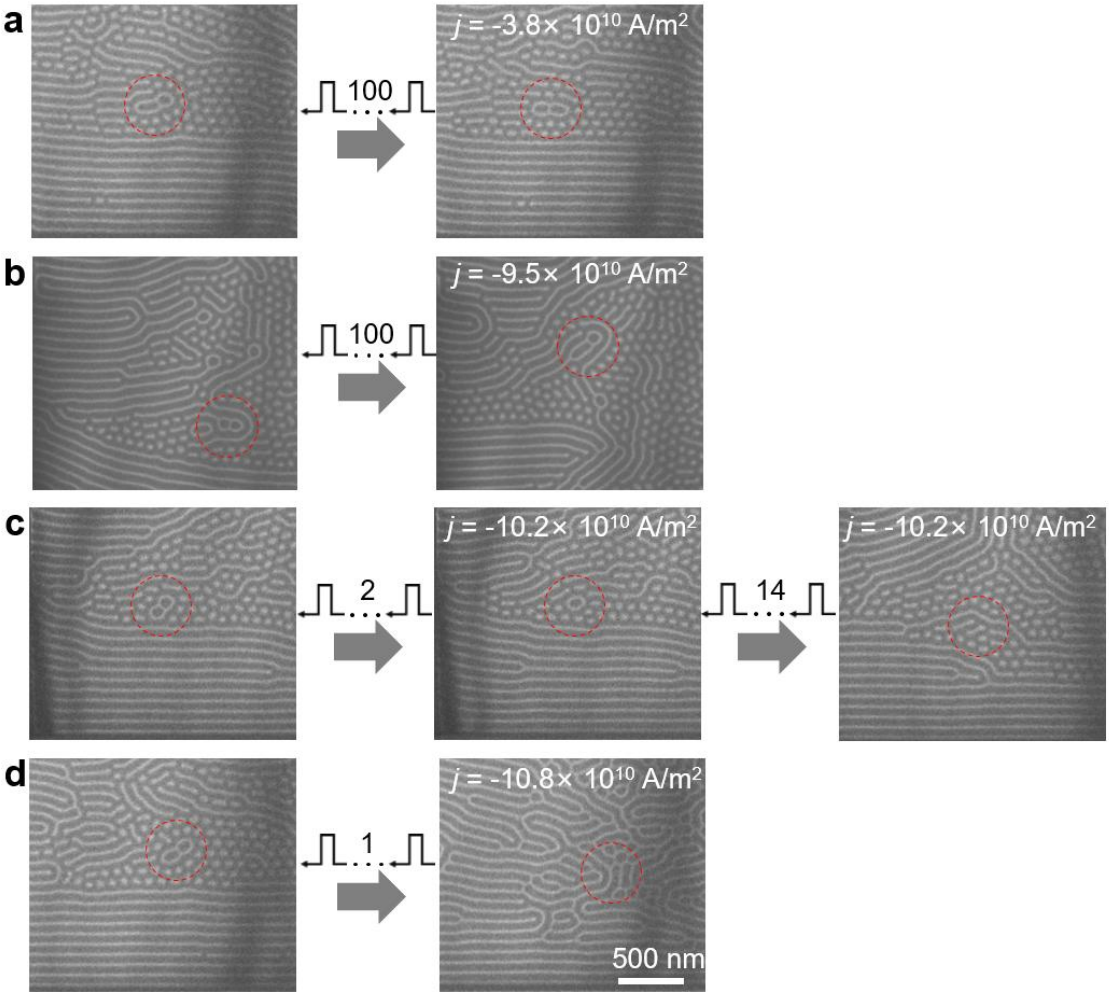

Fig. R16 (*i.e.*, Supplementary Fig. 19) Effect of current density on the stability of the $-S(2)$ skyrmion bag for a fixed pulse width of 20 ns. The $-S(2)$ skyrmion bag retains its topological state after applying 100 current pulses with densities of -3.8×10^{10} A/m² (a) and -9.5×10^{10} A/m² (b). At a current density of -10.2×10^{10} A/m² (c), the bag first transforms into a $-S(1)$ bag after 2 pulses and subsequently into a $Q = 1$ skyrmion after 14 pulses. At a higher current density of -10.8×10^{10} A/m² (d), the state is reset after a single pulse.

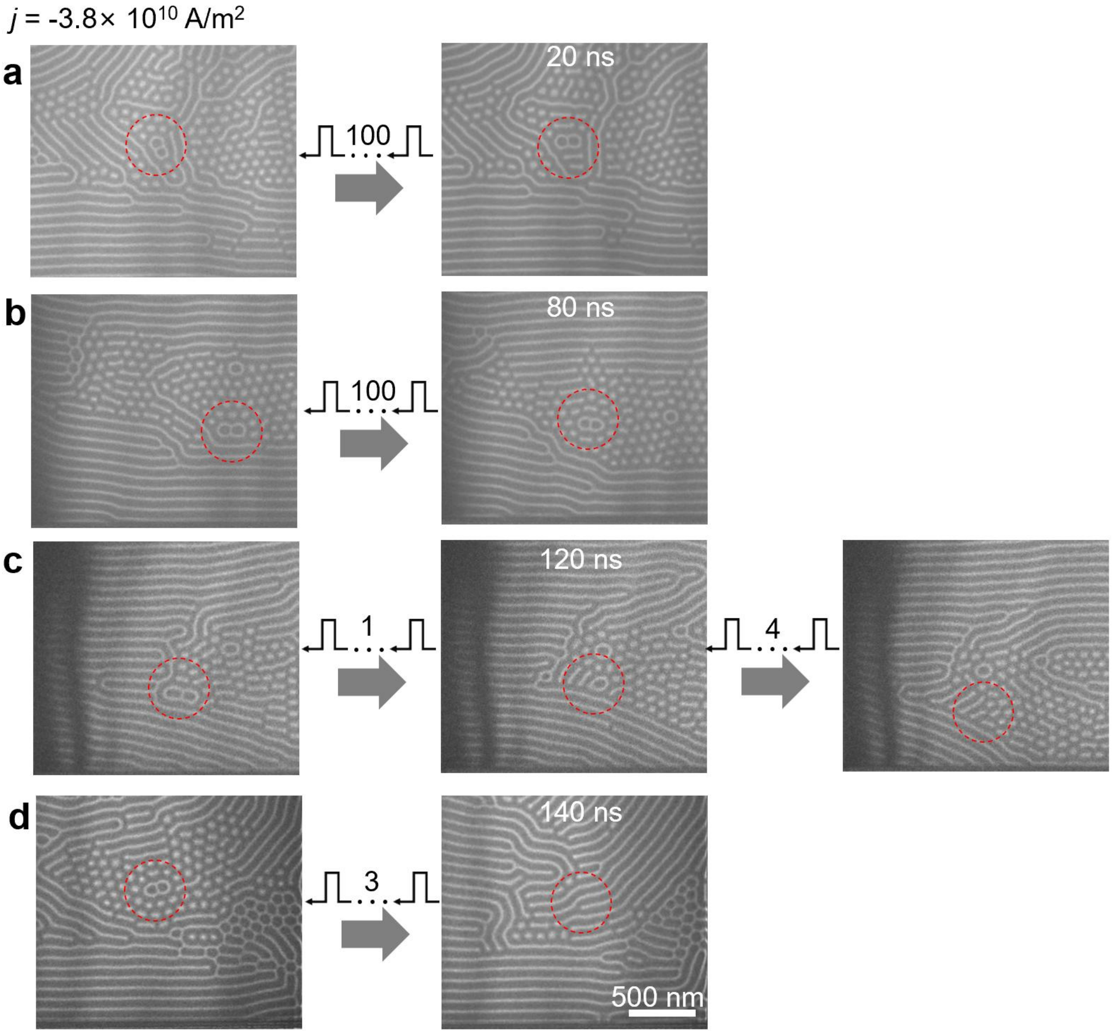

Fig. R17 (*i.e.*, Supplementary Fig. 20) Effect of pulse width on the stability of the $-S(2)$ skyrmion bag for a fixed current density of -3.8×10^{10} A/m^2. The $-S(2)$ skyrmion bag retains its topological state after application of 100 current pulses with a width of 20 ns (a) and 80 ns (b). At a width of 120 ns (c), the $-S(2)$ skyrmion bag first transforms to a $-S(1)$ bag after 1 current pulse and finally $Q = 1$ skyrmion after 4 current pulses. At a width of 140 ns (d), the state is quickly reset after 3 current pulses.

For the effect of current polarity. When the current orientation is reversed, the polarity and the charge sign of current-induced skyrmions are both reversed, as shown in Fig. R18. Based on the symmetry analysis and selective polarity rule (Fig. R3), the initial skyrmion bags annihilate during the background magnetization transitions between $Q = -1$ skyrmion lattice and $Q = 1$ skyrmion lattice (Fig. R18).

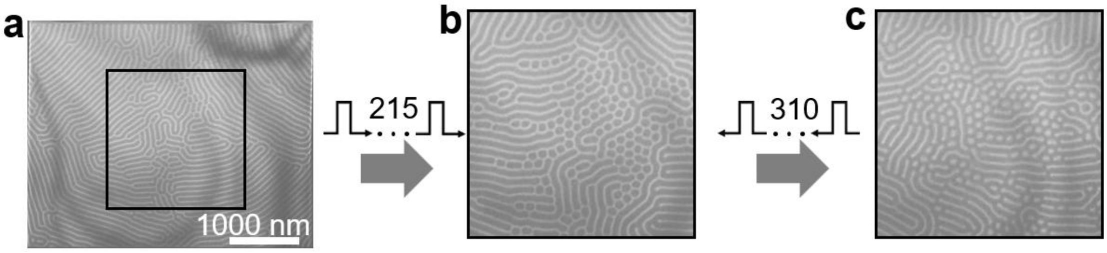

Fig. R18 (*i.e.*, Supplementary Fig. 7) Skyrmion polarity reversal by reversing the current orientation. **a** Initial domain states. **b** Current-induced skyrmions and skyrmion bags after applying 215 current pulses with a density of $+8.4\times10^{10}$ A m^{-2}. **c** Subsequent state after applying 310 current pulses with a density of -8.4×10^{10} A m^{-2}. The Fresnel images in (b) and (c) correspond to the region indicated by the black box in (a). Defocus distance: −500 μm.

We have added effects of current parameters on topological transformations in our revised manuscript, see Lines 293-300: "*The topological transformations from S(n) to S(n − 1) bags are understood by the instability of skyrmion bags due to the current-induced Joule thermal heating. For longer current density and longer current pulse width, the Joule thermal heating effect becomes more remarkable, causing the easy transformations (Supplementary Fig. 19 and 20). If the current orientation is reversed, based on the symmetry analysis and selective polarity rule (Supplementary Fig. 10), the initial skyrmion bags annihilate during the background magnetization transitions between $Q = -1$ skyrmion lattice and $Q = 1$ skyrmion lattice (Supplementary Fig. 7).*

Comment 9: On page 6, the authors mentioned "Once current is applied, the spin-transfer torque forces the formation of a horizontal helix with a q-vector perpendicular to the current orientation, as experimentally identified (Fig. 2b). However, we can always observe a perpendicular helix whose top ends are pinned from the geometrical edges". What is the meaning of "q-vector" here? Authors are requested to clarify these statements. Also, the formation process of skyrmions from perpendicular helix is not well explained in the manuscript. On the next page, the authors mentioned "The long helix could undergo the integral crash forces of external individual skyrmion moving to the bottom ends, contributing to the formation of individual S(n) bags". This statement is also not very clear to me.

Response: Response to clarification of "*q*-vector". We agree that the term "*q*-vector" requires explicit definition. In chiral magnets such as FeGe, the competition between the ferromagnetic exchange interaction and the Dzyaloshinskii–Moriya interaction (DMI) stabilizes a helical spin modulation. This modulation is characterized by a propagation direction and a periodicity, formally described by a wave vector q. The q-vector defines both the orientation of the spin spiral and its wavelength. Specifically, for a helical phase, the magnetization rotates in a plane perpendicular to q, while for a conical phase, it rotates with a canting angle relative to q. This concept is standard in the literature (see, e.g., Schoenherr *et al.*, Nat. Phys. 14, 465 (2018); Kim *et al.*, Phys. Rev. Lett. 134, 136704 (2025)). We have now included this definition in the revised manuscript and added a schematic illustration (Fig. R19, which corresponds to Fig. 2 in the main text) to aid reader understanding.

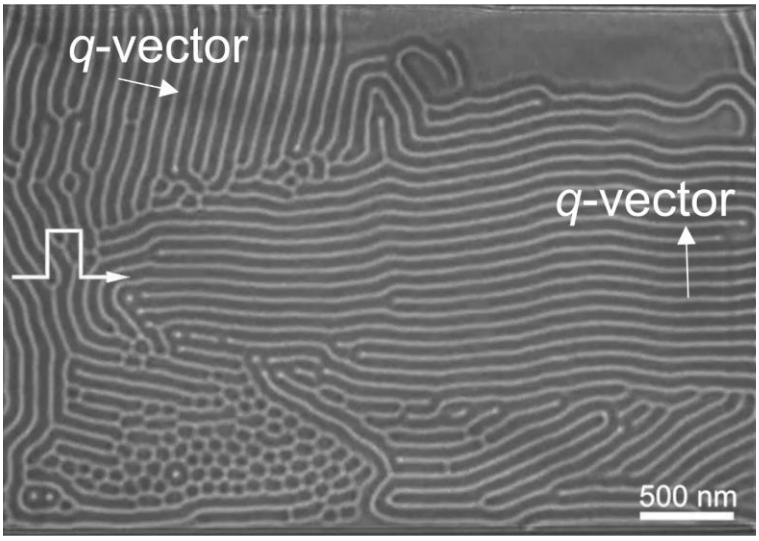

Fig. R19 (*i.e.*, Fig. 2 in the main text) Definition of *q*-vector.

Response to Formation of skyrmions from a perpendicular helix. We acknowledge that the original description of skyrmion formation from a perpendicular helix was insufficiently detailed. The process is governed by the skyrmion Hall effect, which arises from the non-zero topological charge Q of magnetic solitons. As shown in Fig. R3, the ends of a long vertical helix are topologically equivalent to merons with $Q = \pm 1/2$. When an in-plane current is applied, the spin-transfer torque exerts a force on these topological entities, causing them to move transversely to the current direction—a hallmark of the skyrmion Hall effect. The direction of this transverse motion is determined by the sign of Q.

In our geometry, one end of the vertical helix is pinned at the device edge (e.g., the bottom side). If the opposite (free) end experiences a Hall shift away from the pinned side, the helix becomes stretched. When the tensile stress exceeds a threshold, the helix breaks, nucleating an isolated skyrmion. This mechanism is illustrated schematically in Fig. R3 (new figure in the revised manuscript). Based on symmetry, we identify four possible scenarios depending on the pinning location and current orientation. Importantly, our experimental observations (Fig. R4) are fully consistent with this picture and exhibit the predicted correspondence between pinning side and current direction. This consistency also serves to exclude alternative mechanisms such as the Oersted field or Joule heating, which do not exhibit such directional dependence.

We have revised the manuscript to include this detailed explanation, supported by new schematic figures and experimental data.

Response to the formation of skyrmion bags. The statement regarding the formation of skyrmion bags has also been clarified. The process begins with a long vertical helix that contains an embedded skyrmion of opposite topological charge (e.g., $Q = -1$) relative to the surrounding skyrmion cluster/lattice (e.g., $Q = +1$). Under the application of current, the skyrmions in the surrounding lattice experience a transverse Hall motion. These moving skyrmions can impinge upon and destabilize the long helix, eventually

causing it to rupture. The result is the formation of a skyrmion bag—a composite object consisting of inner skyrmions (with the original opposite charge) enclosed by a spiral boundary. This mechanism is now described in the revised text and is accompanied by a schematic in Fig. R20.

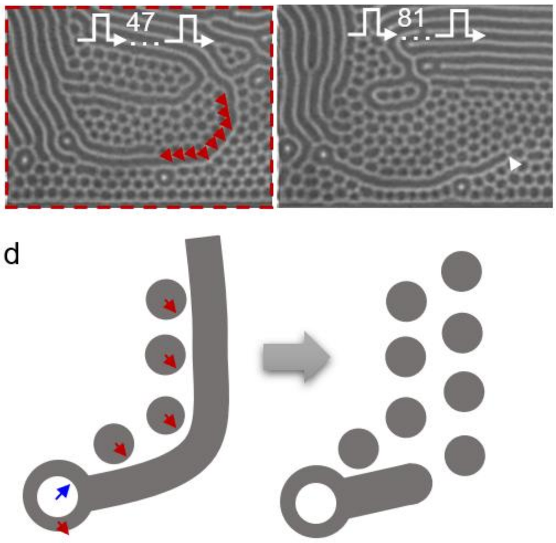

Fig. R20 (i.e., Fig. 2 in the main text) Schematic formation of *S*(n) bags because of the split of helix during the rush of moving individual skyrmions. The red arrows indicate the moving direction of skyrmions under STT because of skyrmion Hall effects.

We have added the definition of *q*-vector in our revised manuscript, see Lines 119-122: "*In chiral magnets, the competition between exchange and Dzyaloshinskii-Moriya interaction (DMI) forces the magnetizations to twist periodically on a plane either perpendicularly (helix) or with a canting angle (cone) along a specific direction, defined as q-vector* [35,36]."

We have added the skyrmion Hall shift effects on the current-induced creation of skyrmions and skyrmion bags, see Lines 140-142: "*Because of skyrmion Hall effects driven by current, the long helix could undergo the integral crash forces of external skyrmion clusters moving to the bottom ends, contributing to the formation of individual S(n) bags (Fig. 2d).*"

Comment 10: Some more comments:
(i) What is the sign convention of the applied current pulses?

Response: The sign convention for the applied current pulses has now been explicitly stated in the revised manuscript (see Lines 106–108): "*We define a positive current pulse as one in which the current flows from the left to the right side of the device, and a negative current pulse as one in which the current flows from the right to the left side.*"

(ii) On page 6, the authors mentioned that "Notably, the outermost magnetizations of both S(n) and S(n1, S(n2)) bags exhibit an out-of-plane up orientation, as shown in Fig. 1c". Should it be "out-of-plane down orientation"?

Response: Based on the sign convention and color coding defined in our manuscript (e.g., in Fig. 1 and the accompanying description), the outermost magnetizations of both *S*(n) and *S*(n1, *S*(n2)) bags indeed exhibit an out-of-plane up orientation. In our convention, white corresponds to magnetization pointing upward (positive out-of-plane component), while black corresponds to downward orientation. As shown in Fig. R21, the background and the bag boundaries consistently appear in red, indicating an upward magnetization direction.

To avoid any ambiguity, we have verified that the color mapping and orientation definitions are clearly stated in the figure caption and the main text, see Lines 92-94: "*The color coding indicates the magnetization orientation as defined in the colorbar: white represents an out-of-plane upward magnetization, while black represents an out-of-plane downward magnetization.*"

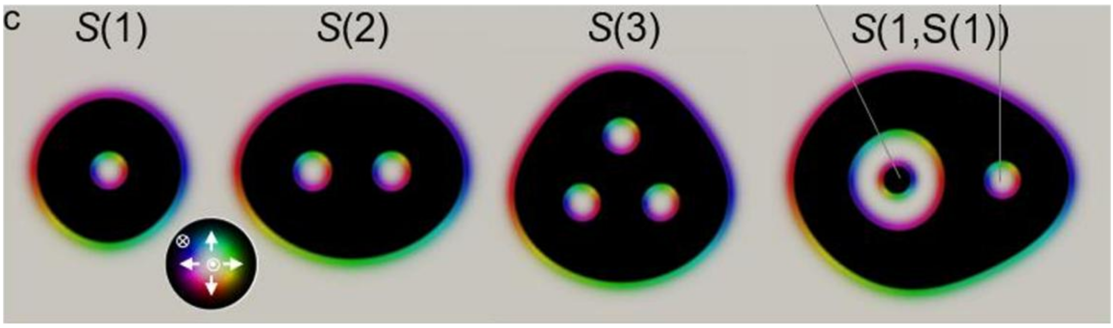

Fig. R21 (i.e., Fig. 1 in the main text) Representative examples of S(n) and S(n1, S(n2)) skyrmion bags. The color coding indicates the magnetization orientation as defined in the colorbar: white represents an out-of-plane upward magnetization, while black represents an out-of-plane downward magnetization.

(iii) How long do the high-Q skyrmions remain stable after formation?There are multiple, mostly minor, deficiencies that I believe need to be addressed before the manuscript receives further consideration.

Response: Following your comment, we have investigated the stability of skyrmion bags after their formation. Under constant physical conditions (i.e., without changing temperature, magnetic field, or applied current), we monitored the skyrmion bags over time using repeated imaging. Our experiments reveal that the skyrmion bags exhibit no observable variation in their morphology, size, or position for periods exceeding 13 hours. This indicates that these high-*Q* spin textures are remarkably stable at zero external magnetic field once formed.

We have added this important finding to the revised manuscript (see Lines 278–283): "*To assess the stability of skyrmion bags, we monitored the magnetic texture under constant conditions (zero applied current, 95 K, and zero external magnetic field) for over 13 hours (Supplementary Fig. 18). Repeated imaging reveals no detectable changes in the morphology, size, or position of the skyrmion bags, demonstrating their long-term stability.*"

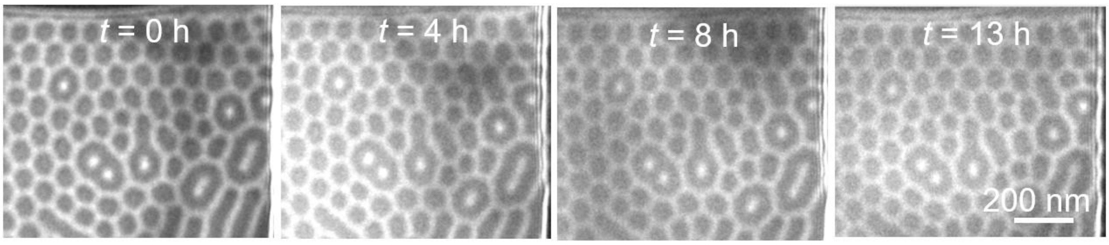

Fig. R22 (*i.e.*, Supplementary Fig. 18) Skyrmion bag stability over time under constant conditions (zero applied current, 95 K, and zero external magnetic field).

We are greatly grateful to you for the thorough reading of our manuscript and for identifying multiple points that required clarification. We believe that these revisions have substantially improved the quality and clarity of our work. We thank you very much again for the valuable feedback and hope that the revised manuscript is now suitable for publication in *Nature Communications*.

Response to Reviewer #2:

Reviewer #2: The manuscript reports the observation of skyrmion bags that spontaneously emerge during the current-induced transformation from the chiral labyrinth domain state (called by the authors a spin spiral) into the skyrmion lattice. In essence, this effect stems from the formation of closed domains whose direction is inverted compared to that favored by the combination of dominant domain termination and current direction. The closed domain traps inverted skyrmions inside. The authors found that a varied number of skyrmions can be trapped, and observed current-driven transformations among different skyrmion populations in the bag.

This work is part of a series of papers published by the same authors on the subject of current-induced skyrmion generation. Notably, Nano Letters, 22, (2022) cited as Ref. 35 in the manuscript showed current-induced generation of skyrmions in the same material (FeGe) and the same structure, also at zero magnetic field. Ref. 25 Nano Letters, 25 (2025) demonstrated stable skyrmion bags in FeGaTe defined by current sequence with assistance from the field.

The main claim of novelty in the present manuscript is that skyrmion bags are generated by current without assistance from the field. I find the presented results clear, convincing, aesthetically pleasing, easy to popularize for the general public. They advance the field of skyrmionics. Thus, I expect that this work will be cited and will have an impact both on the field and on the broader scientific community.

Response: We are truly grateful for your thoughtful and encouraging assessment of our work. Your positive evaluation—highlighting that the results are "*clear, convincing, aesthetically pleasing, and advance the field of skyrmionics*"—means a great deal to us. We are delighted that you recognize the potential impact of our findings on both the specialized field and the broader scientific community.

On the technical level, my main concern is that the domain termination and direction relative to current determines the type of current-generated skyrmions. Consequently, generation of skyrmion bags starting from a random magnetic configuration without magnetic field assistance is an uncontrolled process. In my opinion, this randomness diminishes the practical significance of the presented results, under the assumption that skyrmionic devices can, in principle, become practical.

From the more fundamental point of view, I feel that the manuscript is a relatively modest advancement compared to the results previously published by the group. This is evidenced by the fact that the manuscript does not provide a new theoretical analysis. Therefore, I am overall not convinced that the level of novelty or practical significance warrant publication in Nat. Comm.

Response: We appreciate your acknowledgment of the technical quality of our work and your recognition of its aesthetic appeal and potential impact. We also value your critical perspective on the practical significance and novelty, which allows us to further clarify the contributions of our manuscript.

We agree with you that the current-induced generation of skyrmion bags starting from a random magnetic configuration without field assistance exhibits an inherent stochasticity. This is indeed an important consideration for future practical devices, where deterministic control is essential. However, we view our results as an essential first step toward that goal. By demonstrating that skyrmion bags can be created electrically at zero magnetic field—a capability not achieved before—we establish a foundational principle: pure current stimuli are sufficient to nucleate these high-Q textures. The formation process is governed by the relationship between domain termination and current direction. This very insight provides a potential pathway toward deterministic control: by artificially engineering the initial domain structure (e.g., through patterned pinning sites, designed nucleation seeds, or pre-conditioning current pulses), one could, in principle, achieve precise and repeatable skyrmion bag creation. We have now emphasized this outlook in the revised manuscript, see Lines 382–387: "*The stochastic nature of skyrmion bag formation in our zero-field electrical approach, while currently limiting deterministic operation, highlights the governing roles of local domain terminations and current direction. Achieving deterministic electrical creation of skyrmion bags will require further exploration, particularly through local manipulation techniques such as laser irradiation and lithographically defined pinning sites.*"

We respectfully offer a different perspective on the novelty of our findings. While our group has previously demonstrated current-induced skyrmion generation (Ref. 21) and field-assisted skyrmion bag formation (Ref. 25), the present manuscript reports the following distinct advances: 1. First demonstration of pure electrical creation of skyrmion bags at zero magnetic field. This moves beyond field-assisted methods and represents a new regime of control. 2. Experimental signature of topological defect-mediated transformations. Our direct imaging reveals the role of monopole-like defects in enabling these transformations, offering experimental evidence for theoretical predictions about topological transitions in magnetic systems.

Moreover, in our revised manuscript, we present current-induced rotational dynamics of skyrmion bags, as shown in Fig. R23. Previous studies of skyrmion lattice dynamics have primarily relied on reciprocal-space observations using neutron or X-ray scattering. We note that the mechanism and more details underlying lattice rotation are far beyond the scope of the present work. Instead, our results demonstrate that skyrmion bags embedded in the surrounding lattice can serve as lattice defects that follow the dynamics of skyrmion lattices, enabling direct real-space visualization of lattice dynamics. This finding suggests a pathway for device applications that exploit skyrmion lattice dynamics by detecting the motion of skyrmion bags.

We have added a discussion about the potential application of skyrmion bags in imaging the skyrmion lattice dynamics in our revised manuscript, see Lines 339-348: "*Previous studies have established emergent skyrmion lattice dynamics, including rotation driven by spin torques, thermal gradients, and field gradients*[47-49]*. Typically, such dynamics are inferred from shifting scattering peaks in reciprocal-space neutron scattering experiments*[49]*. Skyrmion bags—which can be regarded as topological defects within the lattice—offer a unique opportunity to directly image lattice dynamics in real space. In our observations, skyrmion bags undergo counter-clockwise rotation within the lattice, revealing the counter-clockwise rotation of the entire skyrmion lattice (Supplementary Fig. 23 and Movie V3). These findings suggest a pathway for device applications that exploit skyrmion lattice dynamics by detecting the motion of skyrmion bags (Supplementary Fig. 23d).* "

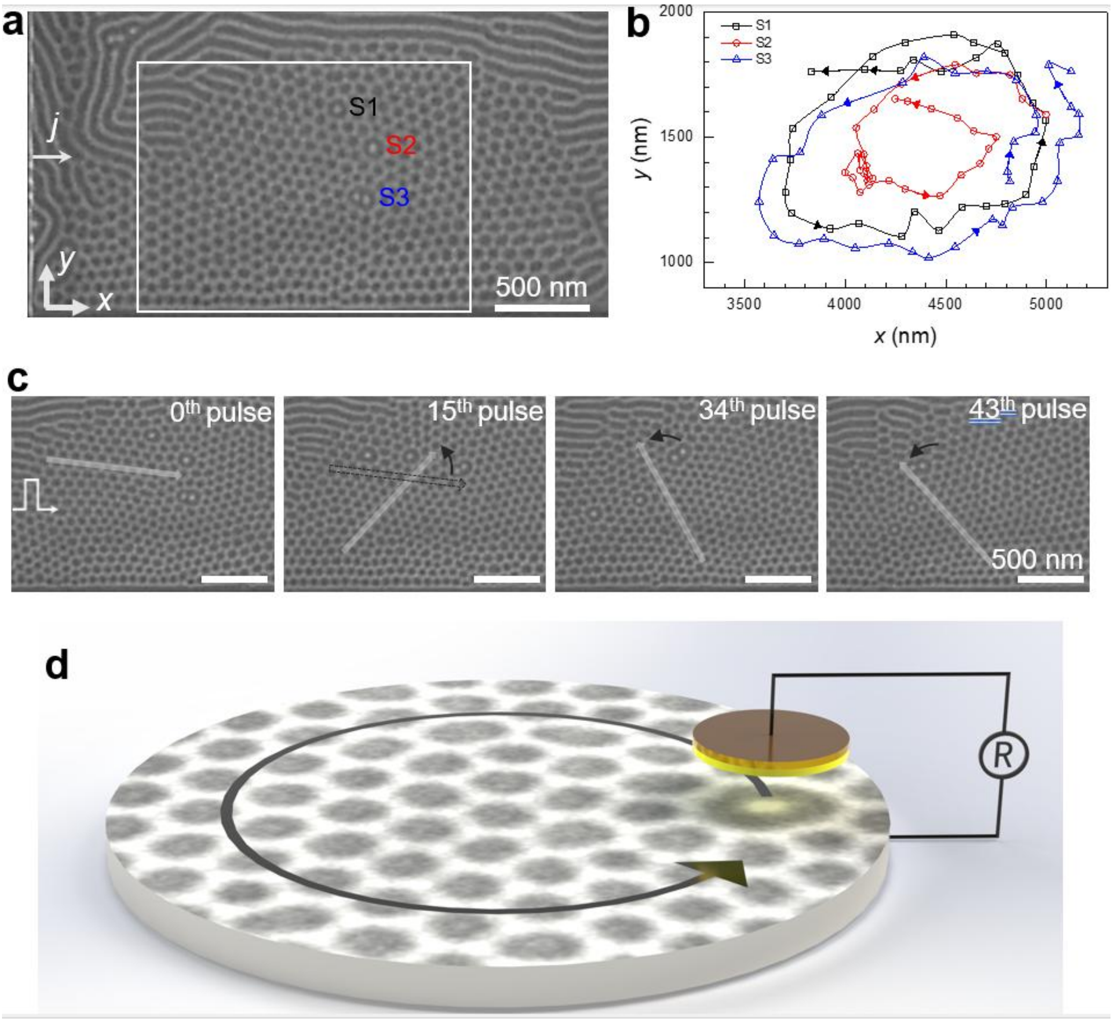

Fig. R23 (i.e., Supplementary Fig. 23) Current-driven rotation of skyrmion lattice and skyrmion bags at zero magnetic field. **a** The overall view of the device. **b** The bag trajectories during the application of current pulses. S1, S2, and S3 represent the skyrmion bag as marked in (a). **c** Snapshots of the lattice and bag rotation driven by nanosecond current pulses. **d** Schematic device design based on skyrmion lattice

rotation dynamics through the detection of embedded skyrmion bags. These Fresnel images were taken at a defocused distance of −500 μm.

We respectfully maintain that the electrically controlled creation, transformation, and rotation dynamics of skyrmion bags at zero magnetic field represent a meaningful advance beyond the current state of the art. We hope that, with the clarifications provided above, the reviewer may reconsider the manuscript's suitability for publication in *Nature Communications*.

We thank you again for the time and expertise invested in evaluating our work, and we remain open to further suggestions for improvement.

Response to Reviewer #3:

Reviewer #3: Referee Report on "Current-induced creation and dynamics of embedded magnetic skyrmion bags" by Yaodong Wu, Jialiang Jiang, Lingyao Kong, Meng Shi, Shouguo Wang, Mingliang Tian, Haifeng Du, and Jin Tang

This manuscript reports the current-induced creation and dynamics of magnetic skyrmion bags in FeGe nanoplates using in-situ Lorentz TEM. They performed well organized experiments and compared with the simulation.

I believe these results are, in principle, worthy of publishing on Nature Communications. While the realization of all-electrical control of high-Q topological textures is significant and timely, several critical points regarding the deterministic control, thermal effects, and 3D dynamics remain insufficiently addressed.

The following points should be clarified before publication.

Response: We sincerely appreciate your recognition of our results as "*worthy of publishing on Nature Communications*". Below, please find our response to your valuable comments.

Comment 1: -Stochasticity vs. Deterministic Control
The authors demonstrate that skyrmion bags can be generated via current pulses.
However, the process appears highly stochastic.
For potential applications in multi-state memory, deterministic switching between specific topological states (e.g., $S(n)$ to $S(n \pm 1)$) is essential.
The manuscript lacks a clear phase diagram or protocol that correlates specific pulse parameters (amplitude, duration, or frequency) with the resulting topological charge Q in a predictable manner.

Response: We agree with you that the current-induced generation of skyrmion bags starting from a random magnetic configuration without field assistance exhibits an inherent stochasticity. This is indeed an important consideration for future practical devices, where deterministic control is essential. We have performed systematic studies on the temperature, current density, and pulse duration on the current-induced creation of skyrmion bags. Our results reveal that the skyrmion bag can be more easily created at low temperatures and short pulse widths at an optimal current density (Fig. R24). The formation process is governed by the relationship between domain termination and current direction. This very insight provides a potential pathway toward deterministic control: by artificially engineering the initial domain structure (e.g., through patterned pinning sites, local pulsed lasers, or pre-conditioning current pulses), one could, in principle, achieve precise and repeatable skyrmion bag creation.

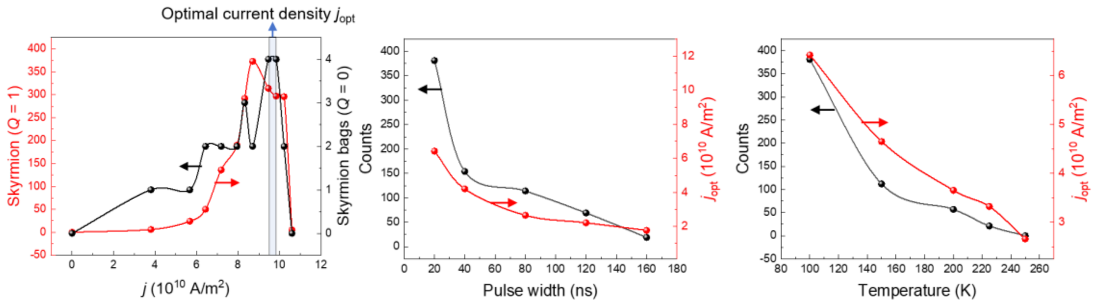

Fig. R24 (i.e., Supplementary Figures 2 and 17) Left: The maximum skyrmion and $Q = 0$ skyrmion bag counts as a function of current density induced by current during each current cycle. Middle: The $S(1)$ skyrmion bag counts emerging in 50 current cycles as a function of pulse width. The current during the cycle is chosen as the optimal current density. Right: skyrmion bag counts emerging in 50 current cycles as a function of temperature. The current during the cycle is chosen as the optimal current density. The pulse width is 20 ns.

However, we view our results as an essential first step toward that goal. By demonstrating that skyrmion bags can be created electrically at zero magnetic field—a capability not achieved before—we establish a foundational principle: pure current stimuli are sufficient to nucleate these high-Q textures.

We have now emphasized this outlook in the revised manuscript, see Lines 382-387: "*The stochastic nature of skyrmion bag formation in our zero-field electrical approach, while currently limiting deterministic operation, highlights the governing roles of local domain terminations and current direction. Achieving deterministic electrical creation of skyrmion bags will require further exploration, particularly through local manipulation techniques such as laser irradiation and lithographically defined pinning sites.*"

Comment 2: -Quantitative Separation of Joule Heating

The study attributes the bag formation primarily to Spin-Transfer Torque (STT). However, given the high current densities used ($j \approx 10^{10} \text{A/m}^2$), Joule heating is inevitable.
The authors should provide a more rigorous quantitative analysis or control experiment to distinguish the purely torque-driven dynamics from thermally-activated processes. Does the temperature rise during pulsing assist in overcoming the topological energy barriers?

Response: We thank you for this important question regarding the potential role of Joule heating. We agree that a quantitative separation of thermal and torque effects is essential, given the high current densities used in our experiments. Below, we provide additional simulations and control experiments to address this point.

<u>Quantitative evaluation of Joule heating.</u> We performed multiphysics field simulations to quantify the temperature rise induced by nanosecond current pulses under the same conditions as those used in Fig. 2 of the main text. The simulations consider the specific geometry, material properties (including electrical and thermal conductivities of FeGe), and pulse parameters. As shown in Fig. R25, the maximum temperature increase is approximately 14 K for a 70-ns current pulse with a density of 4×10^{10} A m^{-2}. This moderate temperature rise (≈14 K) is insufficient to drive topological transitions on its own, but it may assist the STT-driven splitting of skyrmions and skyrmion bags from the ends of the vertical helix by lowering the effective energy barrier.

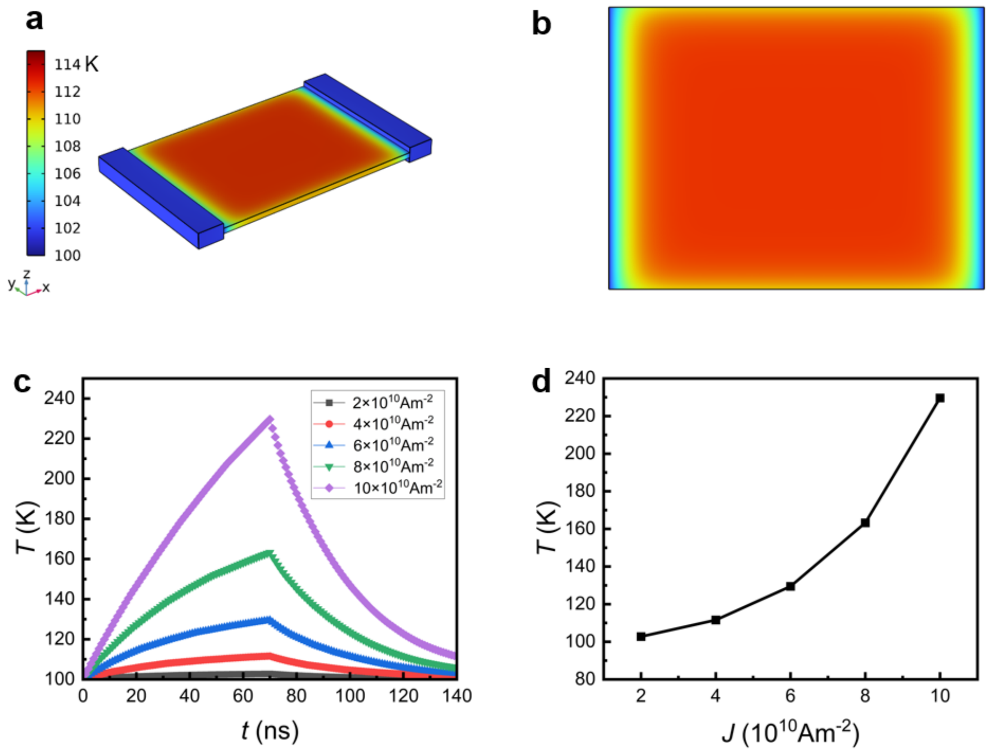

Fig. R25 (*i.e.*, Supplementary Fig. 8) Multiphysics field simulation of current-induced Joule thermal heating. **a** Simulated temperature distribution after a 70-ns current pulse with a current density of 4×10^{10} A m^{-2}. **b** Enlarged view of the temperature distribution in the device area. **c** Temperature at the center of the device as a function of time during the pulse for different current densities. **d** Maximum temperature rise versus current density for 70-ns pulses.

Joule heating depends only on the magnitude of the current density ($\propto j^2$), not on its sign. Therefore, if heating were the dominant mechanism, reversing the current polarity would not change the outcome. In contrast, STT is polarity-sensitive. Our experiments show that reversing the current direction reverses the topological charges of both the generated skyrmions and skyrmion bags (Fig. R26). This unequivocally demonstrates that STT—not Joule heating—determines the topological sign of the created textures.

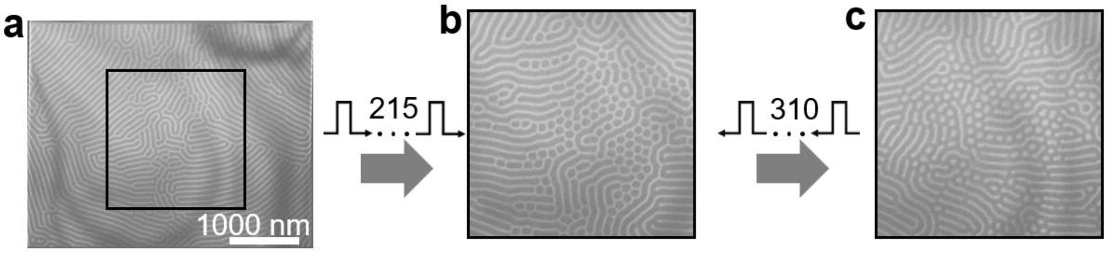

Fig. R26 (*i.e.*, Supplementary Fig. 7) Skyrmion polarity reversal by reversing the current orientation. **a** Initial domain states. **b** Current-induced skyrmions and skyrmion bags after applying 215 current pulses with a density of +8.4 A m^{-2}. **c** Subsequent state after applying 310 current pulses with a density of −8.4 A m^{-2}. The Fresnel images in (b) and (c) correspond to the region indicated by the black box in (a). Defocus distance: −500 μm.

Symmetry analysis. We further analyzed the relationship between helix pinning ends, current direction, and the topological charge of the resulting skyrmions/bags. For a vertical helix pinned at the top side, a positive current generates $Q = -1$ skyrmions and $S(n)$ bags, whereas a negative current generates $Q = +1$ skyrmions and $-S(n)$ bags. When the helix is pinned at the bottom side, the polarity dependence is reversed. These four predicted cases are systematically summarized in Fig. R27, and all are observed experimentally. This symmetry-based analysis provides strong evidence that the topological sign is governed by STT rather than by any current-direction-independent effect (such as Joule heating or Oersted fields).

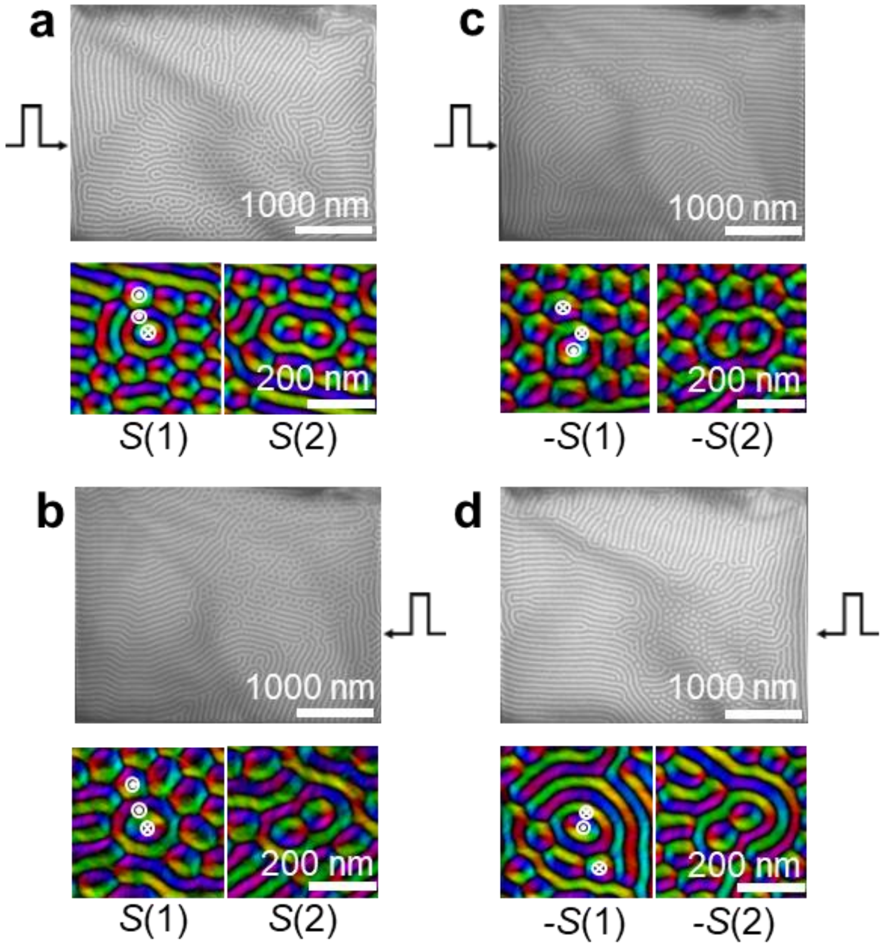

Fig. R27 (*i.e.*, Supplementary Fig. 11) Experimental demonstration of the skyrmion generation rule based on force and symmetry analysis. Fresnel images were taken at a defocus distance of −500 µm, revealing the pinning ends of the vertical helix. Representative in-plane magnetic configurations of skyrmion bags and skyrmions are shown for four cases, depending on the pinning location and current polarity: **a** vertical helix pinned at the top ends, positive current; **b** pinned at the bottom ends, positive current; **c** pinned at the bottom ends, negative current; **d** pinned at the top ends, negative current.

We have added a discussion regarding the Joule heating and torque contribution in our revised manuscript, see Lines 204-212: "*Current-induced effects also include Joule thermal heating and the Oersted field. By reversing the current orientation, the topological charges of current-induced skyrmions and skyrmion bags are reversed (Supplementary Fig. 7), ruling out Joule heating as the main origin, since it does not depend on current direction. Our multiphysic field simulation shows a maximum temperature rise of about 14 K for a 70-ns current pulse with a density of* 4×10^{10} *A* m^{-2} *(Supplementary Fig. 8). This moderate temperature rise may assist the splitting of skyrmions and skyrmion bags from the ends of a long vertical helix, but the topological sign is determined solely by STT.*" and Lines 219-224: "*Furthermore, symmetry and force analysis yield four distinct STT-driven dynamical behaviors depending on the*

pinning ends of the vertical helix and the current orientation (Supplementary Fig. 10). Our experiments reproduce all four predicted schemes(Supplementary Fig. 11). Consequently, we conclude that STT is the primary effect determining the topological sign of current-induced skyrmion bags and skyrmions."

Comment 3: -Ambiguity in 3D Topological Dynamics
The interpretation of the annihilation of internal skyrmions through magnetic monopoles or chiral bobbers relies heavily on micromagnetic simulations.
As Lorentz TEM provides only a 2D projection of the magnetization, the evidence for these 3D processes remains indirect.
The authors should discuss the limitations of their 2D observations in describing 3D tube-breaking events and provide more robust justification for their proposed 3D models.

Response: We thank you for this insightful comment. We fully acknowledge that Lorentz TEM provides only a 2D projection of the magnetization, and that direct evidence for 3D processes such as monopole-mediated annihilation or chiral bobber formation remains indirect.

Three-dimensional magnetic reconstruction typically requires a series of Lorentz TEM images acquired over a wide tilt angular range (e.g., from –60° to +60°). Unfortunately, this approach is not feasible with our current TEM instrument. However, while Lorentz TEM integrates the magnetization along the electron beam direction (*i.e.*, through the sample thickness), it can still differentiate certain 3D magnetic solitons from those with translational symmetry along the depth direction. Specifically, 3D depth-modulated spin textures exhibit weaker integrated contrast compared to depth-uniform skyrmion tubes, because the in-plane magnetization components partially cancel along the projection. This contrast-based distinction has been successfully used in previous experimental reports of 3D magnetic solitons, including chiral bobbers and skyrmion bundles (see, e.g., Refs. 7 and 19). In our work, we observe obvious contrast differences that are consistent with the presence of 3D depth-modulated structures.

To reflect this limitation more carefully, we have moderated our claim from a definitive identification to "*experimental signatures of 3D topological defects*." We have also added an outlook for future 3D imaging. The revised text (Lines 330–338) now reads:

"*The distinction between depth-uniform skyrmion tubes and depth-modulated 3D solitons (such as magnetic bobbers or skyrmion bundles) based on Lorentz TEM contrast differences has been established in previous studies* [7,16,19,34]. *Following this principle, our observed contrast features are consistent with the presence of 3D topological structures, but we refer to them as experimental signatures rather than definitive proof. For direct and unambiguous reconstruction of the full 3D spin configuration, future work should employ tilt-series Lorentz TEM imaging over a wide angular range (e.g., –60° to +60°) combined with tomographic reconstruction* [44-46]."

Comment 4: -Electrical Detection (Read-out) Mechanism
While the "writing" and "manipulation" of skyrmion bags are discussed, the "reading" aspect is entirely absent.
The authors should comment on how these varying topological charges could be detected electrically (e.g., via the Topological Hall Effect or TMR) without relying on electron microscopy, which is crucial for any practical spintronic device.

Response: Skyrmion bags with different Q values can be distinguished electrically via the topological Hall effect (THE), whose magnitude is proportional to the net topological charge within the detection region. For more efficient and high-sensitivity detection, we propose using tunnel magnetoresistance (TMR) effects. TMR devices—such as magnetic tunnel junctions (MTJs)—can be integrated into the device geometry to locally probe the stray field or the spin-polarized tunneling current modulated by the skyrmion bag. The resistance change across an MTJ depends on the relative orientation of the free and reference layers; a passing skyrmion bag alters the local magnetization configuration, leading to a distinct resistance signature that scales with Q. This approach has been successfully demonstrated for isolated skyrmions and offers a path toward compact, all-electrical read-out (Ref. 53-55).

We have added a schematic device design in the revised manuscript (Fig. R28), showing a skyrmion bag embedded within a detection region defined by a TMR nanopillar or a Hall cross. We have added comments on the electrical detection of the skyrmion bag in our revised manuscript, see Lines 348-355: "*The electrical detection of skyrmion bags with different topological charges Q can be achieved via the topological Hall effect, whose magnitude is proportional to the Q value within the detection region* [50-52]*. For more efficient and practical read-out, tunnel magnetoresistance effects—integrated into a magnetic tunnel junction geometry—can be used to distinguish different Q values of skyrmion bags (Supplementary Fig. 23d)* [53-55]*. This provides a pathway for all-electrical writing, manipulation, and reading of skyrmion-bag-based spintronic devices.*"

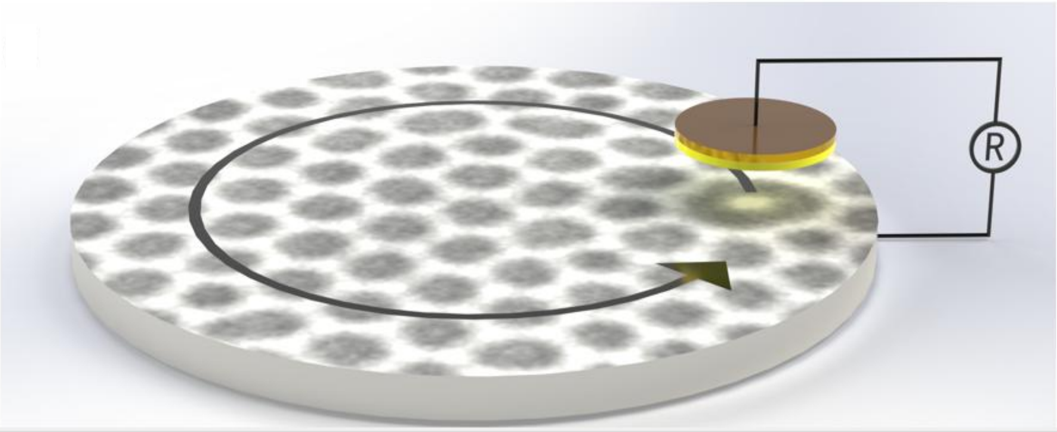

Fig. R28 (*i.e.*, Supplementary Fig. 23) Schematic device design based on embedded skyrmion bags.

Comment 5: -Material and Temperature Generality
The experiments are conducted on FeGe at specific temperatures (around 220 K).
The authors should address whether the observed current-induced bag formation is a universal phenomenon applicable to room-temperature chiral magnets or multilayer systems with interfacial Dzyaloshinskii-Moriya Interaction (DMI).

Response: We thank you very much for this important question regarding the generality of our findings. Below, we provide a detailed response.

Mechanism is topology-driven, not material-specific. The formation of skyrmion bags in our experiments relies on three key ingredients: (1) the coexistence of skyrmions with opposite topological charges ($Q = 1$ and $Q = -1$) at zero magnetic field, (2) the instability of a vertical helix end (topologically equivalent to a meron with $| Q |= 1/2$) under spin-transfer torque, and (3) the skyrmion Hall effect, which drives the transverse motion of skyrmions and merons.

These ingredients are not unique to FeGe. The coexistence of opposite-polarity skyrmions at zero field is applicable for other chiral magnets and multilayers with interfacial DMI. The meron-end instability is a generic consequence of the topological nature of the helix termination, independent of the specific exchange or DMI constants. The skyrmion Hall effect is a universal feature of any topologically nontrivial spin texture under a current.

Therefore, the creation mechanism we report is not tied to the specific magnetic parameters of FeGe; rather, it follows from general topological and dynamical principles. In principle, our experimental observations can be extended to other skyrmion-hosting materials, including room-temperature chiral magnets and multilayers with interfacial Dzyaloshinskii-Moriya Interaction.

We have clarified this point in the revised manuscript (Lines 227–233): "*The creation mechanism of skyrmion bags relies on the coexistence of skyrmions with opposite topological charges at zero magnetic field, and the STT-driven instability of helix ends—both of which are rooted in topology and dynamics rather than in specific material parameters. Therefore, our experimental findings can, in principle, be generalized to other skyrmion hosting systems, including room temperature chiral magnets and multilayers with interfacial DMI* [14]."

Comment 6: -The stability and retention time of the generated skyrmion bags after the current is turned off should be quantified.

Response: Following your comment, we have added the stability of skyrmion bags after their formation. Under constant physical conditions (i.e., without changing temperature, magnetic field, or applied current), we monitored the skyrmion bags over time. Our experiments reveal that the skyrmion bags exhibit no observable variation in their morphology, size, or position for periods exceeding 13 hours. This indicates that these high-Q spin textures are remarkably stable at zero external magnetic field once formed.

We have added this important finding to the revised manuscript (see Lines 278–283): "*To assess the stability of skyrmion bags, we monitored the magnetic texture under constant conditions (zero applied current, 95 K, and zero external magnetic field) for over 13 hours (Supplementary Fig. 18). Repeated imaging reveals no detectable changes in the morphology, size, or position of the skyrmion bags, demonstrating their long-term stability.*"

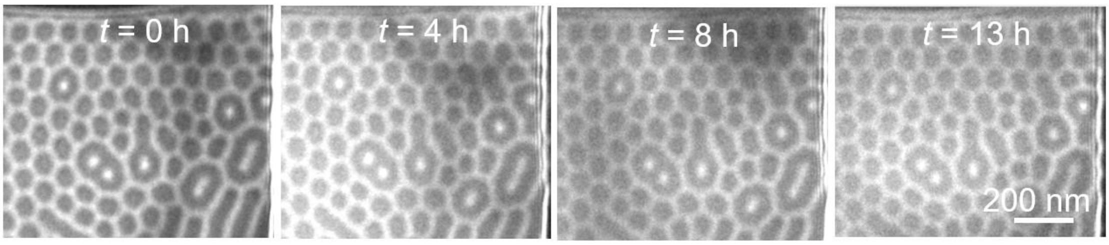

Fig. R29 (*i.e.*, Supplementary Fig. 18) Skyrmion bag stability over time under constant conditions (zero applied current, 95 K, and zero external magnetic field).

Manuscript ID: NCOMMS-25-94549B

"*Current-induced creation and dynamics of embedded magnetic skyrmion bags*" by Y. Wu *et al.*

Note that all page numbers and references without special instructions refer to the newly revised manuscript attached with this response and not to the original version submitted.

Response to Reviewer #1:

Reviewer #1: I am satisfied with the authors responses to my queries. I have also noticed that the authors have made appropriate changes in the revised manuscript. I believe the manuscript is now suitable for publication.

Response: Thank you for your positive assessment and for your time and expertise in reviewing our manuscript. We are delighted to know that you support its publication. Your insightful comments and constructive suggestions have greatly helped us improve the quality of our work. We are sincerely grateful for your efforts and valuable contributions.

Response to Reviewer #2:

Reviewer #2: The authors have provided detailed rebuttals to all the criticisms raised by the three referees, and introduced the corresponding revisions in the manuscript. In my opnion, these rebuttals/changes are thorough and convincing. As I wrote in my original review, as far as the field of skyrmionics is concerned, the manuscript provides a sizable contribution and will likely be well-received. I believe it should be published.

However I should note that the comments of the reviewers and the discussion in the manuscript seem to be based on the implicit assumption that there are widely accepted pathways for practical applications of skyrmionic devices that can materialize once cetain conditions such as room-temperature operation mentioned by the Referee C, or perhaps some stability/efficiency conditions mentioned by Referee A, are satisfied. However, I am personally not aware of any concrete proposals for such practical devices, or estimates of how they would compete with existing technologies, and neither have I seen any substantial analysis of what properties, if any, of skyrmion-hosting materials would satisfy the application requirements.

Response: We sincerely thank you for your thorough review and for recommending our manuscript for publication. We greatly appreciate your insightful comment regarding the practical application landscape of skyrmionic devices. You raise an important point—the current gap between fundamental discoveries and concrete, competitive device proposals is indeed a challenge that the field must address moving forward. We fully agree with your perspective, and we are grateful for your continued support and valuable time dedicated to improving our work.
